# Supplementary material for: Patient-reported outcome measures for patients with meniscal tears: a systematic review of measurement properties and evaluation with the COSMIN checklist
Source: BMJ Open. 2017 Oct 13;7(10):e017247. doi: 10.1136/bmjopen-2017-017247 (PMC5652504; doi:10.1136/bmjopen-2017-017247)
Supplement: Supplementary Appendix 1 [file bmjopen-2017-017247supp001.pdf]

Appendix 1: Search strategy

Databases: MEDLINE, Embase, AMED, PsycInfo

#1 Condition

menis*.af

#2 Construct

("quality of life" OR qol OR func* OR HR-PRO OR HRPRO OR HRQOL OR QL OR disab* OR wellbeing OR "well being" OR subjective OR utility OR utilities OR priorit* OR outcome* OR health).af

#3 Instrument

(score* OR measure* OR PROM OR index* OR indices OR scale* OR questionnaire* OR instrument* OR survey* OR profile* OR apprais* OR status OR reported OR reporting OR rated OR rating* OR assessment*).af

#4 Measurement Properties

"Validation Studies".pt OR instrumentation.af OR ("observer variation" OR "psychometrics" OR "reproducibility of results" OR "discriminant analysis").mh OR (agreement OR precision OR imprecision OR "precise values" OR repeatab* OR ((replicab* OR repeated) AND (measure OR measures OR findings OR result OR results OR test OR tests))).af OR (reproducib* OR psychometr* OR clinimetr* OR clinometr* OR observer AND variation OR reliab* OR valid* OR coefficient OR "internal consistency" OR (cronbach* AND (alpha OR alphas)) OR "item correlation" OR "item correlations" OR "item selection" OR "item selections" OR "item reduction" OR "item reductions" OR test?retest OR (test AND retest) OR (reliab* AND (test OR retest)) OR stability OR interrater OR inter-rater OR intrarater OR intra-rater OR intertester OR inter-tester OR intratester OR intra-tester OR interobserver OR inter-observer OR intraobserver OR intra-observer OR intertechnician OR intertechnician OR intratechnician OR intra-technician OR interexaminer OR inter-examiner OR intraexaminer OR intra-examiner OR inter-assay OR inter-assay OR intraassay OR intra-assay OR interindividual OR inter-individual OR intraindividual OR intra-individual OR interparticipant OR inter-participant OR intraparticipant OR intra-participant OR kappa OR kappa?s OR "coefficient of variation" OR generaliza* OR generalisa* OR concordance OR (intraclass AND correlation*) OR discriminative OR "known group" OR "factor analysis" OR "factor analyses" OR "factor structure" OR "factor structures" OR dimensionality OR subscale* OR "multitrait scaling analysis" OR "multitrait scaling analyses" OR "item discriminant" OR "interscale correlation" OR "interscale correlations" OR ((error OR errors) AND (measure* OR correlat* OR evaluat* OR accuracy OR accurate OR precision OR mean)) OR "individual variability" OR "interval variability" OR "rate variability").ti,ab NOT ("addresses" OR "biography" OR "case reports" OR "comment" OR "directory" OR "editorial" OR "festschrift" OR "interview" OR "lectures" OR "legal cases" OR "legislation" OR "letter" OR "news" OR "newspaper article" OR "patient education handout" OR "popular works" OR "congresses" OR "consensus development conference" OR "consensus development conference, nih" OR "practice guideline").pt

#5 (#1 AND #2 AND #3 AND #4)

#5 Remove Duplicates
