# Supplementary material for: Patient-reported outcome measures for patients with meniscal tears: a systematic review of measurement properties and evaluation with the COSMIN checklist
Source: BMJ Open. 2017 Oct 13;7(10):e017247. doi: 10.1136/bmjopen-2017-017247 (PMC5652504; doi:10.1136/bmjopen-2017-017247)
Supplement: Supplementary Appendix 2 [file bmjopen-2017-017247supp002.pdf]

Appendix 2: Quality criteria for measurement properties[1]

Property	Rating	Quality Criteria
**Reliability**		
Internal consistency	+	Cronbach's alpha(s) ≥ 0.70
	?	Cronbach's alpha not determined or dimensionality unknown
	-	Cronbach's alpha(s) < 0.70
Reliability	+	ICC / weighted Kappa ≥ 0.70 OR Pearson's r ≥ 0.80
	?	Neither ICC / weighted Kappa, nor Pearson's r determined
	-	ICC / weighted Kappa < 0.70 OR Pearson's r < 0.80
Measurement error	+	MIC > SDC OR MIC outside the LOA
	?	MIC not defined
	-	MIC ≤ SDC OR MIC equals or inside LOA
**Validity**		
Content validity	+	All items are considered to be relevant for the construct to be measured, for the target population, and for the purpose of the measurement AND the questionnaire is considered to be comprehensive
	?	Not enough information available
	-	Not all items are considered to be relevant for the construct to be measured, for the target population, and for the purpose of the measurement OR the questionnaire is considered not to be comprehensive
Construct validity – Structural validity	+	Factors should explain at least 50% of the variance
	?	Explained variance not mentioned
	-	Factors explain < 50% of the variance
Construct validity – Hypothesis testing	+	Correlations with instruments measuring the same construct ≥ 0.50 OR at least 75% of the results are in accordance with the hypotheses AND correlations with related constructs are higher than with unrelated constructs
	?	Solely correlations determined with unrelated constructs
	-	Correlations with instruments measuring the same construct < 0.50 OR < 75% of the results are in accordance with the hypotheses OR correlations with related constructs are lower than with unrelated constructs
Construct validity – Cross-cultural validity	+	No differences in factor structure OR no important DIF between language versions
	?	Multiple group factor analysis not applied AND DIF not assessed
	-	Differences in factor structure OR important DIF between language versions
**Responsiveness**		
Responsiveness	+	Correlation with changes on instruments measuring the same construct ≥ 0.50 OR at least 75% of the results are in accordance with the hypotheses OR AUC ≥ 0.70 AND correlations with changes in related constructs are higher than with unrelated constructs
	?	Solely correlations determined with unrelated constructs
	-	Correlations with changes on instruments measuring the same construct < 0.50 OR < 75% of the results are in accordance with the hypotheses OR AUC < 0.70 OR correlations with changes in related constructs are lower than with unrelated constructs

MIC = minimal important change, SDC = smallest detectable change, LoA = limits of agreement, ICC = intraclass correlation coefficient, DIF = differential item functioning, AUC = area under the curve
+ = positive rating, ? = indeterminate rating, - = negative rating

1 Terwee CB, Bot SDM, de Boer MR, *et al.* Quality criteria were proposed for measurement properties of health status questionnaires. *J Clin Epidemiol* 2007; **60**: 34–42.
