# Supplementary material for: Patient-reported outcome measures for patients with meniscal tears: a systematic review of measurement properties and evaluation with the COSMIN checklist
Source: BMJ Open. 2017 Oct 13;7(10):e017247. doi: 10.1136/bmjopen-2017-017247 (PMC5652504; doi:10.1136/bmjopen-2017-017247)
Supplement: Author's manuscript [file bmjopen-2017-017247.draft_revisions.pdf]

BMJ Open is committed to open peer review. As part of this commitment we make the peer review history of every article we publish publicly available.

When an article is published we post the peer reviewers' comments and the authors' responses online. We also post the versions of the paper that were used during peer review. These are the versions that the peer review comments apply to.

The versions of the paper that follow are the versions that were submitted during the peer review process. They are not the versions of record or the final published versions. They should not be cited or distributed as the published version of this manuscript.

BMJ Open is an open access journal and the full, final, typeset and author-corrected version of record of the manuscript is available on our site with no access controls, subscription charges or pay-per-view fees (http://bmjopen.bmj.com).

If you have any questions on BMJ Open's open peer review process please email editorial.bmjopen@bmj.com

BMJ Open

Patient-reported outcome measures for patients with meniscal tears: a systematic review of measurement properties and evaluation with the COSMIN checklist

Journal:	*BMJ Open*
Manuscript ID	bmjopen-2017-017247
Article Type:	Research
Date Submitted by the Author:	11-Apr-2017

Complete List of Authors:	Abram, Simon; University of Oxford Nuffield Department of Orthopaedics Rheumatology and Musculoskeletal Sciences,<br>Middleton, Robert; University of Oxford Nuffield Department of Orthopaedics Rheumatology and Musculoskeletal Sciences<br>Beard, David; Oxford University, Nuffield Dept of Orthopaedics, Rheumatology and Musculoskeletal Sciences<br>Price, Andrew; University of Oxford, Nuffield Department of Orthopaedics, Rheumatology and Musculoskeletal Sciences<br>Hopewell, Sally; University of Oxford, Oxford Clinical Trials Research Unit

**Primary Subject Heading**:	Surgery
Secondary Subject Heading:	Sports and exercise medicine, Patient-centred medicine
Keywords:	Knee < ORTHOPAEDIC & TRAUMA SURGERY, ORTHOPAEDIC & TRAUMA SURGERY, Orthopaedic & trauma surgery < SURGERY

For peer review only

SCHOLARONE™ Manuscripts

Patient-reported outcome measures for patients with meniscal tears: a systematic review of measurement properties and evaluation with the COSMIN checklist

S.G.F. Abram, R. Middleton, D.J. Beard, A.J. Price, S. Hopewell.

Authors' affiliation:

Nuffield Department of Orthopaedics, Rheumatology and Musculoskeletal Sciences, University of Oxford

Corresponding author:

Simon Abram

simon.abram@doctors.org.uk

Keywords:

outcomes; patient reported outcomes; psychometrics; meniscal pathology; meniscus; knee surgery; knee

For peer review only

Abstract

Objective: Meniscal tears occurs frequently in the population and an estimated 2 million arthroscopic meniscectomy procedures are performed worldwide each year. The purpose of this systematic review is to summarise and critically appraise the evidence for patient-reported outcome measures (PROMs) in patients with meniscal tears.

Design: A systematic review was undertaken. Data on reported measurement properties was extracted and the quality of the studies appraised according to Consensus-based Standards for the selection of health Measurement Instruments (COSMIN).

Data sources: A search of Medline, EMBASE, AMED and PsycINFO, unlimited by language or publication date (last search 20/02/17).

Eligibility criteria for selecting studies: Development and validation studies reporting the measurement properties of PROMs in patients with meniscal tears were included.

Results: 11 studies and 10 PROMs were included. The overall quality of studies was poor. For measurement of symptoms and functional status there is only very limited evidence supporting the selection of either the Lysholm knee scale, International Knee Documentation Committee (IKDC) Subjective Knee Form, or the Dutch-version of the Knee Osteoarthritis Outcome Score (KOOS). For measuring health-related quality of life, only limited evidence supports the selection of Western Ontario Meniscal Evaluation Tool (WOMET). Of all the PROMs evaluated, WOMET has the strongest evidence for content validity.

Conclusion: For patients with meniscal tears, there is poor quality and incomplete evidence regarding the validity of the currently available PROMs. Clinical trials and other studies reliant on these PROMs should, therefore, be interpreted with caution.

PROPERO registration number: CRD42017056847

For peer review only

Strengths and limitations of this study

- This is the first review of PROMs for patients with meniscal tears and the first to apply the COSMIN checklist, which is a validated and accepted tool for the appraisal of study quality.
- Although COSMIN has acceptable inter-rater and intra-rater properties, the scoring of some items is reliant on author judgement. We performed pre-testing to ensure scoring consistency and review authors scored studies independently. Nevertheless, it is feasible that another review team might score some items differently.
- One strength of this review is the use of a validated, highly-sensitive search strategy to identify relevant studies. A limitation, however, is that only studies specifically designed to appraise the measurement properties of PROMs were included. Trials and other clinical studies of patients with meniscal tears were not included as these studies are not designed to assess measurement properties and the reporting of these properties would be highly unusual.
- For practical purposes, we chose to include tentative summary guidance regarding the selection of PROMs for use in the target population. It should be understood, however, that it would be reasonable to declare that the overall level of evidence for any of the PROMs is insufficient for a recommendation to be made.

For peer review only

Introduction

The menisci are fibrocartilaginous structures within the knee joint which are important for load distribution and knee stability.[1,2] More than one third of people over the age of 50 without any radiographic evidence of osteoarthritis may develop a tear of the meniscus and over 70% of those with osteoarthritis will also have meniscal pathology.[3] These meniscal tears may be associated with significant knee pain.[4] Arthroscopic meniscectomy is a surgical procedure commonly used to treat symptomatic meniscal tears with approximately two million cases performed worldwide each year with combined costs of several billion US dollars.[5] A number of recent randomised controlled trials have been published challenging the effectiveness of arthroscopic meniscectomy.[6–10] The published trials have used a wide array of patient-reported outcome measures (PROMs) and this inconsistency leads to restricted comparisons between trials and difficult interpretation of their findings.[11] The best PROM for this population is unknown.

Patient reported outcome measures (PROMs) are collected in a range of settings and are increasingly important in clinical practice. In orthopaedics, PROMs are important for auditing treatment outcomes and increasingly to demonstrate the cost-effectiveness of treatment.[12] With the rapid increase in usage, it is important to ensure that PROMs are, as much as possible, condition-specific and formally validated for the 'construct' to be measured; for example, health-related quality of life in patients with meniscal tears.[13] Ideally, for a PROM to be valid, it should be developed with condition-specific patient involvement. In other cases, a PROM may be developed for an alternative population of patients and subsequently studied for validity in the target population with the condition of interest.

There is a need for the selection of condition-specific, standardised, 'core' outcome sets for use in clinical trials and general clinical evaluation.[14] A systematic review of the evidence is an important step in the selection of such a core outcome PROM and may determine the need for further validation studies or even the development of a new PROM.[14] No systematic review has been published evaluating the measurement properties with the quality of evidence for the PROMs that are available for patients with meniscal tears. This is a barrier to the interpretation of previous research and to the design of future studies in these patients.

The purpose of this review is to report the measurement properties and evidence for the validity of all PROMs which have been evaluated in patients with meniscal tears.

For peer review only

Methods

Search Strategy

We performed a search of Medline, EMBASE, AMED and PsycINFO, unlimited by language or publication date. The search was based upon a validated search filter designed to be highly sensitive in identifying all studies of measurement properties.[15] Full details of the search are available in supplementary appendix 1. The final search was performed on 20/02/2017 following submission of the protocol to PROSPERO (CRD42017056847). A review of study citations was performed to further increase the sensitivity of the search strategy.

Selection of studies

The title and abstract of all records retrieved by the search were independently reviewed by two authors against the inclusion and exclusion criteria (SA and RM). Any disagreement was resolved with review of the full text publication and discussion. Referral to a third author (SH) was not required for agreement.

Development and validation studies for PROMs reporting measurement properties in patients with meniscal tears were included. PROMs aiming to measure health related quality of life, health status, symptoms including pain, or functional status were included. PROMs using standard scoring methods and without clinician completed elements were included and only studies involving discrete populations of patients with meniscal tears. Those studies with less than 50% of patients having a meniscal tear as the primary diagnosis (i.e. without other significant knee pathology e.g. concomitant anterior cruciate ligament (ACL) rupture) were excluded unless the meniscal tear group were reported separately.

Where a PROM was developed for a condition other than meniscal pathology and subsequently tested in a population with meniscal tears, the full text original development article for the included PROM was identified and reviewed.

Data extraction: Measurement properties and assessing the quality of studies

Data extraction was performed by two authors (SA and RM) and any disagreement resolved in consultation with a third author (SH). The following was extracted from each publication: the PROM, the intended construct for measurement, measurement properties, administration method, study population and diagnosis, number of patients, patient demographics, country, language and setting and method of administration (e.g. postal, online).

The quality of each included studies was assessed by two reviewers (SA and RM) using the Consensus-based Standards for the selection of health Measurement Instruments (COSMIN) appraisal checklist.[16] When reviewing a study of a PROM, it is necessary to consider a combination of the reported measurement properties, the patient

For peer review only

population, and the quality of the study methodology. To help overcome some of the difficulties in evaluating the quality of PROMs, COSMIN was published in 2010.[17,18] COSMIN contains rules for grading overall methodological quality of studies performed into the measurement properties of PROMs. These consensus standards are regularly reviewed and revised based on the latest evidence and research. COSMIN initially separated standards into boxes including a series of binary methodological ratings. The scoring methodology was subsequently revised to a four level (excellent/good/fair/poor) rating system in 2012.[16] Each measurement property is assessed by a box containing 5-18 questions scored on this scale according to defined COSMIN criteria. A system of 'worst score counts' applies for each box – that is, if one question in the box is scored as poor, the overall quality of the evidence for that measurement property is determined to be poor. COSMIN have also published agreed definitions for each measurement property as detailed below.[19] All measurement properties reported by the included studies were evaluated.

Reliability

Overall, reliability is a measure of how free a PROM is from measurement error.[19] Reliability is assessed by collecting the PROM twice in a defined period when there has been no change in the patient's condition. Ideally, rather than assume the patient's condition is unchanged, a methodologically strong study will confirm this, for example by administering a knee-specific global transition question on symptoms.

- <u>Internal consistency:</u> is the degree of inter-relatedness among the PROM items.[19]
- <u>Reliability:</u> is the proportion of total variance in the measurement which is because of true differences among patients.[19]
- <u>Measurement error:</u> is the systematic and random error of a patient's score that is not attributed to true changes in the construct to be measured.[19]

Validity

Validity is the extent to which the PROM measures the 'construct' it purports to measure.[19]

- <u>Content validity:</u> is the degree to which the content of a PROM is an adequate reflection of the construct to be measured.[19]
- <u>Construct validity:</u> is the degree to which the scores of a PROM are consistent with hypotheses (e.g. relationships of score to other instruments or differences between relevant groups) based on the assumption the PROM validly measures the intended construct.[19]
    - <u>Structural validity:</u> is the degree to which the scores of a PROM are an adequate reflection of the dimensionality of the construct to be measured.[19]
    - <u>Hypothesis testing:</u> assumes that the PROM validly measures the construct of interest. Hypotheses are prepared *a priori* with regards to the correlation of the PROM with other relevant PROMs or domains of other PROMs. The magnitude and direction of the correlation should be stated in advance of testing.

For peer review only

- Cross-cultural validity: is the degree to which the performance of the items on a translated or culturally adapted PROM are comparable to the performance of the original version of the PROM.[19]

Responsiveness

Responsiveness is defined as the ability of a PROM to measure change over time in the construct to be measured.[19] It is important to note that in studies assessing the measurement properties of a PROM, responsiveness should be assessed against another valid PROM as for the assessment of construct validity. Measurement of effect size alone is not appropriate as this is a measure of the magnitude of the change and not the quality of the measurement.[17]

Interpretability

Interpretability is defined by COSMIN as the degree to which it is possible to assign qualitative meaning to a PROM's quantitative score.[19] It is not considered a measurement property but is important when interpreting the findings from administration of a PROM in the context of a clinical condition. Interpretability includes an assessment of minimal important change (MIC), floor and ceiling effects. In general, floor and ceiling effects <15% are considered acceptable although some authors have argued the threshold should be set higher at <30%.[20,21] A high floor or ceiling effect suggests that items at the lower or upper end are missing from a question item, domain, or the PROM overall.

Generalisability

Generalisability is an assessment of external validity: the extent to which the findings on the measurement properties of a PROM may be considered relevant to a population or construct or interest. For example, a study of the measurement properties of a PROM in a population with advanced knee osteoarthritis cannot be generalised to athletes with knee ligament injury without further study in the target population. In this review, the population of patients involved in the original development of each PROM is determined and the inclusion and exclusion criteria of all studies reporting measurement properties of the included PROMs is reported. This enables the generalisability of findings to the population of patients with meniscal tears to be considered.

Data synthesis

Data synthesis was performed by SA and checked by RM. For each included PROM, a summary of the features of the PROM is presented including details of the original development process, the development population and target construct to be measured.

For each PROM, a rating (positive, negative or indeterminate) for the measurement properties reported in the study was first determined based upon consensus standards described in supplementary appendix 2.[21] This assessment

For peer review only

was then combined with an overall quality of evidence assessment which was adapted for COSMIN from the work of the Cochrane Back Review Group (Table 1).[22,23] For example, one good quality study reporting positive measurement properties (e.g. internal consistent with Cronbach's alpha ≥ 0.70) results in an overall rating of 'moderate' (++). Where the quality of study methodology on a measurement property is rated 'poor' the overall rating of the measurement property is always rated 'indeterminate', irrespective of the number of such studies and whether the reported measurement property itself would otherwise be considered positively. These standards are designed to ensure reported measurement properties are interpreted in the context of study quality and overall reliability.

For peer review only

Results

Selection of studies

The search strategy identified 1321 unique articles for screening. After screening, 34 full text articles were retrieved of which 11 met the inclusion criteria for this review. Figure 1 summarises the study selection process. The 11 studies reported measurement properties for 10 PROMs. The characteristics of the included studies are shown in Table 2 and the development and features of the included PROMs are summarised in Table 3.

Quality of the included studies

In total, the 11 studies reported 93 measurement properties for the 10 PROMs. The COSMIN methodology rating for 49 of these (53%) was poor. Many measurement properties were not reported and there was inconsistent reporting between studies (Table 5).

Quality of PROMs

Interpretability factors including floor and ceiling effects are summarised in Table 4. The overall level of evidence for the measurement properties of each is PROM is summarised in Table 6. This combines the rating of the reported measurement property using the consensus criteria available in supplementary appendix 2 with the COSMIN scoring and the number of studies per PROM (as described in Table 1).

Of the 10 PROMs identified, five intended to measure symptoms and functional status, four health-related quality of life and one activity level.

Symptoms & Functional Status

Hughston: The Hughston Clinic Questionnaire was developed in 1991 as a knee-specific rather than disease-specific outcome measure.[24] It includes questions on symptoms, functional status and sports activity and patients were not involved in the development of the questions. Only one study has evaluated use of the Hughston questionnaire in patients with meniscal tears.[25] Content validity was rated poor as patients were not involved in the original development and content validity has not been subsequently assessed in patients with meniscal tears. In patients with meniscal tears, there was moderate negative evidence against construct validity based on hypothesis testing and all other measurement properties were either not reported or indeterminate due to poor study design or reporting (Table 6).

IKDC: The International Knee Documentation Committee (IKDC) Subjective Knee Form was developed in 2001 as a knee-specific rather than disease-specific outcome measure.[26] It includes question domains on symptoms, functional

For peer review only

status and sports activity and patients were not involved in the development of the questions. Two studies have evaluated use of the IKDC score in patients with meniscal tears.[27,28] In English there is limited positive evidence for reliability and construct validity based on hypothesis testing.[27] In Dutch, there is moderate positive evidence for reliability and construct validity based on hypothesis testing but limited negative evidence against structural validity.[28] In both studies, all other measurement properties were either not reported or indeterminate (Table 6). For the English-version, although no floor or ceiling effect was detected for the overall score, unacceptable floor effects were reported for 9 items and unacceptable ceiling effects in 5 items (Table 4).

KOOS: The Knee Outcome Osteoarthritis Score (KOOS) was developed in 1998 as a knee-injury specific outcome measure for patients at risk of developing osteoarthritis.[29] It includes question domains on symptoms, functional status, sports activity and quality of life. Patients with anterior cruciate ligament (ACL) or meniscal injuries were included in the development process. The KOOS includes the WOMAC osteoarthritis score in full and the WOMAC may therefore be calculated from the KOOS. The KOOS has been studied in Dutch and Swedish for patients with meniscal tears; no study has evaluated the English-version of KOOS in this population.[28,30] There is moderate positive evidence for reliability and construct validity from hypothesis testing of the Dutch-version.[28] For the Swedish-version, there is limited positive evidence for reliability and construct validity based on hypothesis testing.[31] For both the Dutch and Swedish versions, content validity and all other measurement properties were either rated indeterminate or were not reported (Table 6).

Lysholm: The Lysholm knee score was developed in 1982 and modified in 1985 as a disease-specific outcome measure for patients with knee ligament injury.[32,33] The Lysholm knee score was originally designed to be completed by clinicians and developed without patient involvement. One study has evaluated the use of Lysholm in English speaking patients with meniscal tears.[34] There is limited positive evidence for reliability and construct validity based on hypothesis testing. Content validity and all other measurement properties are either indeterminate or were not reported (Table 6). There was no floor or ceiling effect for the Lysholm score overall however an unacceptable floor effect was detected for 2 items and unacceptable ceiling effects for 5 items (Table 4).

WOMAC: The Western Ontario McMaster Osteoarthritis Index (WOMAC) was developed in 1982 as a disease-specific outcome measure for patients with osteoarthritis of the hip or knee.[35] The WOMAC includes question domains for pain, stiffness and functional status and patients with osteoarthritis were involved in the development of the questions. The WOMAC is incorporated in its entirety in the KOOS (see above). One study has evaluated the Dutch version of WOMAC in patients with meniscal tears.[28] In these patients, there is moderate positive evidence for reliability and construct validity (hypothesis testing). No floor or ceiling effects were detected. Content validity and all other measurement properties are either indeterminate or were not reported (Table 6).

Health-related quality of life

For peer review only

EQ-5D: EQ-5D is a generic measure of health-related quality of life developed in 1990.[36] It was developed with patient involvement and includes question domains on mobility, self care, usual activities, pain, and anxiety or depression. One study has evaluated the English EQ-5D in patients with meniscal tears.[25] In this population, there is moderate positive evidence for construct validity based on hypothesis testing. All other measurement properties are either indeterminate or were not reported.

KQoL-26: The Knee Quality of Life (KQoL-26) 26-item questionnaire was developed in 2008, in English, as a disease-specific health-related quality of life measure for patients with suspected ligamentous or meniscal injury of the knee.[37] In the study population, 67% of patients had a meniscal tear and there is limited positive evidence for internal consistency, reliability, content validity, and construct validity (hypothesis testing and structural validity). Administered by post, an overall response rate of 59% was reported with 14.9% missing items.[37] Floor and ceiling effects were poorly reported with at least one question having an unacceptable floor effect and one an unacceptable ceiling effect (Table 4).

SF-6D: The short form-6 dimensions (SF-6D) generic health-related quality of life measure is derived from the SF-36 or SF-12 and was developed in 2004.[38] It was developed with patient involvement and contains 6 questions domains: physical functioning, role limitation, social functioning, pain, mental health and vitality. One study has evaluated the English version of SF-6D in patients with meniscal tears.[25] There is moderate positive evidence for construct validity based on hypothesis testing but all other measurement properties are indeterminate or were not reported.

WOMET: The Western Ontario Meniscal Evaluation Tool (WOMET) is a meniscal tear disease-specific, quality of life measure developed in 2007.[39] Patients with meniscal tears were involved throughout the development process although the authors reported that the same patients were “admittedly heterogeneous with respect to the incidence of coexisting knee pathology such as chondral damage or ligament injury”.[39] The WOMET has been evaluated in English, Chinese, Dutch, Finnish and Turkish. There is strong positive evidence for content validity in the English version and moderate positive evidence in the Dutch version. There is limited positive evidence for reliability, construct validity (hypothesis testing) and responsiveness of the English-version.[40] Measurement error was only reported for the Dutch-version of WOMET and in this case it was concerning that the minimal important change (MIC) for the PROM was found to be less than the smallest detectable change (SDC). A summary of the level of evidence for the measurement properties in all languages is shown in Table 6. Although the overall score does not exhibit floor or ceiling effects, unacceptable levels were reported for several items (Table 4).

Activity Level

Tegner: The Tegner Activity Scale was developed in 1985 for patients with ACL injury.[33] Patients were not involved in the development of the scale. One study has evaluated use of the scale in patients with meniscal tears.[34] In this population, there is limited positive evidence for reliability and construct validity based on hypothesis testing. All other measurement properties were either not reported or indeterminate.

For peer review only

Discussion

This review identified 11 studies evaluating 10 PROMs in patients with meniscal tears: five PROMS measuring symptoms and functional status, four PROMs measuring health-related quality of life and one for activity level. Unfortunately, the findings of the studies were limited by poor methodology and incomplete reporting of PROM measurement properties.

One previous review has been published summarising reported measurement properties of a range of PROMs in studies of patients with any knee condition.[41] In this previous review, WOMET was broadly recommended for use in patients with meniscal injuries without distinguishing the intended health-related quality of life construct from others or assessing the quality of the studies.[41] Ours is the first systematic review of PROMs for patients with meniscal tears and the first to evaluate and report the quality of study methodology. In orthopaedics and sports medicine, systematic reviews of PROMs applying the COSMIN appraisal checklist are established and have been published for patient populations including those with hip and knee osteoarthritis, hip and groin disability, patellofemoral pain, distal radius fractures, shoulder pain, and undergoing hip arthroscopy.[42–49]

For studies included in this review, the COSMIN methodology rating was poor for just over half (53%) of reported measurement properties. Internal consistency was rated poor in all but one of the 11 studies. A key reason for this was the failure of most studies to perform factor analysis to assess the structural validity of PROMs. Internal consistency is an assessment of the inter-relatedness of the items measuring the same underlying construct i.e. the PROM or sub-domain should be 'unidimensional' for the construct to be measured. Factor analysis is a technique that may be used determine whether a PROM or sub-domain is 'unidimensional'. Without this assessment of structural validity, there can be no clear interpretation of internal consistency statistics.[17]

Cross-cultural validity and responsiveness were also particularly poorly evaluated. Regarding responsiveness, frequently studies reported only an effect size for the studied PROM. Effect size alone is measure of the magnitude of a change scores and not the quality of the measurement and is therefore insufficient to assess this measurement property.[17] Responsiveness refers to the validity of a change score and should be assessed with, for example, hypothesis testing against the change score of another related PROM, analogous to the assessment of construct validity.

Measurement error was poorly reported in the included studies and the minimal important change (MIC) was calculated for only one of the PROMS – the Dutch-version of WOMET.[50] It was concerning that in this case the MIC was found to be less than the smallest detectable change (SDC) due to measurement error. Failure to determine and report this information affects the ability of researchers to design high-quality prospective studies and limits interpretation of previous work.

For peer review only

Evidence for the content validity of the available PROMs was limited. Only the KQoL-26 and WOMET were developed with involvement from patients with meniscal tears. Overall, there was heterogeneity in the population of the patients recruited to the included studies as shown in Table 2. Although most patients in the included studies had meniscal tears as their primary diagnosis, many also had a diagnosis of ligament injury or chondral damage. This reflects the heterogeneity of patients with meniscal tears in general, ranging from the isolated traumatic tear in a young athlete without osteoarthritis to atraumatic tears in older patients with osteoarthritis. This makes work developing and evaluating PROMs in patients with meniscal tears particularly challenging but necessary to inform the design and interpretation of clinical studies of treatments such as arthroscopic meniscectomy.

Strengths and limitations

One strength of this review is the use of a validated, highly-sensitive search strategy to identify relevant studies.[15] A limitation, however, is that only studies specifically designed to appraise the measurement properties of PROMs were included. Trials and other clinical studies of patients with meniscal tears were not included as these studies are not designed to assess measurement properties and the reporting of these properties would be highly unusual.

This is the first review of PROMs for patients with meniscal tears and the first to apply the COSMIN checklist, which is a validated and accepted tool for the appraisal of study quality. Although it has been shown to have acceptable inter-rater and intra-rater properties, the scoring of some items is reliant on author judgement.[51] We performed pre-testing to ensure scoring consistency and review authors scored studies independently with any disagreement being settled by consensus or discussion with a third author. Nevertheless, it is feasible that another review team might score some items differently.

For practical purposes, we chose to include tentative summary guidance regarding the selection of PROMs for use in the target population. It should be understood, however, that it would be reasonable to declare that the overall level of evidence for any of the PROMs is insufficient for a recommendation to be made.

Conclusion

Currently, although a wide range of PROMs are available for patients with knee conditions, the PROMs that have been tested in patients with meniscal tears all lack data on a large proportion of measurement properties. This is disappointing given moves to select condition-specific, standardised, 'core' outcome sets for use in clinical trials and general clinical evaluation.[14] Considerable further work is required before this will be possible for patients with meniscal tears.

For the assessment of symptoms and functional status in patients with meniscal tears, there is currently only very limited evidence supporting the selection of the English-version of Lysholm or IKDC, or Dutch-version of KOOS. Although the total score of these three PROMs does not exhibit floor or ceiling effects, a considerable number of

For peer review only

sub-domain items from both IKDC and Lysholm were reported to have unacceptable floor or ceiling effects. For health-related quality of life, only limited evidence supports the selection of WOMET. One study suggests that measurement error may limit the ability of the WOMET to detect the minimal important change in score for meniscal patients.[50] Several WOMET sub-domain items, but not the total score, have been reported to exhibit unacceptable floor or ceiling effects. For assessment of activity level, only the Tegner activity scale has been evaluated and only very limited evidence is available.

Of all the PROMs evaluated, WOMET has the strongest evidence for content validity. In common with many of the validation studies in this population, however, the included patients frequently had other diagnoses in the same knee such as ligament injuries or chondral defects. This impacts upon the interpretation of clinical evidence in sub-groups of patients that were poorly represented within the development or validation study population. The findings of these validation studies may not be generalizable to such sub-groups and a PROM may fail to detect important clinical differences. Further validation studies may be required in sub-groups or the development of a more specific outcome measure may be necessary.[52] This is pertinent, for example, to current debate about the effectiveness of arthroscopic meniscectomy where there is an increasing focus on certain sub-groups of patients within this highly heterogeneous population.[11,53–55]

In summary, many PROMs have been used in clinical studies of patients with meniscal tears but the overall quality of evidence supporting the validity of these PROMs is poor. Further work is required targeting the deficiencies highlighted by this systematic review.

For peer review only

Statements

Contributorship statement

S. Abram: methodology, study selection, analysis, writing and editing paper.
R. Middleton: study selection, analysis, editing paper.
D.J. Beard: concept, editing paper.
A. J. Price: concept, editing paper.
S. Hopewell: methodology, analysis, editing paper.

Competing interests

No competing interests.

Funding

This research received no specific grant from any funding agency in the public, commercial or not-for-profit sectors.

Data sharing statement

No additional data available.

For peer review only

References

1 Stephen JM, Halewood C, Kittl C, Bollen SR, Williams A, Amis AA. Posteromedial Meniscocapsular Lesions Increase Tibiofemoral Joint Laxity With Anterior Cruciate Ligament Deficiency, and Their Repair Reduces Laxity. *Am J Sports Med* 2016; **44**: 400–8.

2 Rao AJ, Erickson BJ, Cvetanovich GL, Yanke AB, Bach BR, Cole BJ. The Meniscus-Deficient Knee: Biomechanics, Evaluation, and Treatment Options. *Orthop J Sport Med* 2015; **3**: 2325967115611386.

3 Englund M, Guermazi A, Gale D, *et al.* Incidental meniscal findings on knee MRI in middle-aged and elderly persons. *N Engl J Med* 2008; **359**: 1108–15.

4 Roos EM, Roos HP, Ryd L, Lohmander LS. Substantial disability 3 months after arthroscopic partial meniscectomy: A prospective study of patient-relevant outcomes. *Arthroscopy* 2000; **16**: 619–26.

5 Järvinen TLN, Guyatt GH. Arthroscopic surgery for knee pain. *Bmj* 2016; **3934**: i3934.

6 Sihvonen R, Paavola M, Malmivaara A, *et al.* Arthroscopic partial meniscectomy versus sham surgery for a degenerative meniscal tear. *N Engl J Med* 2013; **369**: 2515–24.

7 Katz JN, Brophy RH, Chaisson CE, *et al.* Surgery versus physical therapy for a meniscal tear and osteoarthritis. *N Engl J Med* 2013; **368**: 1675–84.

8 Herrlin S V., Wange PO, Lapidus G, Hållander M, Werner S, Weidenhielm L. Is arthroscopic surgery beneficial in treating non-traumatic, degenerative medial meniscal tears? A five year follow-up. *Knee Surgery, Sport Traumatol Arthrosc* 2013; **21**: 358–64.

9 Yim J-H, Seon J-K, Song E-K, *et al.* A comparative study of meniscectomy and nonoperative treatment for degenerative horizontal tears of the medial meniscus. *Am J Sports Med* 2013; **41**: 1565–70.

10 Kise NJ, Risberg MA, Stensrud S, Ranstam J, Engebretsen L, Roos EM. Exercise therapy versus arthroscopic partial meniscectomy for degenerative meniscal tear in middle aged patients: randomised controlled trial with two year follow-up. *bmj BMJ BMJ* 2016; **354354**: 3740–3740.

11 Monk P, Garfjeld Roberts P, Palmer AJR, *et al.* The Urgent Need for Evidence in Arthroscopic Meniscal Surgery: A Systematic Review of the Evidence for Operative Management of Meniscal Tears. *Am J Sports Med* 2016. DOI:10.1177/0363546516650180.

12 van de Graaf VA, Scholtes VAB, Wolterbeek N, *et al.* Cost-effectiveness of Early Surgery versus Conservative Treatment with Optional Delayed Meniscectomy for Patients over 45 years with non-obstructive meniscal tears (ESCAPE study): protocol of a randomised controlled trial. *BMJ Open* 2016; **6**: e014381.

13 Mokkink LB, Terwee CB, Stratford PW, *et al.* Evaluation of the methodological quality of systematic reviews of health status measurement instruments. *Qual Life Res* 2009; **18**: 313–33.

14 Williamson PR, Altman DG, Blazeby JM, *et al.* Developing core outcome sets for clinical trials: issues to consider. *Trials* 2012; **13**: 132.

15 Terwee CB, Jansma EP, Riphagen II, De Vet HCW. Development of a methodological PubMed search filter for finding studies on measurement properties of measurement instruments. *Qual Life Res* 2009; **18**: 1115–23.

16 Terwee CB, Mokkink LB, Knol DL, Ostelo RWJG, Bouter LM, De Vet HCW. Rating the methodological quality in systematic reviews of studies on measurement properties: A scoring system for the COSMIN checklist. *Qual*

Life Res 2012; **21**: 651–7.

17 Mokkink LB, Terwee CB, Knol DL, *et al.* The COSMIN checklist for evaluating the methodological quality of studies on measurement properties: a clarification of its content. *BMC Med Res Methodol* 2010; **10**: 22.

18 Mokkink LB, Terwee CB, Patrick DL, *et al.* The COSMIN checklist for assessing the methodological quality of studies on measurement properties of health status measurement instruments: An international Delphi study. *Qual Life Res* 2010; **19**: 539–49.

19 Mokkink LB, Terwee CB, Patrick DL, *et al.* The COSMIN study reached international consensus on taxonomy, terminology, and definitions of measurement properties for health-related patient-reported outcomes. *J Clin Epidemiol* 2010; **63**: 737–45.

20 Briggs KK, Kocher MS, Rodkey WG, Steadman JR. Reliability, validity, and responsiveness of the Lysholm knee score and Tegner activity scale for patients with meniscal injury of the knee. *J Bone Joint Surg Am* 2006; **88**: 698–705.

21 Terwee CB, Bot SDM, de Boer MR, *et al.* Quality criteria were proposed for measurement properties of health status questionnaires. *J Clin Epidemiol* 2007; **60**: 34–42.

22 Schellingerhout JM, Verhagen AP, Heymans MW, Koes BW, De Vet HC, Terwee CB. Measurement properties of disease-specific questionnaires in patients with neck pain: A systematic review. *Qual Life Res* 2012; **21**: 659–70.

23 Van Tulder M, Furlan A, Bombardier C, Bouter L. Updated Method Guidelines for Systematic Reviews in the Cochrane Collaboration Back Review Group. *Spine (Phila Pa 1976)* 2003; **28**: 1290–9.

24 Flandry F, Hunt JP, Terry GC, Hughston JC. Analysis of subjective knee complaints using visual analog scales. *Am J Sports Med* 1972; **19**: 112–8.

25 Goodwin C, Ratcliffe J, Morris J, *et al.* Using the knee-specific Hughston Clinic Questionnaire, EQ-5D and SF-6D following arthroscopic partial meniscectomy surgery: A comparison of psychometric properties. *Qual Life Res An Int J Qual Life Asp Treat Care Rehabil* 2011; **20**: 1437–46.

26 Irrgang JJ, Anderson AF, Boland AL, *et al.* Development and validation of the international knee documentation committee subjective knee form. *Am J Sports Med* 2001; **29**: 600–13.

27 Crawford K, Briggs K, Rodkey G, Steadman Richard J. Reliability, validity, and responsiveness of the IKDC score for meniscus injuries of the knee. *Arthroscopy* 2007; **23**: 839.

28 van de Graaf V a, Wolterbeek N, Scholtes V a B, Mutsaerts EL a R, Poolman RW. Reliability and Validity of the IKDC, KOOS, and WOMAC for Patients With Meniscal Injuries. *Am J Sports Med* 2014; **42**: 1408–16.

29 Roos EM, Roos HP, Lohmander LS, Ekdahl C, Beynnon BD. Knee Injury and Osteoarthritis Outcome Score (KOOS)—Development of a Self-Administered Outcome Measure. *J Orthop Sport Phys Ther* 1998; **28**: 88–96.

30 Roos EM, Roos HP, Ekdahl C, Lohmander LS. Knee injury and Osteoarthritis Outcome Score (KOOS)--validation of a Swedish version. *Scand J Med Sci Sports* 1998; **8**: 439–48.

31 E.M. R, H.P. R, C. E, L.S. L. Knee injury and Osteoarthritis Outcome Score (KOOS)--validation of a Swedish version. *Scand J Med Sci Sports* 1998; **8**: 439–48.

32 Lysholm J, Gillquist J. Evaluation of knee ligament surgery results with special emphasis on use of a scoring scale. 1982; **10**: 150–4.

33 Tegner Y, Lysholm J. Rating systems in the evaluation of knee ligament injuries. *Clin Orthop Relat Res* 1985; : 43–9.

34 K.K. B, W.G. R, J.R. S, M.S. K. Reliability, validity, and responsiveness of the Lysholm knee score and tegner activity scale for patients with meniscal injury of the knee. *J Bone Jt Surg - Ser A* 2006; **88**: 698–705.

35 Bellamy N. The WOMAC Knee and Hip Osteoarthritis Indices: Development, validation, globalization and influence on the development of the AUSCAN Hand Osteoarthritis Indices. *Clin Exp Rheumatol* 2005; **23**. DOI:10.1097/01.prs.0000436796.74305.38.

36 Group TE. EuroQol - a new facility for the measurement of health-related quality of life. *Health Policy (New York)* 1990; **16**: 199–208.

37 Garratt M, Brealey S, Robling M, *et al.* Development of the knee quality of life (KQoL-26) 26-item questionnaire: data quality, reliability, validity and responsiveness. *Health Qual Life Outcomes* 2008; **6**: 48.

38 Braizer J, Roberts J, Deverill M. The Estimation of a Preference-Based Measure of Health Form the SF-36. *Journall Heal Econ* 2002; **21**: 271–92.

39 Kirkley A, Griffin S, Whelan D. The development and validation of a quality of life-measurement tool for patients with meniscal pathology: the Western Ontario Meniscal Evaluation Tool (WOMET). *Clin J Sport Med* 2007; **17**: 349–56.

40 Kirkley A, Griffin S, Whelan D. The development and validation of a quality of life-measurement tool for patients with meniscal pathology: the Western Ontario Meniscal Evaluation Tool (WOMET). *Clin J Sport Med* 2007; **17**: 349.

41 Wang, Dean1 Wang D, Jones MH, Khair MM MAP outcome measures for the knee. JKS 2010; 23: 137–51., Jones MH, Khair MM, Miniaci A. Patient-reported outcome measures for the knee. *J Knee Surg* 2010; **23**: 137–51.

42 Thorborg K, Tijssen M, Habets B, *et al.* Patient-Reported Outcome (PRO) questionnaires for young to middle-aged adults with hip and groin disability: a systematic review of the clinimetric evidence. *Br J Sports Med* 2015; **49**: 812.

43 Thoomes-de Graaf M, Scholten-Peeters GGM, Schellingerhout JM, *et al.* Evaluation of measurement properties of self-administered PROMs aimed at patients with non-specific shoulder pain and activity limitations: a systematic review. *Qual Life Res* 2016; **25**: 2141–60.

44 Green A, Liles C, Rushton A, Kyte DG. Measurement properties of patient-reported outcome measures (PROMS) in Patellofemoral Pain Syndrome: A systematic review. *Man Ther* 2014; **19**: 517–26.

45 Terwee CB, Bouwmeester W, van Elsland SL, de Vet HCW, Dekker J. Instruments to assess physical activity in patients with osteoarthritis of the hip or knee: A systematic review of measurement properties. *Osteoarthr Cartil* 2011; **19**: 620–33.

46 Gagnier JJ, Mullins M, Huang H, *et al.* A Systematic Review of Measurement Properties of Patient-Reported Outcome Measures Used in Patients Undergoing Total Knee Arthroplasty. *J Arthroplasty* 2017. DOI:10.1016/j.arth.2016.12.052.

47 Kroman SL, Roos EM, Bennell KL, Hinman RS, Dobson F. Measurement properties of performance-based outcome measures to assess physical function in young and middle-aged people known to be at high risk of

For peer review only

hip and/or knee osteoarthritis: A systematic review. *Osteoarthr Cartil* 2014; **22**: 26–39.

48 Dobson F, Hinman RS, Hall M, Terwee CB, Roos EM, Bennell KL. Measurement properties of performance-based measures to assess physical function in hip and knee osteoarthritis: A systematic review. *Osteoarthr Cartil* 2012; **20**: 1548–62.

49 Tijssen M, van Cingel R, van Melick N, de Visser E. Patient-Reported Outcome questionnaires for hip arthroscopy: a systematic review of the psychometric evidence. *BMC Musculoskelet Disord* 2011; **12**: 117.

50 van der Wal RJP, Heemskerk BTJ, van Arkel ERA, Mokkink LB, Thomassen BJW. Translation and Validation of the Dutch Western Ontario Meniscal Evaluation Tool. *J Knee Surg* 2016; **1**. DOI:10.1055/s-0036-1584576.

51 Mokkink LB, Terwee CB, Gibbons E, *et al.* Inter-rater agreement and reliability of the COSMIN (COnsensus-based Standards for the selection of health status Measurement Instruments) Checklist. *Med Res Methodol* 2010; **10**: 1–11.

52 McKenna SP. Measuring patient-reported outcomes: moving beyond misplaced common sense to hard science. *BMC Med* 2011; **9**: 86.

53 Tornbjerg SM, Nissen N, Englund M, *et al.* Structural pathology is not related to patient-reported pain and function in patients undergoing meniscal surgery. *Br J Sports Med* 2016; : bjsports-2016-096456.

54 Thorlund JB, Juhl CB, Roos EM, Lohmander LS. Arthroscopic surgery for degenerative knee: systematic review and meta-analysis of benefits and harms. *Bmj* 2015; **350**: h2747–h2747.

55 Thorlund JB, Englund M, Christensen R, *et al.* Patient reported outcomes in patients undergoing arthroscopic partial meniscectomy for traumatic or degenerative meniscal tears: comparative prospective cohort study. *Bmj* 2017; : j356.

56 Sihvonen R, Järvelä T, Aho H, Järvinen LN. Validation of the Western Ontario Meniscal Evaluation Tool (WOMET) for patients with a degenerative meniscal tear: a meniscal pathology-specific quality-of-life index. *J Bone Joint Surg Am* 2012; **94**.

57 Celik D, Demirel M, Kuş G, Erdil M, Özdinçler Razak A. Translation, cross-cultural adaptation, reliability and validity of the Turkish version of the Western Ontario Meniscal Evaluation Tool (WOMET). *Knee Surg Sports Traumatol Arthrosc* 2015; **23**: 816.

58 Tong WW, Wang W, Xu WD. Development of a Chinese version of the Western Ontario Meniscal Evaluation Tool: cross-cultural adaptation and psychometric evaluation. *J Orthop Surg Res* 2016; **11**: 90.

59 Bellamy N, Buchanan WW, Goldsmith CH, Campbell J, Stitt LW. Validation study of WOMAC: a health status instrument for measuring clinically important patient relevant outcomes to antirheumatic drug therapy in patients with osteoarthritis of the hip or knee. *J Rheumatol* 1988; **15**: 1833–40.

For peer review only

Figures

Figure 1: Overview of study selection

Full search strategy may be found in supplementary appendix 1.

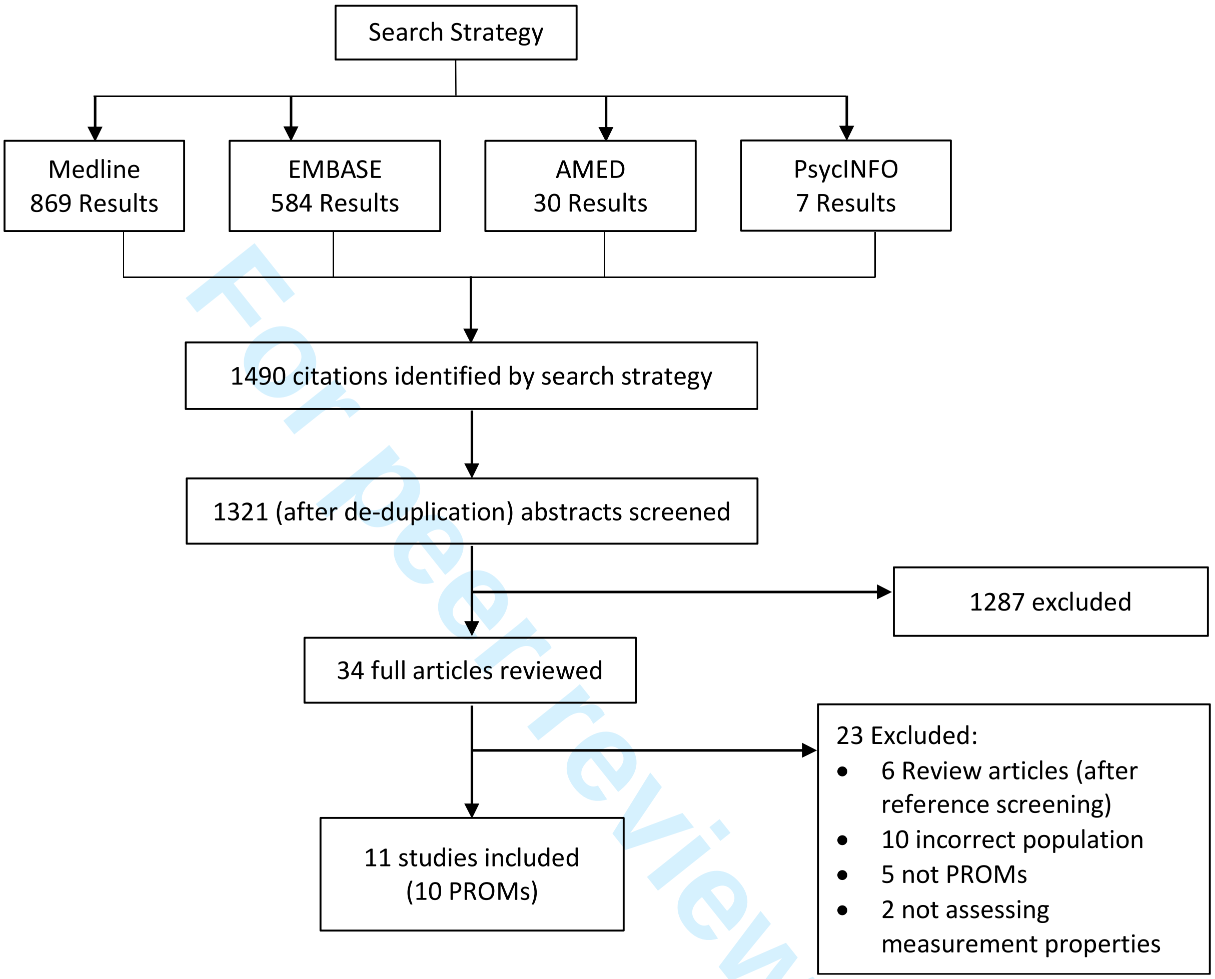

For peer review only

Tables

Table 1: Overall levels of evidence for the quality of the measurement property[22,23]

The quality of the evidence for the measurement property for each PROM, considering the quality criteria for each measurement property (Table 1), the methodology of each study reporting the measurement property (Table 5) and the number of studies reporting the measurement property including consistency of findings.

Level of Evidence	Rating	Quality Criteria
Strong	+++ or - - -	Consistent findings (positive or negative) in multiple studies of good methodological quality OR in one study of excellent methodological quality
Moderate	++ or - -	Consistent findings (positive or negative) in multiple studies of fair methodological quality OR in one study of good methodological quality
Limited	+ or -	One study of fair methodological quality (positive or negative)
Conflicting	+/-	Conflicting results
Unknown	?	Only studies of poor methodological quality

+ = positive rating, ? = indeterminate rating, - = negative rating

For peer review only

Table 2: Characteristics of the included studies

Study (year)	Instrument(s)	Country (language)	Population (inclusion and exclusion criteria)	*N*	Mean age (SD, range)	Female : Male
Goodwin (2011)[25]	**Hughston**<br>**EQ-5D**<br>**SF-6D**	**UK (English)**	**Inclusion:** Patients previously undergoing arthroscopic partial meniscectomy.	84	38 (SD 8, 21-58)	14 : 86 %
Crawford (2007)[27]	**IKDC**	**USA (English)**	**Inclusion:** Patient with "meniscal pathology requiring treatment" and completed IKDC questionnaire<br>**Exclusion:** Patients with ligament pathology or a chondral defect greater than Outerbridge grade 2	Groups:<br>A: 31<br>B: 264<br>C: 50<br>D: 50	48 (18-81)	29 : 71 %
Van de Graaf (2014)[28]	**IKDC**<br>**KOOS**<br>**WOMAC**	**Netherlands (Dutch)**	**Inclusion:** Age >18, knowledge of Dutch language, either on waiting list for meniscal surgery or between 6 weeks and 6 months after meniscal surgery.<br>**Exclusion:** Received arthroplasty in either knee or previous ACL surgery on the knee of interest.	75	48.8 (35-62)	50 : 50 %
Roos (1998)[31]	**KOOS**	**Sweden (Swedish)**	**Inclusion:** Patient waiting for knee arthroscopy for either meniscal lesion, ACL injury or tibio-femoral cartilage damage.<br>(54% meniscal tear, 20% ACL+meniscal tear, 13% ACL, 13% isolated chondral damage)<br>**Exclusion:** Multiple joint involvement, other diagnosis.	142	39.7 (14-75)	22 : 78 %
Garratt (2008)[37]	**KQoL-26**	**UK (English)**	**Inclusion:** Patients aged 18-55, referred to hospital clinic with suspected meniscus or knee ligament pathology. (67% meniscal tear, 30% ACL, 3% other)<br>**Exclusion:** Requiring urgent referral, non-traumatic arthropathy, chronic knee instability, previous same knee surgery (except diagnostic arthroscopy)	323	47 (14.3)	44 : 56 %
Briggs (2006)[34]	**Lysholm**<br>**Tegner**	**USA (English)**	**Inclusion:** Patient previously undergoing surgery for meniscal lesion or waiting list for meniscal surgery.	Groups:<br>A: 122<br>B: 191<br>C: 477	40 (13-81)	32 : 68 %
Kirkley (2007)[40]	**WOMET**	**Canada (English)**	**Inclusion:** Patients with "meniscal symptoms (swelling, catching, locking)" and magnetic resonance imaging suggestive of meniscal pathology.	Groups:<br>A: 31<br>B: 36<br>C: 34<br>D: 69	Not reported	Not reported
Sihvonen (2012)[56]	**WOMET**	**Finland (Finnish)**	**Inclusion:** Patients with arthroscopically verified degenerative meniscal tear and no previous knee trauma.<br>**Exclusion:** Trauma, bilateral arthroscopy, re-operation within 6 months	Groups:<br>A: 485<br>B: 385<br>C: 100<br>D: 40	53 (18-81)	45 : 55 %
Celik (2013)[57]	**WOMET**	**Turkey (Turkish)**	**Inclusion:** Age >16, presence of meniscal tear or previous meniscal repair or resection, complete questionnaires.<br>**Exclusion:** Ligament injury, "articular cartilage damage causing instability", inability to complete the form due to cognitive impairment.	96	43.6 (23-71)	64 : 36 %
Tong (2016)[58]	**WOMET**	**China (Chinese)**	**Inclusion:** Patients with meniscal pathology who underwent arthroscopic surgery for meniscal repair or resection. Age >18, able to read and speak Chinese.<br>**Exclusion:** Ligament injuries, history of leg surgery, infection, tumours, rheumatologic disease, neurological or musculoskeletal disorders.	121	41.2 (14.3)	57 : 43 %
Van der Wal (2016)[50]	**WOMET**	**Netherlands (Dutch)**	**Inclusion:** Patients with MRI confirmed, symptomatic, meniscal tear. Age 18-70, understanding of Dutch language.<br>**Exclusion:** Concomitant ligament injury, previous ligament injury with instability, previous knee surgery, chondral defect greater than Outerbridge grade 2 on MRI or during surgery, inability to participate due to cognitive impairment.	86	Median 52 (IQr 43-60)	41 : 59 %

IQr = interquartile range.

For peer review only

Table 3: Characteristics of the included PROMs

Instrument	Year of development	Original language	Intended Construct & Domains	Number of questions	Target or Development Population	Patients involved in development?
**Symptoms & Functional Status**						
Hughston	1991[24]	English	**Knee-specific symptoms, functional status, sports activity.**<br>No sub-domains	28 questions	"Patients who had undergone knee surgery that varied from arthroscopy to total arthroplasty."[24]	No
IKDC	2001[26]	English	**Knee-specific symptoms, functional status, sports activity.**<br>1. Symptoms<br>2. Sports activities<br>3. Function	18 questions	"A knee-specific, rather than a disease-specific, measure of symptoms, function, and sports activity."[26]	No
KOOS	1998[29]	English	**Knee injury-specific symptoms, functional status, sports activity, quality of life.**<br>1. Symptoms & Stiffness<br>2. Pain<br>3. Activities of daily living (ADL)<br>4. Function in sports and recreation<br>5. Knee-related quality of life (QoL)	42 questions	Patients with knee injury (ACL or meniscus injury) at risk of developing osteoarthritis.	Yes
Lysholm	1982[32] / 1985[33]	English	**Disease-specific (knee ligament) symptoms, functional status**<br>No sub-domains	8 questions	"A scoring scale for knee ligament surgery follow-up emphasizing evaluation of symptoms of instability."[32]	No
WOMAC	1982[35]	English	**Disease-specific (osteoarthritis of hip or knee) symptoms, functional status**<br>1. Pain<br>2. Stiffness<br>3. Function & Daily activities	24 questions	"Outcomes of anti-rheumatic drug therapy in patients with osteoarthritis of the hip or knee."[59]	Yes
**Health-related quality of life**						
EQ-5D	1990[36]	English, Dutch, Finnish, Norweigan, Swedish	**General population health-related quality of life**<br>1. Mobility<br>2. Self-care<br>3. Usual activities<br>4. Pain/Discomfort<br>5. Anxiety/Depression	6 questions	General tool for describing and valuing health related quality of life – items developed and valued after questioning large samples of randomly selected adults.	Yes
KQoL-26	2008[37]	English	**Disease-specific (knee ligament or meniscus) health-related quality of life**<br>1. Physical functioning<br>2. Activity limitations<br>3. Emotional functioning	26 questions	"Patients with a suspected ligamentous or meniscal injury of the knee."[37]	Yes
SF-6D	2004[38]	English	**General population health-related quality of life**<br>1. Physical functioning<br>2. Role limitation<br>3. Social functioning<br>4. Pain<br>5. Mental health<br>6. Vitality	6 questions	Derived from SF-36 or SF-12. A general, preference based classification for describing health-related quality of life.	Yes
WOMET	2007[39]	English	**Disease-specific (meniscus) health-related quality of life**<br>Physical symptoms<br>1. Sports/Recreation/Work/Lifestyle<br>2. Emotions	16 questions	"Patients with meniscal symptomology (swelling, catching, locking) and in whom magnetic resonance imaging had suggested meniscal pathology."[39]	Yes
**Activity level**						
Tegner	1985[33]	English	**Disease-specific (knee ligament) symptoms, functional status**<br>No sub-domains	1 question	Patients with ACL injury diagnosed by clinical examination under anaesthesia and confirmed by arthroscopy or arthrotomy.	No

ACL = anterior cruciate ligament

For peer review only

Table 4: Interpretability including missing items, response rate, floor and ceiling effects

Instrument and Study	Administration	Missing responses	Missing items	Overall % lowest possible total score (floor)	Overall % highest possible score (ceiling)	Items or Domains with >15% responses with lowest score (floor)	Items or Domains >15% highest possible score (ceiling)	MIC
**Symptoms & Functional Status**								
**Hughston**								
Goodwin (2011)[25]	Clinic	Not reported	Not reported	0%	0%	Not reported	Not reported	Not reported
**IKDC**								
Crawford (2007)[27]	Clinic / Postal	Not reported	Not reported	0%	0%	• Activity pain<br>• Pain last 4 weeks<br>• Pain severity<br>• Catching<br>• Kneeling<br>• Sitting<br>• Running<br>• Jumping<br>• Stopping	• Swelling<br>• Catching<br>• Climb stairs<br>• Sitting<br>• Rising	Not reported
Van de Graaf (2014)[28]	Online / Postal	Unclear	0%	0%	0%	Nil	Nil	Not reported
**KOOS**								
Roos (1998)[31]	Postal	7.2%	0.8%	Not reported	Not reported	Nil	Nil	Not reported
Van de Graaf (2014)[28]	Online / Postal	Unclear	0%	0%	3%	Nil	Nil	Not reported
**Lysholm**								
Briggs (2006)[34]	Clinic	Not reported	Not reported	0%	0.5%	• Squatting<br>• Pain	• Swelling<br>• Instability<br>• Support<br>• Limp<br>• Locking	Not reported
**WOMAC**								
Van de Graaf (2014)[28]	Online / Postal	Unclear	0%	0%	6%	Nil	Nil	Not reported
**Health-related quality of life**								
**EQ-5D**								
Goodwin (2011)[25]	Clinic	Not reported	Not reported	4%	1%	Not reported	Not reported	Not reported
**KQoL-26**								
Garratt (2008)[37]	Postal	41%	14.9%	Not reported	Not reported	• Avoiding turning, twisting, or sideways movements<br>• *	• Staying seated for 15 minutes<br>• *	Not reported
**SF-6D**								
Goodwin (2011)[25]	Clinic	Not reported	Not reported	0%	0%	Not reported	Not reported	Not reported
**WOMET**								
Kirkley (2007)[40]	Clinic	Not reported	Not reported	5.7%	1.7%	Not reported	Not reported	Not reported
Sihvonen (2012)[56]	Unclear	16%	7.5%	0%	0%	• Numbness	Nil	Not reported
Celik (2013)[57]	Unclear	Not reported	Not reported	0%	0%	• Numbness<br>• Swelling	• Consciousnes s<br>• Activities<br>• Specific skills<br>• Squatting<br>• Fear injury<br>• Concern about future of knee<br>• Frustration	Not reported
Tong (2016)[58]	Unclear	Not reported	0%	0%	0%	Nil	Nil	Not reported
Van der Wal (2016)[50]	Clinic	0%	<1%	0%	0%	• Numbness<br>• Swelling	Nil	14.7
**Activity level**								
**Tegner**								
Briggs (2006)[34]	Clinic	Not reported	Not reported	2.5%	2.5%	na	na	Not reported

MIC = minimal important change; * = Other domains not reported

For peer review only

Table 5: Methodology quality of each study per PROM and measurement property (COSMIN rating)

Instrument and Study	Internal consistency	Reliability	Measurement error	Content validity	Structural validity	Hypothesis testing	Cross-cultural validity	Responsiveness
Symptoms & Functional Status								
**Hughston**								
Goodwin (2011)[25]	Poor	na	na	Poor	na	Good	na	Poor
**IKDC**								
Crawford (2007)[27]	Poor	Fair	Fair	Poor	na	Fair	na	Poor
Van de Graaf (2014)[28]	Poor	Good	Good	Poor	Fair	Good	Poor	na
**KOOS**								
Roos (1998)[31]	Poor	Fair	na	Poor	Poor	Fair	Poor	Poor
Van de Graaf (2014)[28]	Poor	Good	Good	Poor	Poor	Good	Poor	na
**Lysholm**								
Briggs (2006)[34]	Poor	Fair	Fair	Poor	na	Fair	na	Poor
**WOMAC**								
Van de Graaf (2014)[28]	Poor	Good	Good	Poor	Poor	Good	Poor	na
Health-related quality of life								
**EQ-5D**								
Goodwin (2011)[25]	Poor	na	na	Poor	na	Good	na	Poor
**KQoL-26**								
Garratt (2008)[37]	Fair	Fair	na	Fair	Fair	Fair	na	Poor
**SF-6D**								
Goodwin (2011)[25]	Poor	na	na	Poor	na	Good	na	Poor
**WOMET**								
Kirkley (2007)[40]	Poor	Fair	na	Excellent	na	Fair	na	Fair
Sihvonen (2012)[56]	Poor	Poor	na	Poor	na	Fair	Poor	Poor
Celik (2013)[57]	Poor	Good	Good	Poor	na	Good	Poor	na
Tong (2016)[58]	Poor	Good	na	Poor	na	Good	Poor	Poor
Van der Wal (2016)[50]	Poor	Good	Good	Good	na	Good	Poor	Good
Activity level								
**Tegner**								
Briggs (2006)[34]	na	Fair	Fair	Poor	na	Fair	na	Poor

For peer review only

Table 6: Overall rating of measurement properties and level of evidence for each PROM

Instrument	Internal consistency	Reliability	Measurement error	Content validity	Structural validity	Hypothesis testing	Cross-cultural validity	Responsiveness
**Symptoms & Functional Status**								
**Hughston**								
English[25]	?	na	na	?	na	- -	na	?
**IKDC**								
English[27]	?	+	?	?	na	+	na	?
Dutch[28]	?	++	?	?	-	++	?	na
**KOOS**								
Dutch[28]	?	++	?	?	?	++	?	na
Swedish[31]	?	+	na	?	?	+	?	?
**Lysholm**								
English[34]	?	+	?	?	na	+	na	?
**WOMAC**								
Dutch[28]	?	++	?	?	?	++	?	na
**Health-related quality of life**								
**EQ-5D**								
English[25]	?	na	na	?	na	++	na	?
**KQoL-26**								
English[37]	+	+	na	+	+	+	na	?
**SF-6D**								
English[25]	?	na	na	?	na	++	na	?
**WOMET**								
English[40]	?	+	na	+++	na	+	na	+
Chinese[58]	?	++	na	?	na	++	?	?
Dutch[50]	?	++	- -	++	na	++	?	++
Finnish[56]	?	?	na	?	na	+	?	?
Turkish[57]	?	++	?	?	na	++	?	na
**Activity level**								
**Tegner**								
English[34]	na	+	?	?	na	+	na	?

For peer review only

Appendix 1: Search strategy

Databases: Medline, EMBASE, AMED, PsycInfo

#1 Condition

menis*.af

#2 Construct

("quality of life" OR qol OR func* OR HR-PRO OR HRPRO OR HRQOL OR QL OR disab* OR wellbeing OR "well being" OR subjective OR utility OR utilities OR priorit* OR outcome* OR health).af

#3 Instrument

(score* OR measure* OR PROM OR index* OR indices OR scale* OR questionnaire* OR instrument* OR survey* OR profile* OR apprais* OR status OR reported OR reporting OR rated OR rating* OR assessment*).af

#4 Measurement Properties

"Validation Studies".pt OR instrumentation.af OR ("observer variation" OR "psychometrics" OR "reproducibility of results" OR "discriminant analysis").mh OR (agreement OR precision OR imprecision OR "precise values" OR repeatab* OR ((replicab* OR repeated) AND (measure OR measures OR findings OR result OR results OR test OR tests))).af OR (reproducib* OR psychometr* OR clinimetr* OR clinometr* OR observer AND variation OR reliab* OR valid* OR coefficient OR "internal consistency" OR (cronbach* AND (alpha OR alphas)) OR "item correlation" OR "item correlations" OR "item selection" OR "item selections" OR "item reduction" OR "item reductions" OR test?retest OR (test AND retest) OR (reliab* AND (test OR retest)) OR stability OR interrater OR inter-rater OR intrarater OR intra-rater OR intertester OR inter-tester OR intratester OR intra-tester OR interobserver OR inter-observer OR intraobserver OR intra-observer OR intertechnician OR intertechnician OR intratechnician OR intra-technician OR interexaminer OR inter-examiner OR intraexaminer OR intra-examiner OR inter-assay OR inter-assay OR intraassay OR intra-assay OR interindividual OR inter-individual OR intraindividual OR intra-individual OR interparticipant OR inter-participant OR intraparticipant OR intra-participant OR kappa OR kappa?s OR "coefficient of variation" OR generaliza* OR generalisa* OR concordance OR (intraclass AND correlation*) OR discriminative OR "known group" OR "factor analysis" OR "factor analyses" OR "factor structure" OR "factor structures" OR dimensionality OR subscale* OR "multitrait scaling analysis" OR "multitrait scaling analyses" OR "item discriminant" OR "interscale correlation" OR "interscale correlations" OR ((error OR errors) AND (measure* OR correlat* OR evaluat* OR accuracy OR accurate OR precision OR mean)) OR "individual variability" OR "interval variability" OR "rate variability").ti,ab NOT ("addresses" OR "biography" OR "case reports" OR "comment" OR "directory" OR "editorial" OR "festschrift" OR "interview" OR "lectures" OR "legal cases" OR "legislation" OR "letter" OR "news" OR "newspaper article" OR "patient education handout" OR "popular works" OR "congresses" OR "consensus development conference" OR "consensus development conference, nih" OR "practice guideline").pt

#5 (#1 AND #2 AND #3 AND #4)

#5 Remove Duplicates

For peer review only

Appendix 2: Quality criteria for measurement properties[1]

Property	Rating	Quality Criteria
**Reliability**		
Internal consistency	+	Cronbach's alpha(s) ≥ 0.70
	?	Cronbach's alpha not determined or dimensionality unknown
	-	Cronbach's alpha(s) < 0.70
Reliability	+	ICC / weighted Kappa ≥ 0.70 OR Pearson's r ≥ 0.80
	?	Neither ICC / weighted Kappa, nor Pearson's r determined
	-	ICC / weighted Kappa < 0.70 OR Pearson's r < 0.80
Measurement error	+	MIC > SDC OR MIC outside the LOA
	?	MIC not defined
	-	MIC ≤ SDC OR MIC equals or inside LOA
**Validity**		
Content validity	+	All items are considered to be relevant for the construct to be measured, for the target population, and for the purpose of the measurement AND the questionnaire is considered to be comprehensive
	?	Not enough information available
	-	Not all items are considered to be relevant for the construct to be measured, for the target population, and for the purpose of the measurement OR the questionnaire is considered not to be comprehensive
Construct validity – Structural validity	+	Factors should explain at least 50% of the variance
	?	Explained variance not mentioned
	-	Factors explain < 50% of the variance
Construct validity – Hypothesis testing	+	Correlations with instruments measuring the same construct ≥ 0.50 OR at least 75% of the results are in accordance with the hypotheses AND correlations with related constructs are higher than with unrelated constructs
	?	Solely correlations determined with unrelated constructs
	-	Correlations with instruments measuring the same construct < 0.50 OR < 75% of the results are in accordance with the hypotheses OR correlations with related constructs are lower than with unrelated constructs
Construct validity – Cross-cultural validity	+	No differences in factor structure OR no important DIF between language versions
	?	Multiple group factor analysis not applied AND DIF not assessed
	-	Differences in factor structure OR important DIF between language versions
**Responsiveness**		
Responsiveness	+	Correlation with changes on instruments measuring the same construct ≥ 0.50 OR at least 75% of the results are in accordance with the hypotheses OR AUC ≥ 0.70 AND correlations with changes in related constructs are higher than with unrelated constructs
	?	Solely correlations determined with unrelated constructs
	-	Correlations with changes on instruments measuring the same construct < 0.50 OR < 75% of the results are in accordance with the hypotheses OR AUC < 0.70 OR correlations with changes in related constructs are lower than with unrelated constructs

MIC = minimal important change, SDC = smallest detectable change, LoA = limits of agreement, ICC = intraclass correlation coefficient, DIF = differential item functioning, AUC = area under the curve
+ = positive rating, ? = indeterminate rating, - = negative rating

1 Terwee CB, Bot SDM, de Boer MR, *et al.* Quality criteria were proposed for measurement properties of health status questionnaires. *J Clin Epidemiol* 2007; **60**: 34–42.

For peer review only

BMJ Open

Patient-reported outcome measures for patients with meniscal tears: a systematic review of measurement properties and evaluation with the COSMIN checklist

Journal:	*BMJ Open*
Manuscript ID	bmjopen-2017-017247.R1
Article Type:	Research
Date Submitted by the Author:	17-Jul-2017
Complete List of Authors:	Abram, Simon; University of Oxford Nuffield Department of Orthopaedics Rheumatology and Musculoskeletal Sciences,<br>Middleton, Robert; University of Oxford Nuffield Department of Orthopaedics Rheumatology and Musculoskeletal Sciences<br>Beard, David; Oxford University, Nuffield Dept of Orthopaedics, Rheumatology and Musculoskeletal Sciences<br>Price, Andrew; University of Oxford, Nuffield Department of Orthopaedics, Rheumatology and Musculoskeletal Sciences<br>Hopewell, Sally; University of Oxford, Oxford Clinical Trials Research Unit
**Primary Subject Heading**:	Surgery
Secondary Subject Heading:	Sports and exercise medicine, Patient-centred medicine
Keywords:	Knee < ORTHOPAEDIC & TRAUMA SURGERY, ORTHOPAEDIC & TRAUMA SURGERY, Orthopaedic & trauma surgery < SURGERY

SCHOLARONE™ Manuscripts

For peer review only

Patient-reported outcome measures for patients with meniscal tears: a systematic review of measurement properties and evaluation with the COSMIN checklist

S.G.F. Abram, R. Middleton, D.J. Beard, A.J. Price, S. Hopewell.

Authors' affiliation:

Nuffield Department of Orthopaedics, Rheumatology and Musculoskeletal Sciences, University of Oxford

Corresponding author:

Simon Abram

simon.abram@doctors.org.uk

Keywords:

outcomes; patient reported outcomes; psychometrics; meniscal pathology; meniscus; knee surgery; knee

For peer review only

Abstract

Objective: Meniscal tears occur frequently in the population and the most common surgical treatment, arthroscopic partial meniscectomy, is performed in approximately 2 million cases worldwide each year. The purpose of this systematic review is to summarise and critically appraise the evidence for the use of patient-reported outcome measures (PROMs) in patients with meniscal tears.

Design: A systematic review was undertaken. Data on reported measurement properties was extracted and the quality of the studies appraised according to Consensus-based Standards for the selection of health Measurement Instruments (COSMIN).

Data sources: A search of MEDLINE, Embase, AMED and PsycINFO, unlimited by language or publication date (last search 20/02/2017).

Eligibility criteria for selecting studies: Development and validation studies reporting the measurement properties of PROMs in patients with meniscal tears were included.

Results: 11 studies and 10 PROMs were included. The overall quality of studies was poor. For measurement of symptoms and functional status there is only very limited evidence supporting the selection of either the Lysholm knee scale, International Knee Documentation Committee (IKDC) Subjective Knee Form, or the Dutch-version of the Knee injury and Osteoarthritis Outcome Score (KOOS). For measuring health-related quality of life, only limited evidence supports the selection of the Western Ontario Meniscal Evaluation Tool (WOMET). Of all the PROMs evaluated, WOMET has the strongest evidence for content validity.

Conclusion: For patients with meniscal tears, there is poor quality and incomplete evidence regarding the validity of the currently available PROMs. Further research is required to ensure these PROMs truly reflect the symptoms, function, and quality of life of patients with meniscal tears.

PROPERO registration number: CRD42017056847

For peer review only

Strengths and limitations of this study

- This is the first review of PROMs for patients with meniscal tears and the first to apply the COSMIN checklist, which is a validated and accepted tool for the appraisal of study quality.
- Anotherstrength of this review is the use of a validated, highly-sensitive search strategy to identify relevant studies. A limitation, however, is that only studies specifically designed to appraise the measurement properties of PROMs were included. Trials and other clinical studies of patients with meniscal tears were not included as these studies are not designed to assess measurement properties and the reporting of these properties would be highly unusual.
- Although the COSMIN checklist has acceptable inter-rater and intra-rater properties, the scoring of some items is reliant on author judgement. We performed pre-testing to ensure scoring consistency, and review authors scored studies independently. Nevertheless, it is feasible that another review team might score some items differently.
- For practical purposes, we chose to include tentative summary guidance regarding the selection of PROMs for use in the target population. It should be understood, however, that it would be reasonable to declare that the overall level of evidence for any of the PROMs is insufficient for a recommendation to be made.

For peer review only

Introduction

The menisci are fibrocartilaginous structures within the knee joint which are important for load distribution and knee stability.[1,2] More than one third of people over the age of 50 without any radiographic evidence of osteoarthritis may develop a 'tear' of the meniscus and over 70% of those with osteoarthritis will also have a torn meniscus.[3] These meniscal tears may be associated with significant knee pain and other symptoms, especially if the torn meniscal tissue interferes with the normal articulation of the joint.[4] Meniscal tears are diagnosed and managed based upon a combination of a review of symptoms, clinical examination, and imaging findings on x-ray radiographs and magnetic resonance imaging (MRI).[5] Arthroscopic partial meniscectomy is a surgical procedure commonly used to treat symptomatic meniscal tears with approximately two million cases performed worldwide each year with combined costs of several billion US dollars.[6] A number of recent randomised controlled trials have been published challenging the effectiveness of arthroscopic partial meniscectomy.[7–11] Patient-reported outcome measures (PROMs) are critical to the interpretation of these trials, yet a wide array of different PROMs have been collected. This inconsistency leads to restricted comparisons between trials and difficult interpretation of their findings.[12,13] The best PROM for this population is unknown.

Patient reported outcome measures (PROMs) are collected in a range of settings and are increasingly important in clinical practice. In orthopaedics, PROMs are important for auditing treatment outcomes and increasingly to demonstrate the cost-effectiveness of treatment.[14] With the rapid increase in usage, it is important to ensure that PROMs have formally validated measurement properties. Although generic PROMs enable the comparison of patients with different conditions, these PROMs may fail to capture important items in specific populations.[15] Ideally, a PROM should either be developed with condition-specific patient involvement or subsequently studied for validity in the population of interest.[16] Fundamentally, a PROM should comprehensively and consistently reflect the intended 'construct' to be measured in the population with the condition of interest – for example, health-related quality of life in patients with meniscal tears.[17]

There is a need for the selection of standardised 'core' PROMs for consistent use in clinical trials and the general clinical evaluation of patients with specific conditions.[13] A systematic review of the evidence is an important step in the selection of such a 'core outcome set' and may determine the need for further validation studies or even the development of a new PROM.[13] No systematic review has been published evaluating the measurement properties with the quality of evidence for the PROMs that are available for patients with meniscal tears. This is a barrier to the interpretation of previous research and to the design of future studies in these patients.

The purpose of this review is to report the measurement properties and evidence for the validity of all PROMs which have been evaluated in patients with meniscal tears.

For peer review only

Methods

This systemic review is reported based upon the Preferred Reporting Items for Systematic Review and Meta-Analyses (PRISMA) guidelines.[18,19] The protocol for this review was submitted to PROSPERO (CRD42017056847) on 20/02/2017.

Study selection criteria

We included studies of adults with meniscal tears of the knee. Those studies with less than 50% of patients having a meniscal tear as the primary diagnosis (i.e. without other significant knee pathology e.g. concomitant anterior cruciate ligament (ACL) rupture) were excluded unless the meniscal tear group was reported separately. Studies administrating PROMs for the purpose of assessing measurement properties were included. PROMs using standard scoring methods, without clinician completed elements, measuring health related quality of life, health status, symptoms including pain, or functional status were included. Some studies included patients undergoing surgery (e.g. arthroscopic partial meniscectomy, meniscal repair) as part of this assessment process, but the purpose of this review was not to assess the effectiveness of such interventions.

Measurement properties

All PROM measurement properties reported by the included studies were evaluated. The primary measurement properties assessed were those within the reliability, validity, and responsiveness domains. The secondary domains assessed were interpretability and generalisability. The Consensus-based Standards for the selection of health Measurement Instruments (COSMIN) definition of the domains and measurement properties follows below.[20]

Reliability

The reliability domain is a measure of how free a PROM is from measurement error.[20] The measurement properties within this domain are assessed by repeated collection of the PROM in a defined period when there has been no change in the patient's condition. Ideally, rather than assume the patient's condition is unchanged, a methodologically strong study will assess for change, for example by administering a knee-specific global transition question on symptoms.

- Internal consistency: is the degree of inter-relatedness among the PROM items.[20]
- Reliability: is the proportion of total variance in the measurement which is because of true differences among patients.[20]
- Measurement error: is the systematic and random error of a patient's score that is not attributed to true changes in the construct to be measured.[20]

Validity

For peer review only

The validity domain is the extent to which the PROM measures the 'construct' it purports to measure.[20]

- Content validity: is the degree to which the content of a PROM is an adequate reflection of the construct to be measured.[20] The items should be comprehensive and relevant.
- Construct validity: is the degree to which the scores of a PROM are consistent with hypotheses (e.g. relationship of the score to that of other PROMs collected in the same group) based on the assumption the PROM validly measures the intended construct.[20]
    - Structural validity: is the degree to which the scores of a PROM are an adequate reflection of the dimensionality of the construct to be measured.[20]
    - Hypothesis testing: assumes that the PROM validly measures the construct of interest. Hypotheses are prepared *a priori* with regards to the correlation of the PROM with other relevant PROMs or domains of other PROMs. The magnitude and direction of the correlation should be stated in advance of testing.
    - Cross-cultural validity: is the degree to which the performance of the items on a translated or culturally adapted PROM are comparable to the performance of the original version of the PROM.[20]

Responsiveness

Responsiveness is defined as the ability of a PROM to measure change over time in the construct to be measured.[20] It is important to note that in studies assessing the measurement properties of a PROM, responsiveness should be assessed against another valid PROM as for the assessment of construct validity. Measurement of effect size alone is not appropriate as this is a measure of the magnitude of the change and not the quality of the measurement.[21]

Interpretability

Interpretability is defined as the degree to which it is possible to assign qualitative meaning to a PROM's quantitative score.[20] It is not considered a measurement property but is important when interpreting the findings from administration of a PROM in the context of a clinical condition. Interpretability includes an assessment of minimal important change (MIC), floor and ceiling effects. In general, floor and ceiling effects <15% are considered acceptable although some authors have argued the threshold should be set at <30%.[22,23] A high floor or ceiling effect suggests that items at the lower or upper end are missing from a question item, domain, or the PROM overall.

Generalisability

Generalisability is an assessment of external validity: the extent to which the findings on the measurement properties of a PROM may be considered relevant to a population or construct or interest. For example, a study of the measurement properties of a PROM in a population with advanced knee osteoarthritis cannot be generalised to athletes with knee ligament injury without further study in the target population. In this review, the population of

For peer review only

patients involved in the original development of each PROM is determined and the inclusion and exclusion criteria of all studies reporting measurement properties of the included PROMs is reported to highlight any heterogeneity. The generalisability of findings to the population of patients with meniscal tears is considered.

Search Strategy

We performed a search of MEDLINE, Embase, AMED and PsycINFO, unlimited by language or publication date. The search was based upon a validated search filter designed to be highly sensitive in identifying all studies of measurement properties.[24] Full details of the search are available in supplementary appendix 1. The final search was performed on 20/02/2017, following submission of the protocol to PROSPERO. A review of study citations was performed to further increase the sensitivity of the search strategy.

Selection of studies

The title and abstract of all records retrieved by the search was independently reviewed by two authors against the inclusion and exclusion criteria (SA and RM). Any disagreement was resolved with review of the full text publication and discussion. Referral to a third author (SH) was not required for agreement. The original PROM development article was retrieved for all PROMs identified – for example, where a PROM was developed for a condition other than meniscal pathology and subsequently tested in a population with meniscal tears.

Data extraction: Measurement properties and assessing the quality of studies

Data extraction was performed by two authors (SA and RM) and any disagreement resolved in consultation with a third author (SH). The following was extracted from each publication: the PROM, the intended construct for measurement, measurement properties, administration method, study population and diagnosis, number of patients, patient demographics, country, language and setting and method of administration (e.g. postal, online).

The quality of each included study was assessed by two reviewers (SA and RM) using the Consensus-based Standards for the selection of health Measurement Instruments (COSMIN) appraisal checklist.[25] When reviewing a study of a PROM, it is necessary to consider a combination of the reported measurement properties, the patient population, and the quality of the study methodology. To help overcome some of the difficulties in evaluating the quality of PROMs, COSMIN was published in 2010.[21,26] COSMIN contains rules for grading overall methodological quality of studies performed into the measurement properties of PROMs. These consensus standards are regularly reviewed and revised based on the latest evidence and research. COSMIN initially separated standards into boxes including a series of binary methodological ratings. The scoring methodology was subsequently revised to a four level (excellent/good/fair/poor) rating system in 2012.[25] Each measurement property is assessed by a box containing 5-18 questions scored on this scale according to defined COSMIN criteria. A system of 'worst score counts' applies for

For peer review only

each box – that is, if one question in the box is scored as poor, the overall quality of the evidence for that measurement property is determined to be poor.

Data synthesis

Data synthesis was performed by SA and checked by RM. For each included PROM, a summary of the features of the PROM is presented including details of the original development process, the development population and target construct to be measured.

For each PROM, a rating (positive, negative or indeterminate) for the measurement properties reported in the study was first determined based upon consensus standards described in supplementary appendix 2.[23] This assessment was then combined with an overall quality of evidence assessment which was adapted for COSMIN from the work of the Cochrane Back Review Group (Table 1).[27,28] For example, one good quality study reporting positive measurement properties (e.g. internal consistent with Cronbach's alpha ≥ 0.70) results in an overall rating of 'moderate' (++). Where the quality of study methodology on a measurement property is rated 'poor' the overall rating of the measurement property is always rated 'indeterminate', irrespective of the number of such studies and whether the reported measurement property itself would otherwise be considered positively. These standards are designed to ensure reported measurement properties are interpreted in the context of study quality and overall reliability.

For peer review only

Results

Selection of studies

The search strategy identified 1321 unique articles for screening. After screening, 34 full text articles were retrieved of which 11 met the inclusion criteria for this review. Figure 1 summarises the study selection process. The 11 studies reported measurement properties for 10 PROMs.

Study characteristics

The characteristics of the included studies are shown in Table 2. The mean age of patients included in the studies ranged from 38-53 years. The proportion of female patients included ranged from 14-64%. Inclusion and exclusion criteria were inconsistent with regards to age, symptoms, investigations and treatment (Table 2). The development and features of the included PROMs are summarised below and in Table 3.

Quality of the included studies

In total, the 11 studies reported 93 measurement properties for the 10 PROMs. The COSMIN methodology rating for 49 of these (53%) was poor. Many measurement properties were not reported and there was inconsistent reporting between studies (Table 4).

Quality of PROMs

Interpretability factors including floor and ceiling effects are summarised in Table 5. The overall level of evidence for the measurement properties of each PROM is summarised in Table 6. This combines the rating of the reported measurement property using the consensus criteria available in supplementary appendix 2 with the COSMIN scoring and the number of studies per PROM (as described in Table 1).

Of the 10 PROMs identified, five intended to measure symptoms and functional status, four health-related quality of life and one activity level.

Symptoms & Functional Status

Hughston: The Hughston Clinic Questionnaire was developed in 1991 as a knee-specific rather than disease-specific outcome measure.[29] It includes questions on symptoms, functional status and sports activity and patients were not involved in the development of the questions. Only one study has evaluated use of the Hughston questionnaire in patients with meniscal tears.[30] Content validity was rated poor as patients were not involved in the original development and content validity has not been subsequently assessed in patients with meniscal tears. In patients

For peer review only

with meniscal tears, there was moderate negative evidence against construct validity based on hypothesis testing and all other measurement properties were either not reported or indeterminate due to poor study design or reporting (Table 6).

IKDC: The International Knee Documentation Committee (IKDC) Subjective Knee Form was developed in 2001 as a knee-specific rather than disease-specific outcome measure.[31] It includes question domains on symptoms, functional status and sports activity and patients were not involved in the development of the questions. Two studies have evaluated use of the IKDC score in patients with meniscal tears.[32,33] In English, there is limited positive evidence for reliability and construct validity based on hypothesis testing.[32] In Dutch, there is moderate positive evidence for reliability and construct validity based on hypothesis testing but limited negative evidence against structural validity.[33] In both studies, all other measurement properties were either not reported or indeterminate (Table 6). For the English-version, although no floor or ceiling effect was detected for the overall score, unacceptable floor effects were reported for 9 items and unacceptable ceiling effects in 5 items (Table 5).

KOOS: The Knee injury and Outcome Osteoarthritis Score (KOOS) was developed in 1998 as a knee-injury specific outcome measure for patients at risk of developing osteoarthritis.[34] It includes question domains on symptoms, functional status, sports activity and quality of life. Patients with anterior cruciate ligament (ACL) or meniscal injuries were included in the development process. The KOOS includes the WOMAC osteoarthritis score in full and the WOMAC may therefore be calculated from the KOOS. The KOOS has been studied in Dutch and Swedish for patients with meniscal tears; no study has evaluated the English-version of KOOS in this population.[33,35] There is moderate positive evidence for reliability and construct validity from hypothesis testing of the Dutch-version.[33] For the Swedish-version, there is limited positive evidence for reliability and construct validity based on hypothesis testing.[36] For both the Dutch and Swedish versions, content validity and all other measurement properties were either rated indeterminate or were not reported (Table 6).

Lysholm: The Lysholm knee score was developed in 1982 and modified in 1985 as a disease-specific outcome measure for patients with knee ligament injury.[37,38] The Lysholm knee score was originally designed to be completed by clinicians and developed without patient involvement. One study has evaluated the use of Lysholm in English speaking patients with meniscal tears.[22] There is limited positive evidence for reliability and construct validity based on hypothesis testing. Content validity and all other measurement properties are either indeterminate or were not reported (Table 6). There was no floor or ceiling effect for the Lysholm score overall however an unacceptable floor effect was detected for 2 items and unacceptable ceiling effects for 5 items (Table 5).

WOMAC: The Western Ontario McMaster Osteoarthritis Index (WOMAC) was developed in 1982 as a disease-specific outcome measure for patients with osteoarthritis of the hip or knee.[39] The WOMAC includes question domains for pain, stiffness and functional status and patients with osteoarthritis were involved in the development of the questions. The WOMAC is incorporated in its entirety in the KOOS (see above). One study has evaluated the Dutch version of WOMAC in patients with meniscal tears.[33] In these patients, there is moderate positive evidence for

For peer review only

reliability and construct validity (hypothesis testing). No floor or ceiling effects were detected. Content validity and all other measurement properties are either indeterminate or were not reported (Table 6).

Health-related quality of life

EQ-5D: EQ-5D is a generic measure of health-related quality of life developed in 1990.[40] It was developed with patient involvement and includes question domains on mobility, self care, usual activities, pain, and anxiety or depression. One study has evaluated the English EQ-5D in patients with meniscal tears.[30] In this population, there is moderate positive evidence for construct validity based on hypothesis testing. All other measurement properties are either indeterminate or were not reported.

KQoL-26: The Knee Quality of Life (KQoL-26) 26-item questionnaire was developed in 2008, in English, as a disease-specific health-related quality of life measure for patients with suspected ligamentous or meniscal injury of the knee.[41] In the study population, 67% of patients had a meniscal tear and there is limited positive evidence for internal consistency, reliability, content validity, and construct validity (hypothesis testing and structural validity). Administered by post, an overall response rate of 59% was reported with 14.9% missing items.[41] Floor and ceiling effects were poorly reported with at least one question having an unacceptable floor effect and one an unacceptable ceiling effect (Table 5).

SF-6D: The short form-6 dimensions (SF-6D) generic health-related quality of life measure is derived from the SF-36 or SF-12 and was developed in 2004.[42] It was developed with patient involvement and contains 6 questions domains: physical functioning, role limitation, social functioning, pain, mental health and vitality. One study has evaluated the English version of SF-6D in patients with meniscal tears.[30] There is moderate positive evidence for construct validity based on hypothesis testing but all other measurement properties are indeterminate or were not reported.

WOMET: The Western Ontario Meniscal Evaluation Tool (WOMET) is a meniscal tear disease-specific, quality of life measure developed in 2007.[43] Patients with meniscal tears were involved throughout the development process although the authors reported that the same patients were “admittedly heterogeneous with respect to the incidence of coexisting knee pathology such as chondral damage or ligament injury”.[43] The WOMET has been evaluated in English, Chinese, Dutch, Finnish and Turkish. There is strong positive evidence for content validity in the English version and moderate positive evidence in the Dutch version. There is limited positive evidence for reliability, construct validity (hypothesis testing) and responsiveness of the English-version.[43] Measurement error was only reported for the Dutch-version of WOMET and in this case it was concerning that the minimal important change (MIC) for the PROM was found to be less than the smallest detectable change (SDC). A summary of the level of evidence for the measurement properties in all languages is shown in Table 6. Although the overall score does not exhibit floor or ceiling effects, unacceptable levels were reported for several items (Table 5).

For peer review only

Activity Level

Tegner: The Tegner Activity Scale was developed in 1985 for patients with ACL injury.[38] Patients were not involved in the development of the scale. One study has evaluated use of the scale in patients with meniscal tears.[22] In this population, there is limited positive evidence for reliability and construct validity based on hypothesis testing. All other measurement properties were either not reported or indeterminate.

For peer review only

Discussion

This review identified 11 studies evaluating 10 PROMs in patients with meniscal tears: five PROMS measuring symptoms and functional status, four PROMs measuring health-related quality of life and one for activity level. Unfortunately, the findings of the studies were limited by poor methodology and incomplete reporting of PROM measurement properties.

One previous review has been published summarising reported measurement properties of a range of PROMs in studies of patients with any knee condition.[44] In this previous review, WOMET was broadly recommended for use in patients with meniscal injuries without distinguishing the intended health-related quality of life construct from others or assessing the quality of the studies.[44] Ours is the first systematic review of PROMs for patients with meniscal tears and the first to evaluate and report the quality of study methodology. In orthopaedics and sports medicine, systematic reviews of PROMs applying the COSMIN appraisal checklist are established and have been published for patient populations including those with hip and knee osteoarthritis, hip and groin disability, patellofemoral pain, distal radius fractures, shoulder pain, and undergoing hip arthroscopy.[45–52]

For studies included in this review, the COSMIN methodology rating was poor for just over half (53%) of reported measurement properties. Internal consistency was rated poor in all but one of the 11 studies. A key reason for this was the failure of most studies to perform factor analysis to assess the structural validity of PROMs. Internal consistency is an assessment of the inter-relatedness of the items measuring the same underlying construct i.e. the PROM or sub-domain should be 'unidimensional' for the construct to be measured. Factor analysis is a technique that may be used determine whether a PROM or sub-domain is 'unidimensional'. Without this assessment of structural validity, there can be no clear interpretation of internal consistency statistics.[21]

Cross-cultural validity and responsiveness were also particularly poorly evaluated. Regarding responsiveness, frequently studies reported only an effect size for the studied PROM. Effect size alone is measure of the magnitude of a change scores and not the quality of the measurement and is therefore insufficient to assess this measurement property.[21] Responsiveness refers to the validity of a change score and should be assessed with, for example, hypothesis testing against the change score of another related PROM, analogous to the assessment of construct validity.

Measurement error was poorly reported in the included studies and the minimal important change (MIC) was calculated for only one of the PROMS – the Dutch-version of WOMET.[53] It was concerning that in this case the MIC was found to be less than the smallest detectable change (SDC) due to measurement error. Failure to determine and report this information affects the ability of researchers to design high-quality prospective studies and limits interpretation of previous work.

For peer review only

Evidence for the content validity of the available PROMs was limited. Only the KQoL-26 and WOMET were developed with involvement from patients with meniscal tears. Overall, there was heterogeneity in the population of the patients recruited to the included studies as shown in Table 2. Although most patients in the included studies had meniscal tears as their primary diagnosis, many also had a diagnosis of ligament injury or chondral damage. This reflects the heterogeneity of patients with meniscal tears in general, ranging from the isolated traumatic tear in a young athlete without osteoarthritis to atraumatic tears in older patients with osteoarthritis. Meniscal tears are not always symptomatic, and given the association with osteoarthritis, the distinction between the onset of meniscal pain and osteoarthritic pain is often unclear.[3,54] No single patient factor is sufficient in isolation.[5] For example, the degenerative meniscus will be more susceptible to tearing following knee trauma than a normal meniscus and no difference in symptom profile or treatment response has been demonstrated based on the mechanism of symptom onset.[55,56]

For studies in this review, there was significant variation in the methods used to identify patients. The latest guidance states specific types of meniscal tears should be identified on MRI imaging and related to symptoms and other findings before any surgical intervention is recommended.[5] Several studies included only patients with meniscal tears verified by previous arthroscopic surgery whereas others verified meniscal tears were visible on MRI imaging. For all the included studies, it was unclear how patients were identified to have symptoms correlating with a meniscal tear rather than other pathology such as osteoarthritis. Identifying patients with symptoms that definitely originate from the meniscus is challenging for both clinicians and researchers.

The mean age range of patients included in the studies was 38-53 years and therefore the generalisability of the findings to other age groups is unclear. It is highly likely that the symptom profile and expectations of younger, active patients sustaining a tear to a normal meniscus in an otherwise normal knee will be different to the study patients with predominantly degenerative meniscal tears and underlying osteoarthritis. This has not yet been evaluated.

Strengths and limitations

One strength of this review is the use of a validated, highly-sensitive search strategy to identify relevant studies.[24] A limitation, however, is that only studies specifically designed to appraise the measurement properties of PROMs were included. Trials and other clinical studies of patients with meniscal tears were not included as these studies are not designed to assess measurement properties and the reporting of these properties would be highly unusual. For the same reason, clinical trial registries were not searched for ongoing studies.

This is the first review of PROMs for patients with meniscal tears and the first to apply the COSMIN checklist, which is a validated and accepted tool for the appraisal of study quality. Although it has been shown to have acceptable inter-rater and intra-rater properties, the scoring of some items is reliant on author judgement.[57] We performed pre-testing to ensure scoring consistency and review authors scored studies independently with any disagreement being

For peer review only - http://bmjopen.bmj.com/site/about/guidelines.xhtml

settled by consensus or discussion with a third author. Nevertheless, it is feasible that another review team might score some items differently.

For practical purposes, we chose to include tentative summary guidance regarding the selection of PROMs for use in the target population. It should be understood, however, that it would be reasonable to declare that the overall level of evidence for any of the PROMs is insufficient for a recommendation to be made. Due to the study population limitations discussed earlier, the generalisability of the summary findings may also be challenged.

Implications for practice

Currently, although a wide range of PROMs are available for patients with knee conditions, the PROMs that have been tested in patients with meniscal tears all lack data on a large proportion of measurement properties. This is disappointing given moves to select condition-specific, standardised, 'core' outcome sets for use in clinical trials and general clinical evaluation.[13] Considerable further work is required before this will be possible for patients with meniscal tears.

For the assessment of symptoms and functional status in patients with meniscal tears, there is currently only very limited evidence supporting the selection of the English-version of Lysholm or IKDC, or Dutch-version of KOOS. Although the total score of these three PROMs does not exhibit floor or ceiling effects, a considerable number of sub-domain items from both IKDC and Lysholm were reported to have unacceptable floor or ceiling effects. For health-related quality of life, only limited evidence supports the selection of WOMET. One study suggests that measurement error may limit the ability of the WOMET to detect the minimal important change in score for meniscal patients.[53] Several WOMET sub-domain items, but not the total score, have been reported to exhibit unacceptable floor or ceiling effects. For assessment of activity level, only the Tegner activity scale has been evaluated and only very limited evidence is available.

Of all the PROMs evaluated, WOMET has the strongest evidence for content validity. In common with many of the validation studies in this population, however, the included patients frequently had other diagnoses in the same knee such as ligament injuries or chondral defects. This impacts upon the interpretation of clinical evidence in sub-groups of patients that were poorly represented within the development or validation study population. The findings of these validation studies may not be generalisable to such sub-groups and a PROM may fail to detect important clinical differences. Further validation studies may be required in sub-groups or the development of a more specific outcome measure may be necessary.[58] This is pertinent, for example, to current debate about the effectiveness of arthroscopic partial meniscectomy where there is an increasing focus on certain sub-groups of patients within this highly heterogeneous population.[12,56,59,60]

For peer review only

Conclusion

In summary, many PROMs have been used in clinical studies of patients with meniscal tears but the overall quality of evidence supporting the validity of these PROMs is poor. Further work is required, targeting the deficiencies highlighted by this systematic review, to ensure these PROMs truly reflect the symptoms, function, and quality of life of patients with meniscal tears. This is necessary to inform the design and interpretation of clinical studies of interventions such as arthroscopic partial meniscectomy in patients with meniscal tears.

For peer review only

Statements

Contributorship statement

S. Abram: methodology, study selection, analysis, writing and editing paper.
R. Middleton: study selection, analysis, editing paper.
D.J. Beard: concept, editing paper.
A. J. Price: concept, editing paper.
S. Hopewell: methodology, analysis, editing paper.

Competing interests

No competing interests.

Funding

This research received no specific grant from any funding agency in the public, commercial or not-for-profit sectors.

Data sharing statement

No additional data available.

For peer review only

References

1 Stephen JM, Halewood C, Kittl C, Bollen SR, Williams A, Amis AA. Posteromedial Meniscocapsular Lesions Increase Tibiofemoral Joint Laxity With Anterior Cruciate Ligament Deficiency, and Their Repair Reduces Laxity. *Am J Sports Med* 2016; **44**: 400–8.

2 Rao AJ, Erickson BJ, Cvetanovich GL, Yanke AB, Bach BR, Cole BJ. The Meniscus-Deficient Knee: Biomechanics, Evaluation, and Treatment Options. *Orthop J Sport Med* 2015; **3**: 2325967115611386.

3 Englund M, Guermazi A, Gale D, *et al.* Incidental meniscal findings on knee MRI in middle-aged and elderly persons. *N Engl J Med* 2008; **359**: 1108–15.

4 Roos EM, Roos HP, Ryd L, Lohmander LS. Substantial disability 3 months after arthroscopic partial meniscectomy: A prospective study of patient-relevant outcomes. *Arthroscopy* 2000; **16**: 619–26.

5 Stone JA, Salzler MJ, Parker DA, Becker R, Harner CD. Degenerative meniscus tears - assimilation of evidence and consensus statements across three continents: state of the art. *J ISAKOS Jt Disord Orthop Sport Med* 2017; **2**: 108–19.

6 Järvinen TLN, Guyatt GH. Arthroscopic surgery for knee pain. *Bmj* 2016; **3934**: i3934.

7 Sihvonen R, Paavola M, Malmivaara A, *et al.* Arthroscopic partial meniscectomy versus sham surgery for a degenerative meniscal tear. *N Engl J Med* 2013; **369**: 2515–24.

8 Katz JN, Brophy RH, Chaisson CE, *et al.* Surgery versus physical therapy for a meniscal tear and osteoarthritis. *N Engl J Med* 2013; **368**: 1675–84.

9 Herrlin S V., Wange PO, Lapidus G, Hållander M, Werner S, Weidenhielm L. Is arthroscopic surgery beneficial in treating non-traumatic, degenerative medial meniscal tears? A five year follow-up. *Knee Surgery, Sport Traumatol Arthrosc* 2013; **21**: 358–64.

10 Yim J-H, Seon J-K, Song E-K, *et al.* A comparative study of meniscectomy and nonoperative treatment for degenerative horizontal tears of the medial meniscus. *Am J Sports Med* 2013; **41**: 1565–70.

11 Kise NJ, Risberg MA, Stensrud S, Ranstam J, Engebretsen L, Roos EM. Exercise therapy versus arthroscopic partial meniscectomy for degenerative meniscal tear in middle aged patients: randomised controlled trial with two year follow-up. *BMJ* 2016; **354**. DOI:10.1136/bmj.i3740.

12 Monk P, Garfjeld Roberts P, Palmer AJR, *et al.* The Urgent Need for Evidence in Arthroscopic Meniscal Surgery: A Systematic Review of the Evidence for Operative Management of Meniscal Tears. *Am J Sports Med* 2016. DOI:10.1177/0363546516650180.

13 Williamson PR, Altman DG, Blazeby JM, *et al.* Developing core outcome sets for clinical trials: issues to consider. *Trials* 2012; **13**: 132.

14 van de Graaf VA, Scholtes VAB, Wolterbeek N, *et al.* Cost-effectiveness of Early Surgery versus Conservative Treatment with Optional Delayed Meniscectomy for Patients over 45 years with non-obstructive meniscal tears (ESCAPE study): protocol of a randomised controlled trial. *BMJ Open* 2016; **6**: e014381.

15 Brazier JE, Harper R, Munro J, Walters SJ, Snaith ML. Generic and condition-specific outcome measures for people with osteoarthritis of the knee. *Rheumatology* 1999; **38**: 870–7.

16 Guyatt GH, Bombardier C, Tugwell PX. Measuring disease-specific quality of life in clinical trials. *CMAJ* 1986;

For peer review only

134: 889–95.

17 Mokkink LB, Terwee CB, Stratford PW, *et al.* Evaluation of the methodological quality of systematic reviews of health status measurement instruments. *Qual Life Res* 2009; **18**: 313–33.

18 Higgins J, Green S, editors. Cochrane Handbook for Systematic Reviews of Interventions, Version 5. 2011 http://handbook.cochrane.org.

19 Moher D, Liberati A, Tetzlaff J, Altman DG. Preferred reporting items for systematic reviews and meta-analyses: the PRISMA statement. *BMJ* 2009; **339**. DOI:10.1136/bmj.b2535.

20 Mokkink LB, Terwee CB, Patrick DL, *et al.* The COSMIN study reached international consensus on taxonomy, terminology, and definitions of measurement properties for health-related patient-reported outcomes. *J Clin Epidemiol* 2010; **63**: 737–45.

21 Mokkink LB, Terwee CB, Knol DL, *et al.* The COSMIN checklist for evaluating the methodological quality of studies on measurement properties: a clarification of its content. *BMC Med Res Methodol* 2010; **10**: 22.

22 Briggs KK, Kocher MS, Rodkey WG, Steadman JR. Reliability, validity, and responsiveness of the Lysholm knee score and Tegner activity scale for patients with meniscal injury of the knee. *J Bone Joint Surg Am* 2006; **88**: 698–705.

23 Terwee CB, Bot SDM, de Boer MR, *et al.* Quality criteria were proposed for measurement properties of health status questionnaires. *J Clin Epidemiol* 2007; **60**: 34–42.

24 Terwee CB, Jansma EP, Riphagen II, De Vet HCW. Development of a methodological PubMed search filter for finding studies on measurement properties of measurement instruments. *Qual Life Res* 2009; **18**: 1115–23.

25 Terwee CB, Mokkink LB, Knol DL, Ostelo RWJG, Bouter LM, De Vet HCW. Rating the methodological quality in systematic reviews of studies on measurement properties: A scoring system for the COSMIN checklist. *Qual Life Res* 2012; **21**: 651–7.

26 Mokkink LB, Terwee CB, Patrick DL, *et al.* The COSMIN checklist for assessing the methodological quality of studies on measurement properties of health status measurement instruments: An international Delphi study. *Qual Life Res* 2010; **19**: 539–49.

27 Schellingerhout JM, Verhagen AP, Heymans MW, Koes BW, De Vet HC, Terwee CB. Measurement properties of disease-specific questionnaires in patients with neck pain: A systematic review. *Qual Life Res* 2012; **21**: 659–70.

28 Van Tulder M, Furlan A, Bombardier C, Bouter L. Updated Method Guidelines for Systematic Reviews in the Cochrane Collaboration Back Review Group. *Spine (Phila Pa 1976)* 2003; **28**: 1290–9.

29 Flandry F, Hunt JP, Terry GC, Hughston JC. Analysis of subjective knee complaints using visual analog scales. *Am J Sports Med* 1991; **19**: 112–8.

30 Goodwin PC, Ratcliffe J, Morris J, Morrissey MC. Using the knee-specific Hughston Clinic Questionnaire, EQ-5D and SF-6D following arthroscopic partial meniscectomy surgery: A comparison of psychometric properties. *Qual Life Res* 2011; **20**: 1437–46.

31 Irrgang JJ, Anderson AF, Boland AL, *et al.* Development and validation of the international knee documentation committee subjective knee form. *Am J Sports Med* 2001; **29**: 600–13.

32 Crawford K, Briggs KK, Rodkey WG, Steadman JR. Reliability, Validity, and Responsiveness of the IKDC Score

For peer review only

for Meniscus Injuries of the Knee. *Arthrosc - J Arthrosc Relat Surg* 2007; **23**: 839–44.

33 van de Graaf V a, Wolterbeek N, Scholtes V a B, Mutsaerts EL a R, Poolman RW. Reliability and Validity of the IKDC, KOOS, and WOMAC for Patients With Meniscal Injuries. *Am J Sports Med* 2014; **42**: 1408–16.

34 Roos EM, Roos HP, Lohmander LS, Ekdahl C, Beynnon BD. Knee Injury and Osteoarthritis Outcome Score (KOOS)—Development of a Self-Administered Outcome Measure. *J Orthop Sport Phys Ther* 1998; **28**: 88–96.

35 Roos EM, Roos HP, Ekdahl C, Lohmander LS. Knee injury and Osteoarthritis Outcome Score (KOOS)--validation of a Swedish version. *Scand J Med Sci Sports* 1998; **8**: 439–48.

36 E.M. R, H.P. R, C. E, L.S. L. Knee injury and Osteoarthritis Outcome Score (KOOS)--validation of a Swedish version. *Scand J Med Sci Sports* 1998; **8**: 439–48.

37 Lysholm J, Gillquist J. Evaluation of knee ligament surgery results with special emphasis on use of a scoring scale. 1982; **10**: 150–4.

38 Tegner Y, Lysholm J. Rating systems in the evaluation of knee ligament injuries. *Clin Orthop Relat Res* 1985; : 43–9.

39 Bellamy N. The WOMAC Knee and Hip Osteoarthritis Indices: Development, validation, globalization and influence on the development of the AUSCAN Hand Osteoarthritis Indices. *Clin Exp Rheumatol* 2005; **23**. DOI:10.1097/01.prs.0000436796.74305.38.

40 Group TE. EuroQol - a new facility for the measurement of health-related quality of life. *Health Policy (New York)* 1990; **16**: 199–208.

41 Garratt M, Brealey S, Robling M, *et al.* Development of the knee quality of life (KQoL-26) 26-item questionnaire: data quality, reliability, validity and responsiveness. *Health Qual Life Outcomes* 2008; **6**: 48.

42 Braizer J, Roberts J, Deverill M. The Estimation of a Preference-Based Measure of Health Form the SF-36. *Journall Heal Econ* 2002; **21**: 271–92.

43 Kirkley A, Griffin S, Whelan D. The development and validation of a quality of life-measurement tool for patients with meniscal pathology: the Western Ontario Meniscal Evaluation Tool (WOMET). *Clin J Sport Med* 2007; **17**: 349–56.

44 Wang D, Jones H, Khair M, Miniaci A. Patient-reported outcome measures for the knee. *J Knee Surg* 2010; **23**: 137–51.

45 Thorborg K, Tijssen M, Habets B, *et al.* Patient-Reported Outcome (PRO) questionnaires for young to middle-aged adults with hip and groin disability: a systematic review of the clinimetric evidence. *Br J Sports Med* 2015; **49**: 812.

46 Thoomes-de Graaf M, Scholten-Peeters GGM, Schellingerhout JM, *et al.* Evaluation of measurement properties of self-administered PROMs aimed at patients with non-specific shoulder pain and activity limitations: a systematic review. *Qual Life Res* 2016; **25**: 2141–60.

47 Green A, Liles C, Rushton A, Kyte DG. Measurement properties of patient-reported outcome measures (PROMS) in Patellofemoral Pain Syndrome: A systematic review. *Man Ther* 2014; **19**: 517–26.

48 Terwee CB, Bouwmeester W, van Elsland SL, de Vet HCW, Dekker J. Instruments to assess physical activity in patients with osteoarthritis of the hip or knee: A systematic review of measurement properties. *Osteoarthr Cartil* 2011; **19**: 620–33.

For peer review only

49 Gagnier JJ, Mullins M, Huang H, *et al.* A Systematic Review of Measurement Properties of Patient-Reported Outcome Measures Used in Patients Undergoing Total Knee Arthroplasty. *J Arthroplasty* 2017. DOI:10.1016/j.arth.2016.12.052.

50 Kroman SL, Roos EM, Bennell KL, Hinman RS, Dobson F. Measurement properties of performance-based outcome measures to assess physical function in young and middle-aged people known to be at high risk of hip and/or knee osteoarthritis: A systematic review. *Osteoarthr Cartil* 2014; **22**: 26–39.

51 Dobson F, Hinman RS, Hall M, Terwee CB, Roos EM, Bennell KL. Measurement properties of performance-based measures to assess physical function in hip and knee osteoarthritis: A systematic review. *Osteoarthr Cartil* 2012; **20**: 1548–62.

52 Tijssen M, van Cingel R, van Melick N, de Visser E. Patient-Reported Outcome questionnaires for hip arthroscopy: a systematic review of the psychometric evidence. *BMC Musculoskelet Disord* 2011; **12**: 117.

53 van der Wal RJP, Heemskerk BTJ, van Arkel ERA, Mokkink LB, Thomassen BJW. Translation and Validation of the Dutch Western Ontario Meniscal Evaluation Tool. *J Knee Surg* 2016; **1**. DOI:10.1055/s-0036-1584576.

54 Guermazi A, Niu J, Hayashi D, *et al.* Prevalence of abnormalities in knees detected by MRI in adults without knee osteoarthritis: population based observational study (Framingham Osteoarthritis Study). *BMJ* 2012; **345**: e5339.

55 Haviv B, Bronak S, Kosashvili Y, Thein R. Arthroscopic meniscectomy of traumatic versus atraumatic tears in middle aged patients: is there a difference? *Arch Orthop Trauma Surg* 2016; **136**: 1297–301.

56 Thorlund JB, Englund M, Christensen R, *et al.* Patient reported outcomes in patients undergoing arthroscopic partial meniscectomy for traumatic or degenerative meniscal tears: comparative prospective cohort study. *Bmj* 2017; **356**: j356.

57 Mokkink LB, Terwee CB, Gibbons E, *et al.* Inter-rater agreement and reliability of the COSMIN (COnsensus-based Standards for the selection of health status Measurement Instruments) Checklist. *Med Res Methodol* 2010; **10**: 1–11.

58 McKenna SP. Measuring patient-reported outcomes: moving beyond misplaced common sense to hard science. *BMC Med* 2011; **9**: 86.

59 Tornbjerg SM, Nissen N, Englund M, *et al.* Structural pathology is not related to patient-reported pain and function in patients undergoing meniscal surgery. *Br J Sports Med* 2016; : bjsports-2016-096456.

60 Thorlund JB, Juhl CB, Roos EM, Lohmander LS. Arthroscopic surgery for degenerative knee: systematic review and meta-analysis of benefits and harms. *Bmj* 2015; **350**: h2747–h2747.

61 Sihvonen R, Järvelä T, Aho H, Järvinen LN. Validation of the Western Ontario Meniscal Evaluation Tool (WOMET) for patients with a degenerative meniscal tear: a meniscal pathology-specific quality-of-life index. *J Bone Joint Surg Am* 2012; **94**.

62 Celik D, Demirel M, Kuş G, Erdil M, Özdinçler Razak A. Translation, cross-cultural adaptation, reliability and validity of the Turkish version of the Western Ontario Meniscal Evaluation Tool (WOMET). *Knee Surg Sports Traumatol Arthrosc* 2015; **23**: 816.

63 Tong WW, Wang W, Xu WD. Development of a Chinese version of the Western Ontario Meniscal Evaluation Tool: cross-cultural adaptation and psychometric evaluation. *J Orthop Surg Res* 2016; **11**: 90.

For peer review only

64 Bellamy N, Buchanan WW, Goldsmith CH, Campbell J, Stitt LW. Validation study of WOMAC: a health status instrument for measuring clinically important patient relevant outcomes to antirheumatic drug therapy in patients with osteoarthritis of the hip or knee. *J Rheumatol* 1988; **15**: 1833–40.

For peer review only

Tables

Table 1: Overall levels of evidence for the quality of the measurement property[27,28]

The quality of the evidence for the measurement property for each PROM, considering the quality criteria for each measurement property (Appendix 2), the methodology of each study reporting the measurement property (Table 4) and the number of studies reporting the measurement property including consistency of findings.

Level of Evidence	Rating	Quality Criteria
Strong	+++ or - - -	Consistent findings (positive or negative) in multiple studies of good methodological quality OR in one study of excellent methodological quality
Moderate	++ or - -	Consistent findings (positive or negative) in multiple studies of fair methodological quality OR in one study of good methodological quality
Limited	+ or -	One study of fair methodological quality (positive or negative)
Conflicting	+/-	Conflicting results
Unknown	?	Only studies of poor methodological quality

+ = positive rating, ? = indeterminate rating, - = negative rating

For peer review only

Table 2: Characteristics of the included studies

Study (year)	Instrument(s)	Country (language)	Population (inclusion and exclusion criteria)	*N*	Mean age (SD, range)	Female : Male
Goodwin (2011)[30]	**Hughston<br>EQ-5D<br>SF-6D**	**UK<br>(English)**	**Inclusion:** Patients previously undergoing arthroscopic partial meniscectomy.	84	38 (SD 8, 21-58)	14 : 86 %
Crawford (2007)[32]	**IKDC**	**USA<br>(English)**	**Inclusion:** Patient with "meniscal pathology requiring treatment" and completed IKDC questionnaire<br>**Exclusion:** Patients with ligament pathology or a chondral defect greater than Outerbridge grade 2	Groups:<br>A: 31<br>B: 264<br>C: 50<br>D: 50	48 (18-81)	29 : 71 %
Van de Graaf (2014)[33]	**IKDC<br>KOOS<br>WOMAC**	**Netherlands<br>(Dutch)**	**Inclusion:** Age >18, knowledge of Dutch language, either on waiting list for meniscal surgery or between 6 weeks and 6 months after meniscal surgery.<br>**Exclusion:** Received arthroplasty in either knee or previous ACL surgery on the knee of interest.	75	48.8 (35-62)	50 : 50 %
Roos (1998)[36]	**KOOS**	**Sweden<br>(Swedish)**	**Inclusion:** Patient waiting for knee arthroscopy for either meniscal lesion, ACL injury or tibio-femoral cartilage damage.<br>(54% meniscal tear, 20% ACL+meniscal tear, 13% ACL, 13% isolated chondral damage)<br>**Exclusion:** Multiple joint involvement, other diagnosis.	142	39.7 (14-75)	22 : 78 %
Garratt (2008)[41]	**KQoL-26**	**UK<br>(English)**	**Inclusion:** Patients aged 18-55, referred to hospital clinic with suspected meniscus or knee ligament pathology.<br>(67% meniscal tear, 30% ACL, 3% other)<br>**Exclusion:** Requiring urgent referral, non-traumatic arthropathy, chronic knee instability, previous same knee surgery (except diagnostic arthroscopy)	323	47 (14.3)	44 : 56 %
Briggs (2006)[22]	**Lysholm<br>Tegner**	**USA<br>(English)**	**Inclusion:** Patient previously undergoing surgery for meniscal lesion or waiting list for meniscal surgery.	Groups:<br>A: 122<br>B: 191<br>C: 477	40 (13-81)	32 : 68 %
Kirkley (2007)[43]	**WOMET**	**Canada<br>(English)**	**Inclusion:** Patients with "meniscal symptoms (swelling, catching, locking)" and magnetic resonance imaging suggestive of meniscal pathology.	Groups:<br>A: 31<br>B: 36<br>C: 34<br>D: 69	Not reported	Not reported
Sihvonen (2012)[61]	**WOMET**	**Finland<br>(Finnish)**	**Inclusion:** Patients with arthroscopically verified degenerative meniscal tear and no previous knee trauma.<br>**Exclusion:** Trauma, bilateral arthroscopy, re-operation within 6 months	Groups:<br>A: 485<br>B: 385<br>C: 100<br>D: 40	53 (18-81)	45 : 55 %
Celik (2013)[62]	**WOMET**	**Turkey<br>(Turkish)**	**Inclusion:** Age >16, presence of meniscal tear or previous meniscal repair or resection, complete questionnaires.<br>**Exclusion:** Ligament injury, "articular cartilage damage causing instability", inability to complete the form due to cognitive impairment.	96	43.6 (23-71)	64 : 36 %
Tong (2016)[63]	**WOMET**	**China<br>(Chinese)**	**Inclusion:** Patients with meniscal pathology who underwent arthroscopic surgery for meniscal repair or resection. Age >18, able to read and speak Chinese.<br>**Exclusion:** Ligament injuries, history of leg surgery, infection, tumours, rheumatologic disease, neurological or musculoskeletal disorders.	121	41.2 (14.3)	57 : 43 %
Van der Wal (2016)[53]	**WOMET**	**Netherlands<br>(Dutch)**	**Inclusion:** Patients with MRI confirmed, symptomatic, meniscal tear. Age 18-70, understanding of Dutch language.<br>**Exclusion:** Concomitant ligament injury, previous ligament injury with instability, previous knee surgery, chondral defect greater than Outerbridge grade 2 on MRI or during surgery, inability to participate due to cognitive impairment.	86	Median 52 (IQr 43-60)	41 : 59 %

IQr = interquartile range.

For peer review only

Table 3: Characteristics of the included PROMs

Instrument	Year of development	Original language	Intended Construct & Domains	Number of questions	Target or Development Population	Patients involved in development?
**Symptoms & Functional Status**						
Hughston	1991[29]	English	**Knee-specific symptoms, functional status, sports activity.**<br>No sub-domains	28 questions	"Patients who had undergone knee surgery that varied from arthroscopy to total arthroplasty."[29]	No
IKDC	2001[31]	English	**Knee-specific symptoms, functional status, sports activity.**<br>1. Symptoms<br>2. Sports activities<br>3. Function	18 questions	"A knee-specific, rather than a disease-specific, measure of symptoms, function, and sports activity."[31]	No
KOOS	1998[34]	English	**Knee injury-specific symptoms, functional status, sports activity, quality of life.**<br>1. Symptoms & Stiffness<br>2. Pain<br>3. Activities of daily living (ADL)<br>4. Function in sports and recreation<br>5. Knee-related quality of life (QoL)	42 questions	Patients with knee injury (ACL or meniscus injury) at risk of developing osteoarthritis.	Yes
Lysholm	1982[37] / 1985[38]	English	**Disease-specific (knee ligament) symptoms, functional status**<br>No sub-domains	8 questions	"A scoring scale for knee ligament surgery follow-up emphasizing evaluation of symptoms of instability."[37]	No
WOMAC	1982[39]	English	**Disease-specific (osteoarthritis of hip or knee) symptoms, functional status**<br>1. Pain<br>2. Stiffness<br>3. Function & Daily activities	24 questions	"Outcomes of anti-rheumatic drug therapy in patients with osteoarthritis of the hip or knee."[64]	Yes
**Health-related quality of life**						
EQ-5D	1990[40]	English, Dutch, Finnish, Norwegian, Swedish	**General population health-related quality of life**<br>1. Mobility<br>2. Self-care<br>3. Usual activities<br>4. Pain/Discomfort<br>5. Anxiety/Depression	6 questions	General tool for describing and valuing health related quality of life – items developed and valued after questioning large samples of randomly selected adults.	Yes
KQoL-26	2008[41]	English	**Disease-specific (knee ligament or meniscus) health-related quality of life**<br>1. Physical functioning<br>2. Activity limitations<br>3. Emotional functioning	26 questions	"Patients with a suspected ligamentous or meniscal injury of the knee."[41]	Yes
SF-6D	2004[42]	English	**General population health-related quality of life**<br>1. Physical functioning<br>2. Role limitation<br>3. Social functioning<br>4. Pain<br>5. Mental health<br>6. Vitality	6 questions	Derived from SF-36 or SF-12. A general, preference based classification for describing health-related quality of life.	Yes
WOMET	2007[43]	English	**Disease-specific (meniscus) health-related quality of life**<br>Physical symptoms<br>1. Sports/Recreation/Work/Lifestyle<br>2. Emotions	16 questions	"Patients with meniscal symptomology (swelling, catching, locking) and in whom magnetic resonance imaging had suggested meniscal pathology."[43]	Yes
**Activity level**						
Tegner	1985[38]	English	**Disease-specific (knee ligament) symptoms, functional status**<br>No sub-domains	1 question	Patients with ACL injury diagnosed by clinical examination under anaesthesia and confirmed by arthroscopy or arthrotomy.	No

ACL = anterior cruciate ligament

For peer review only

Table 4: Quality of each study per PROM and measurement property (COSMIN rating)

**Instrument and Study**	Internal consistency	Reliability	Measurement error	Content validity	Structural validity	Hypothesis testing	Cross-cultural validity	Responsiveness
Symptoms & Functional Status								
**Hughston**								
Goodwin (2011)[30]	Poor	na	na	Poor	na	Good	na	Poor
**IKDC**								
Crawford (2007)[32]	Poor	Fair	Fair	Poor	na	Fair	na	Poor
Van de Graaf (2014)[33]	Poor	Good	Good	Poor	Fair	Good	Poor	na
**KOOS**								
Roos (1998)[36]	Poor	Fair	na	Poor	Poor	Fair	Poor	Poor
Van de Graaf (2014)[33]	Poor	Good	Good	Poor	Poor	Good	Poor	na
**Lysholm**								
Briggs (2006)[22]	Poor	Fair	Fair	Poor	na	Fair	na	Poor
**WOMAC**								
Van de Graaf (2014)[33]	Poor	Good	Good	Poor	Poor	Good	Poor	na
Health-related quality of life								
**EQ-5D**								
Goodwin (2011)[30]	Poor	na	na	Poor	na	Good	na	Poor
**KQoL-26**								
Garratt (2008)[41]	Fair	Fair	na	Fair	Fair	Fair	na	Poor
**SF-6D**								
Goodwin (2011)[30]	Poor	na	na	Poor	na	Good	na	Poor
**WOMET**								
Kirkley (2007)[43]	Poor	Fair	na	Excellent	na	Fair	na	Fair
Sihvonen (2012)[61]	Poor	Poor	na	Poor	na	Fair	Poor	Poor
Celik (2013)[62]	Poor	Good	Good	Poor	na	Good	Poor	na
Tong (2016)[63]	Poor	Good	na	Poor	na	Good	Poor	Poor
Van der Wal (2016)[53]	Poor	Good	Good	Good	na	Good	Poor	Good
Activity level								
**Tegner**								
Briggs (2006)[22]	na	Fair	Fair	Poor	na	Fair	na	Poor

For peer review only

Table 5: Interpretability including missing items, response rate, floor and ceiling effects

Instrument and Study	Administration	Missing responses	Missing items	Overall % lowest possible total score (floor)	Overall % highest possible score (ceiling)	Items or Domains with >15% responses with lowest score (floor)	Items or Domains >15% highest possible score (ceiling)	MIC
**Symptoms & Functional Status**								
**Hughston**								
Goodwin (2011)[30]	Clinic	Not reported	Not reported	0%	0%	Not reported	Not reported	Not reported
**IKDC**								
Crawford (2007)[32]	Clinic / Postal	Not reported	Not reported	0%	0%	• Activity pain<br>• Pain last 4 weeks<br>• Pain severity<br>• Catching<br>• Kneeling<br>• Sitting<br>• Running<br>• Jumping<br>• Stopping	• Swelling<br>• Catching<br>• Climb stairs<br>• Sitting<br>• Rising	Not reported
Van de Graaf (2014)[33]	Online / Postal	Unclear	0%	0%	0%	Nil	Nil	Not reported
**KOOS**								
Roos (1998)[36]	Postal	7.2%	0.8%	Not reported	Not reported	Nil	Nil	Not reported
Van de Graaf (2014)[33]	Online / Postal	Unclear	0%	0%	3%	Nil	Nil	Not reported
**Lysholm**								
Briggs (2006)[22]	Clinic	Not reported	Not reported	0%	0.5%	• Squatting<br>• Pain	• Swelling<br>• Instability<br>• Support<br>• Limp<br>• Locking	Not reported
**WOMAC**								
Van de Graaf (2014)[33]	Online / Postal	Unclear	0%	0%	6%	Nil	Nil	Not reported
**Health-related quality of life**								
**EQ-5D**								
Goodwin (2011)[30]	Clinic	Not reported	Not reported	4%	1%	Not reported	Not reported	Not reported
**KQoL-26**								
Garratt (2008)[41]	Postal	41%	14.9%	Not reported	Not reported	• Avoiding turning, twisting, or sideways movements<br>• *	• Staying seated for 15 minutes<br>• *	Not reported
**SF-6D**								
Goodwin (2011)[30]	Clinic	Not reported	Not reported	0%	0%	Not reported	Not reported	Not reported
**WOMET**								
Kirkley (2007)[43]	Clinic	Not reported	Not reported	5.7%	1.7%	Not reported	Not reported	Not reported
Sihvonen (2012)[61]	Unclear	16%	7.5%	0%	0%	• Numbness	Nil	Not reported
Celik (2013)[62]	Unclear	Not reported	Not reported	0%	0%	• Numbness<br>• Swelling	• Consciousnes s<br>• Activities<br>• Specific skills<br>• Squatting<br>• Fear injury<br>• Concern about future of knee<br>• Frustration	Not reported
Tong (2016)[63]	Unclear	Not reported	0%	0%	0%	Nil	Nil	Not reported
Van der Wal (2016)[53]	Clinic	0%	<1%	0%	0%	• Numbness<br>• Swelling	Nil	14.7
**Activity level**								
**Tegner**								
Briggs (2006)[22]	Clinic	Not reported	Not reported	2.5%	2.5%	na	na	Not reported

MIC = minimal important change; * = Other domains not reported

Table 6: Overall rating of measurement properties and level of evidence for each PROM

See table 1 for a summary of the rating methodology.

Instrument	Internal consistency	Reliability	Measurement error	Content validity	Structural validity	Hypothesis testing	Cross-cultural validity	Responsiveness
**Symptoms & Functional Status**								
**Hughston**								
English[30]	?	na	na	?	na	- -	na	?
**IKDC**								
English[32]	?	+	?	?	na	+	na	?
Dutch[33]	?	++	?	?	-	++	?	na
**KOOS**								
Dutch[33]	?	++	?	?	?	++	?	na
Swedish[36]	?	+	na	?	?	+	?	?
**Lysholm**								
English[22]	?	+	?	?	na	+	na	?
**WOMAC**								
Dutch[33]	?	++	?	?	?	++	?	na
**Health-related quality of life**								
**EQ-5D**								
English[30]	?	na	na	?	na	++	na	?
**KQoL-26**								
English[41]	+	+	na	+	+	+	na	?
**SF-6D**								
English[30]	?	na	na	?	na	++	na	?
**WOMET**								
English[43]	?	+	na	+++	na	+	na	+
Chinese[63]	?	++	na	?	na	++	?	?
Dutch[53]	?	++	- -	++	na	++	?	++
Finnish[61]	?	?	na	?	na	+	?	?
Turkish[62]	?	++	?	?	na	++	?	na
**Activity level**								
**Tegner**								
English[22]	na	+	?	?	na	+	na	?

Figure legends

Figure 1: PRISMA Flow Diagram
Overview of study selection. Full search strategy may be found in supplementary appendix 1.

For peer review only

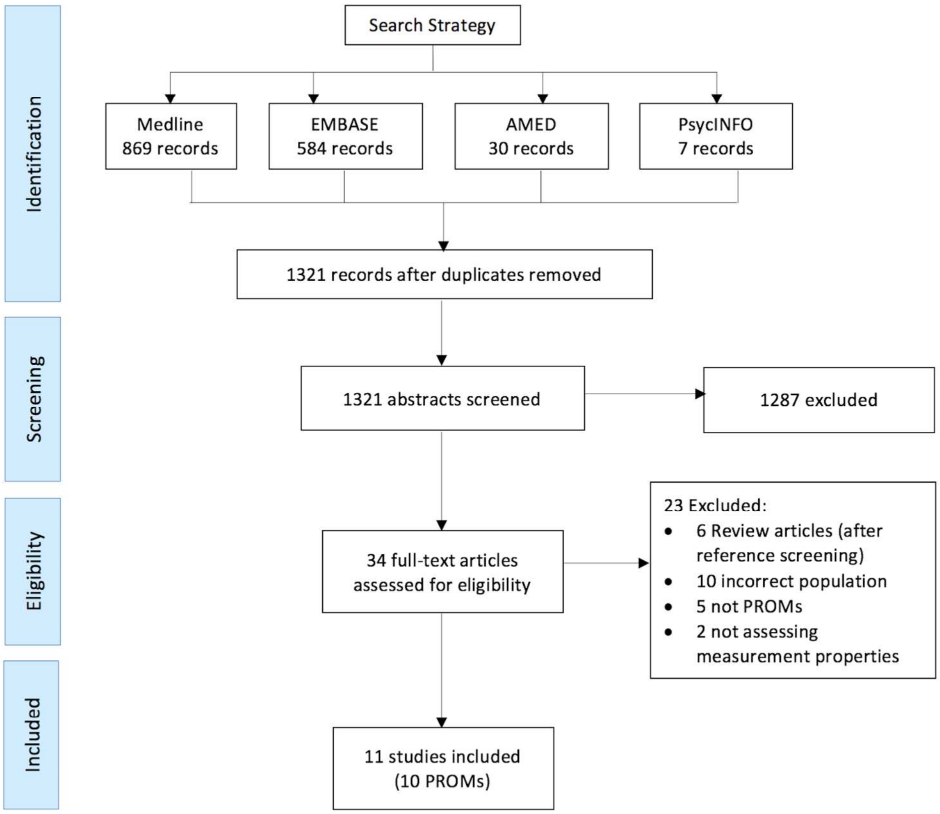

Figure 1: PRISMA Flow Diagram‼ ┼ ‼ ┼

Overview of study selection.

‼ ┼ ‼ ┼ Full search strategy may be found in supplementary appendix 1.

150x123mm (300 x 300 DPI)

Appendix 1: Search strategy

Databases: MEDLINE, Embase, AMED, PsycInfo

#1 Condition

menis*.af

#2 Construct

("quality of life" OR qol OR func* OR HR-PRO OR HRPRO OR HRQOL OR QL OR disab* OR wellbeing OR "well being" OR subjective OR utility OR utilities OR priorit* OR outcome* OR health).af

#3 Instrument

(score* OR measure* OR PROM OR index* OR indices OR scale* OR questionnaire* OR instrument* OR survey* OR profile* OR apprais* OR status OR reported OR reporting OR rated OR rating* OR assessment*).af

#4 Measurement Properties

"Validation Studies".pt OR instrumentation.af OR ("observer variation" OR "psychometrics" OR "reproducibility of results" OR "discriminant analysis").mh OR (agreement OR precision OR imprecision OR "precise values" OR repeatab* OR ((replicab* OR repeated) AND (measure OR measures OR findings OR result OR results OR test OR tests))).af OR (reproducib* OR psychometr* OR clinimetr* OR clinometr* OR observer AND variation OR reliab* OR valid* OR coefficient OR "internal consistency" OR (cronbach* AND (alpha OR alphas)) OR "item correlation" OR "item correlations" OR "item selection" OR "item selections" OR "item reduction" OR "item reductions" OR test?retest OR (test AND retest) OR (reliab* AND (test OR retest)) OR stability OR interrater OR inter-rater OR intrarater OR intra-rater OR intertester OR inter-tester OR intratester OR intra-tester OR interobserver OR inter-observer OR intraobserver OR intra-observer OR intertechnician OR intertechnician OR intratechnician OR intra-technician OR interexaminer OR inter-examiner OR intraexaminer OR intra-examiner OR inter-assay OR inter-assay OR intraassay OR intra-assay OR interindividual OR inter-individual OR intraindividual OR intra-individual OR interparticipant OR inter-participant OR intraparticipant OR intra-participant OR kappa OR kappa?s OR "coefficient of variation" OR generaliza* OR generalisa* OR concordance OR (intraclass AND correlation*) OR discriminative OR "known group" OR "factor analysis" OR "factor analyses" OR "factor structure" OR "factor structures" OR dimensionality OR subscale* OR "multitrait scaling analysis" OR "multitrait scaling analyses" OR "item discriminant" OR "interscale correlation" OR "interscale correlations" OR ((error OR errors) AND (measure* OR correlat* OR evaluat* OR accuracy OR accurate OR precision OR mean)) OR "individual variability" OR "interval variability" OR "rate variability").ti,ab NOT ("addresses" OR "biography" OR "case reports" OR "comment" OR "directory" OR "editorial" OR "festschrift" OR "interview" OR "lectures" OR "legal cases" OR "legislation" OR "letter" OR "news" OR "newspaper article" OR "patient education handout" OR "popular works" OR "congresses" OR "consensus development conference" OR "consensus development conference, nih" OR "practice guideline").pt

#5 (#1 AND #2 AND #3 AND #4)

#5 Remove Duplicates

Appendix 2: Quality criteria for measurement properties[1]

Property	Rating	Quality Criteria
**Reliability**		
Internal consistency	+	Cronbach's alpha(s) ≥ 0.70
	?	Cronbach's alpha not determined or dimensionality unknown
	-	Cronbach's alpha(s) < 0.70
Reliability	+	ICC / weighted Kappa ≥ 0.70 OR Pearson's r ≥ 0.80
	?	Neither ICC / weighted Kappa, nor Pearson's r determined
	-	ICC / weighted Kappa < 0.70 OR Pearson's r < 0.80
Measurement error	+	MIC > SDC OR MIC outside the LOA
	?	MIC not defined
	-	MIC ≤ SDC OR MIC equals or inside LOA
**Validity**		
Content validity	+	All items are considered to be relevant for the construct to be measured, for the target population, and for the purpose of the measurement AND the questionnaire is considered to be comprehensive
	?	Not enough information available
	-	Not all items are considered to be relevant for the construct to be measured, for the target population, and for the purpose of the measurement OR the questionnaire is considered not to be comprehensive
Construct validity – Structural validity	+	Factors should explain at least 50% of the variance
	?	Explained variance not mentioned
	-	Factors explain < 50% of the variance
Construct validity – Hypothesis testing	+	Correlations with instruments measuring the same construct ≥ 0.50 OR at least 75% of the results are in accordance with the hypotheses AND correlations with related constructs are higher than with unrelated constructs
	?	Solely correlations determined with unrelated constructs
	-	Correlations with instruments measuring the same construct < 0.50 OR < 75% of the results are in accordance with the hypotheses OR correlations with related constructs are lower than with unrelated constructs
Construct validity – Cross-cultural validity	+	No differences in factor structure OR no important DIF between language versions
	?	Multiple group factor analysis not applied AND DIF not assessed
	-	Differences in factor structure OR important DIF between language versions
**Responsiveness**		
Responsiveness	+	Correlation with changes on instruments measuring the same construct ≥ 0.50 OR at least 75% of the results are in accordance with the hypotheses OR AUC ≥ 0.70 AND correlations with changes in related constructs are higher than with unrelated constructs
	?	Solely correlations determined with unrelated constructs
	-	Correlations with changes on instruments measuring the same construct < 0.50 OR < 75% of the results are in accordance with the hypotheses OR AUC < 0.70 OR correlations with changes in related constructs are lower than with unrelated constructs

MIC = minimal important change, SDC = smallest detectable change, LoA = limits of agreement, ICC = intraclass correlation coefficient, DIF = differential item functioning, AUC = area under the curve
+ = positive rating, ? = indeterminate rating, - = negative rating

1 Terwee CB, Bot SDM, de Boer MR, *et al.* Quality criteria were proposed for measurement properties of health status questionnaires. *J Clin Epidemiol* 2007; **60**: 34–42.

For peer review only

PRISMA 2009 Checklist

Section/topic	#	Checklist item	Reported on page #
**TITLE**			
Title	1	Identify the report as a systematic review, meta-analysis, or both.	1
**ABSTRACT**			
Structured summary	2	Provide a structured summary including, as applicable: background; objectives; data sources; study eligibility criteria, participants, and interventions; study appraisal and synthesis methods; results; limitations; conclusions and implications of key findings; systematic review registration number.	2-3
**INTRODUCTION**			
Rationale	3	Describe the rationale for the review in the context of what is already known.	3
Objectives	4	Provide an explicit statement of questions being addressed with reference to participants, interventions, comparisons, outcomes, and study design (PICOS).	3
**METHODS**			
Protocol and registration	5	Indicate if a review protocol exists, if and where it can be accessed (e.g., Web address), and, if available, provide registration information including registration number.	4
Eligibility criteria	6	Specify study characteristics (e.g., PICOS, length of follow-up) and report characteristics (e.g., years considered, language, publication status) used as criteria for eligibility, giving rationale.	4
Information sources	7	Describe all information sources (e.g., databases with dates of coverage, contact with study authors to identify additional studies) in the search and date last searched.	7
Search	8	Present full electronic search strategy for at least one database, including any limits used, such that it could be repeated.	7 + Appendix1
Study selection	9	State the process for selecting studies (i.e., screening, eligibility, included in systematic review, and, if applicable, included in the meta-analysis).	7
Data collection process	10	Describe method of data extraction from reports (e.g., piloted forms, independently, in duplicate) and any processes for obtaining and confirming data from investigators.	7
Data items	11	List and define all variables for which data were sought (e.g., PICOS, funding sources) and any assumptions and simplifications made.	4-7
Risk of bias in individual studies	12	Describe methods used for assessing risk of bias of individual studies (including specification of whether this was done at the study or outcome level), and how this information is to be used in any data synthesis.	7
Summary measures	13	State the principal summary measures (e.g., risk ratio, difference in means).	8
Synthesis of results	14	Describe the methods of handling data and combining results of studies, if done, including measures of consistency (e.g., I^2) for each meta-analysis.	8

For peer review only

PRISMA 2009 Checklist

Section/topic	#	Checklist item	Reported on page #
Risk of bias across studies	15	Specify any assessment of risk of bias that may affect the cumulative evidence (e.g., publication bias, selective reporting within studies).	8
Additional analyses	16	Describe methods of additional analyses (e.g., sensitivity or subgroup analyses, meta-regression), if done, indicating which were pre-specified.	n/a
**RESULTS**			
Study selection	17	Give numbers of studies screened, assessed for eligibility, and included in the review, with reasons for exclusions at each stage, ideally with a flow diagram.	9 + Fig 1
Study characteristics	18	For each study, present characteristics for which data were extracted (e.g., study size, PICOS, follow-up period) and provide the citations.	9 + Table 2 (p24)
Risk of bias within studies	19	Present data on risk of bias of each study and, if available, any outcome level assessment (see item 12).	9 + Table 4,6 (p26,p28)
Results of individual studies	20	For all outcomes considered (benefits or harms), present, for each study: (a) simple summary data for each intervention group (b) effect estimates and confidence intervals, ideally with a forest plot.	n/a + Table 5 (p27)
Synthesis of results	21	Present results of each meta-analysis done, including confidence intervals and measures of consistency.	9-12 + Table 6 (p28)
Risk of bias across studies	22	Present results of any assessment of risk of bias across studies (see Item 15).	9-12 + Table 6 (p28)
Additional analysis	23	Give results of additional analyses, if done (e.g., sensitivity or subgroup analyses, meta-regression [see Item 16]).	n/a
**DISCUSSION**			
Summary of evidence	24	Summarize the main findings including the strength of evidence for each main outcome; consider their relevance to key groups (e.g., healthcare providers, users, and policy makers).	13-14, 15
Limitations	25	Discuss limitations at study and outcome level (e.g., risk of bias), and at review-level (e.g., incomplete retrieval of identified research, reporting bias).	14-15
Conclusions	26	Provide a general interpretation of the results in the context of other evidence, and implications for future research.	16
**FUNDING**			

For peer review only - http://bmjopen.bmj.com/site/about/guidelines.xhtml

PRISMA 2009 Checklist

Funding	27	Describe sources of funding for the systematic review and other support (e.g., supply of data); role of funders for the systematic review.	17

From: Moher D, Liberati A, Tetzlaff J, Altman DG, The PRISMA Group (2009). Preferred Reporting Items for Systematic Reviews and Meta-Analyses: The PRISMA Statement. PLoS Med 6(7): e1000097. doi:10.1371/journal.pmed1000097

For more information, visit: www.prisma-statement.org.

Page 2 of 2

For peer review only

BMJ Open

Patient-reported outcome measures for patients with meniscal tears: a systematic review of measurement properties and evaluation with the COSMIN checklist

Journal:	*BMJ Open*
Manuscript ID	bmjopen-2017-017247.R2
Article Type:	Research
Date Submitted by the Author:	23-Aug-2017
Complete List of Authors:	Abram, Simon; University of Oxford Nuffield Department of Orthopaedics Rheumatology and Musculoskeletal Sciences,<br>Middleton, Robert; University of Oxford Nuffield Department of Orthopaedics Rheumatology and Musculoskeletal Sciences<br>Beard, David; Oxford University, Nuffield Dept of Orthopaedics, Rheumatology and Musculoskeletal Sciences<br>Price, Andrew; University of Oxford, Nuffield Department of Orthopaedics, Rheumatology and Musculoskeletal Sciences<br>Hopewell, Sally; University of Oxford, Oxford Clinical Trials Research Unit
**Primary Subject Heading**:	Surgery
Secondary Subject Heading:	Sports and exercise medicine, Patient-centred medicine
Keywords:	Knee < ORTHOPAEDIC & TRAUMA SURGERY, ORTHOPAEDIC & TRAUMA SURGERY, Orthopaedic & trauma surgery < SURGERY

SCHOLARONE™ Manuscripts

For peer review only

Patient-reported outcome measures for patients with meniscal tears: a systematic review of measurement properties and evaluation with the COSMIN checklist

S.G.F. Abram, R. Middleton, D.J. Beard, A.J. Price, S. Hopewell.

Authors' affiliation:

Nuffield Department of Orthopaedics, Rheumatology and Musculoskeletal Sciences, University of Oxford

Corresponding author:

Simon Abram

simon.abram@doctors.org.uk

Keywords:

outcomes; patient reported outcomes; psychometrics; meniscal pathology; meniscus; knee surgery; knee

For peer review only

Abstract

Objective: Meniscal tears occur frequently in the population and the most common surgical treatment, arthroscopic partial meniscectomy, is performed in approximately 2 million cases worldwide each year. The purpose of this systematic review is to summarise and critically appraise the evidence for the use of patient-reported outcome measures (PROMs) in patients with meniscal tears.

Design: A systematic review was undertaken. Data on reported measurement properties was extracted and the quality of the studies appraised according to Consensus-based Standards for the selection of health Measurement Instruments (COSMIN).

Data sources: A search of MEDLINE, Embase, AMED and PsycINFO, unlimited by language or publication date (last search 20/02/2017).

Eligibility criteria for selecting studies: Development and validation studies reporting the measurement properties of PROMs in patients with meniscal tears were included.

Results: 11 studies and 10 PROMs were included. The overall quality of studies was poor. For measurement of symptoms and functional status there is only very limited evidence supporting the selection of either the Lysholm knee scale, International Knee Documentation Committee (IKDC) Subjective Knee Form, or the Dutch-version of the Knee injury and Osteoarthritis Outcome Score (KOOS). For measuring health-related quality of life, only limited evidence supports the selection of the Western Ontario Meniscal Evaluation Tool (WOMET). Of all the PROMs evaluated, WOMET has the strongest evidence for content validity.

Conclusion: For patients with meniscal tears, there is poor quality and incomplete evidence regarding the validity of the currently available PROMs. Further research is required to ensure these PROMs truly reflect the symptoms, function, and quality of life of patients with meniscal tears.

PROPERO registration number: CRD42017056847

For peer review only

Strengths and limitations of this study

- This is the first review of PROMs for patients with meniscal tears and the first to apply the COSMIN checklist, which is a validated and accepted tool for the appraisal of study quality.
- Another strength of this review is the use of a validated, highly-sensitive search strategy to identify relevant studies. A limitation, however, is that only studies specifically designed to appraise the measurement properties of PROMs were included. Trials and other clinical studies of patients with meniscal tears were not included as these studies are not designed to assess measurement properties and the reporting of these properties would be highly unusual.
- Although the COSMIN checklist has acceptable inter-rater and intra-rater properties, the scoring of some items is reliant on author judgement. We performed pre-testing to ensure scoring consistency, and review authors scored studies independently. Nevertheless, it is feasible that another review team might score some items differently.
- For practical purposes, we chose to include tentative summary guidance regarding the selection of PROMs for use in the target population. It should be understood, however, that it would be reasonable to declare that the overall level of evidence for any of the PROMs is insufficient for a recommendation to be made.

For peer review only

Introduction

The menisci are fibrocartilaginous structures within the knee joint which are important for load distribution and knee stability.[1,2] More than one third of people over the age of 50 without any radiographic evidence of osteoarthritis may develop a 'tear' of the meniscus and over 70% of those with osteoarthritis will also have a torn meniscus.[3] These meniscal tears may be associated with significant knee pain and other symptoms, especially if the torn meniscal tissue interferes with the normal articulation of the joint.[4] Meniscal tears are diagnosed and managed based upon a combination of a review of symptoms, clinical examination, and imaging findings.[5] Arthroscopic partial meniscectomy is a surgical procedure commonly used to treat symptomatic meniscal tears with approximately two million cases performed worldwide each year with combined costs of several billion US dollars.[6] A number of recent randomised controlled trials have been published challenging the effectiveness of arthroscopic partial meniscectomy.[7–11] Patient-reported outcome measures (PROMs) are critical to the interpretation of these trials, yet a wide array of different PROMs have been collected. This inconsistency leads to restricted comparisons between trials and the best PROM for this population is unknown.[12,13]

Patient reported outcome measures (PROMs) are collected in a range of settings and are increasingly important in clinical practice. In orthopaedics, PROMs are important for auditing treatment outcomes and increasingly to demonstrate the cost-effectiveness of treatment.[14] With the rapid increase in usage, it is important to ensure that PROMs have formally validated measurement properties. Although generic PROMs enable the comparison of patients with different conditions, these PROMs may fail to capture important items in specific populations.[15] Ideally, a PROM should either be developed with condition-specific patient involvement or subsequently studied for validity in the population of interest.[16] Fundamentally, a PROM should comprehensively and consistently reflect the intended 'construct' to be measured in the population with the condition of interest – for example, health-related quality of life in patients with meniscal tears.[17]

There is a need for the selection of standardised 'core' PROMs for consistent use in clinical trials and the general clinical evaluation of patients with specific conditions.[13] A systematic review of the evidence is an important step in the selection of such a 'core outcome set' and may determine the need for further validation studies or even the development of a new PROM.[13] No systematic review has been published evaluating the measurement properties with the quality of evidence for the PROMs that are available for patients with meniscal tears. This is a barrier to the interpretation of previous research and to the design of future studies in these patients.

The purpose of this review is to report the measurement properties and evidence for the validity of all PROMs which have been evaluated in patients with meniscal tears.

For peer review only

Methods

This systemic review is reported based upon the Preferred Reporting Items for Systematic Review and Meta-Analyses (PRISMA) guidelines.[18] The protocol for this review was submitted to PROSPERO (CRD42017056847) on 20/02/2017.

Study selection criteria

We included studies of adults with meniscal tears of the knee. Those studies with less than 50% of patients having a meniscal tear as the primary diagnosis (i.e. without other significant knee pathology e.g. concomitant anterior cruciate ligament (ACL) rupture) were excluded unless the meniscal tear group was reported separately.
Studies administrating PROMs for the purpose of assessing measurement properties were included. PROMs using standard scoring methods, without clinician completed elements, measuring health related quality of life, health status, symptoms including pain, or functional status were included. Some studies included patients undergoing surgery (e.g. arthroscopic partial meniscectomy, meniscal repair) as part of this assessment process, but the purpose of this review was not to assess the effectiveness of such interventions.

Measurement properties

All PROM measurement properties reported by the included studies were evaluated. The primary measurement properties assessed were those within the reliability, validity, and responsiveness domains. The secondary domains assessed were interpretability and generalisability. The Consensus-based Standards for the selection of health Measurement Instruments (COSMIN) definition of the domains and measurement properties follows below.[19]

Reliability

The reliability domain is a measure of how free a PROM is from measurement error.[19] The measurement properties within this domain are assessed by repeated collection of the PROM in a defined period when there has been no change in the patient's condition. Ideally, rather than assume the patient's condition is unchanged, a methodologically strong study will assess for change, for example by administering a knee-specific global transition question on symptoms.

- Internal consistency: is the degree of inter-relatedness among the PROM items.[19]
- Reliability: is the proportion of total variance in the measurement which is because of true differences among patients.[19]
- Measurement error: is the systematic and random error of a patient's score that is not attributed to true changes in the construct to be measured.[19]

For peer review only

Validity

The validity domain is the extent to which the PROM measures the 'construct' it purports to measure.[19]

- Content validity: is the degree to which the content of a PROM is an adequate reflection of the construct to be measured.[19] The items should be comprehensive and relevant.
- Construct validity: is the degree to which the scores of a PROM are consistent with hypotheses (e.g. relationship of the score to that of other PROMs collected in the same group) based on the assumption the PROM validly measures the intended construct.[19]
    - Structural validity: is the degree to which the scores of a PROM are an adequate reflection of the dimensionality of the construct to be measured.[19]
    - Hypothesis testing: assumes that the PROM validly measures the construct of interest. Hypotheses are prepared *a priori* with regards to the correlation of the PROM with other relevant PROMs or domains of other PROMs. The magnitude and direction of the correlation should be stated in advance of testing.
    - Cross-cultural validity: is the degree to which the performance of the items on a translated or culturally adapted PROM are comparable to the performance of the original version of the PROM.[19]

Responsiveness

Responsiveness is defined as the ability of a PROM to measure change over time in the construct to be measured.[19] It is important to note that in studies assessing the measurement properties of a PROM, responsiveness should be assessed against another valid PROM as for the assessment of construct validity. Measurement of effect size alone is not appropriate as this is a measure of the magnitude of the change and not the quality of the measurement.[20]

Interpretability

Interpretability is defined as the degree to which it is possible to assign qualitative meaning to a PROM's quantitative score.[19] It is not considered a measurement property but is important when interpreting the findings from administration of a PROM in the context of a clinical condition. Interpretability includes an assessment of minimal important change (MIC), floor and ceiling effects. In general, floor and ceiling effects <15% are considered acceptable although some authors have argued the threshold should be set at <30%.[21,22] A high floor or ceiling effect suggests that items at the lower or upper end are missing from a question item, domain, or the PROM overall.

Generalisability

Generalisability is an assessment of external validity: the extent to which the findings on the measurement properties of a PROM may be considered relevant to a population or construct of interest. For example, a study of the measurement properties of a PROM in a population with advanced knee osteoarthritis cannot be generalised to

For peer review only

athletes with knee ligament injury without further study in the target population. In this review, the population of patients involved in the original development of each PROM is determined and the inclusion and exclusion criteria of all studies reporting measurement properties of the included PROMs is reported to highlight any heterogeneity. The generalisability of findings to the population of patients with meniscal tears is considered.

Search Strategy

We performed a search of MEDLINE, Embase, AMED and PsycINFO, unlimited by language or publication date. The search was based upon a validated search filter designed to be highly sensitive in identifying all studies of measurement properties.[23] Full details of the search are available in supplementary appendix 1. The final search was performed on 20/02/2017, following submission of the protocol to PROSPERO. A review of study citations was performed to further increase the sensitivity of the search strategy.

Selection of studies

The title and abstract of all records retrieved by the search was independently reviewed by two authors against the inclusion and exclusion criteria (SA and RM). Any disagreement was resolved with review of the full text publication and discussion. Referral to a third author (SH) was not required for agreement. The original PROM development article was retrieved for all PROMs identified – for example, where a PROM was developed for a condition other than meniscal pathology and subsequently tested in a population with meniscal tears.

Data extraction: Measurement properties and assessing the quality of studies

Data extraction was performed by two authors (SA and RM) and any disagreement resolved in consultation with a third author (SH). The following was extracted from each publication: the PROM, the intended construct for measurement, measurement properties, administration method, study population and diagnosis, number of patients, patient demographics, country, language and setting and method of administration (e.g. postal, online).

The quality of each included study was assessed by two reviewers (SA and RM) using the Consensus-based Standards for the selection of health Measurement Instruments (COSMIN) appraisal checklist.[24] When reviewing a study of a PROM, it is necessary to consider a combination of the reported measurement properties, the patient population, and the quality of the study methodology. To help overcome some of the difficulties in evaluating the quality of PROMs, COSMIN was published in 2010.[20,25] COSMIN contains rules for grading overall methodological quality of studies performed into the measurement properties of PROMs. These consensus standards are regularly reviewed and revised based on the latest evidence and research. COSMIN initially separated standards into boxes including a series of binary methodological ratings. The scoring methodology was subsequently revised to a four level (excellent/good/fair/poor) rating system in 2012.[24] Each measurement property is assessed by a box containing 5-18 questions scored on this scale according to defined COSMIN criteria. A system of 'worst score counts' applies for

For peer review only

each box – that is, if one question in the box is scored as poor, the overall quality of the evidence for that measurement property is determined to be poor.

Data synthesis

Data synthesis was performed by SA and checked by RM. For each included PROM, a summary of the features of the PROM is presented including details of the original development process, the development population and target construct to be measured.

For each PROM, a rating (positive, negative or indeterminate) for the measurement properties reported in the study was first determined based upon consensus standards described in supplementary appendix 2.[22] This assessment was then combined with an overall quality of evidence assessment which was adapted for COSMIN from the work of the Cochrane Back Review Group (Table 1).[26,27] For example, one good quality study reporting positive measurement properties (e.g. internal consistent with Cronbach's alpha ≥ 0.70) results in an overall rating of 'moderate' (++). Where the quality of study methodology on a measurement property is rated 'poor' the overall rating of the measurement property is always rated 'indeterminate', irrespective of the number of such studies and whether the reported measurement property itself would otherwise be considered positively. These standards are designed to ensure reported measurement properties are interpreted in the context of study quality and overall reliability.

For peer review only

Results

Selection of studies

The search strategy identified 1321 unique articles for screening. After screening, 34 full text articles were retrieved of which 11 met the inclusion criteria for this review. Figure 1 summarises the study selection process. The 11 studies reported measurement properties for 10 PROMs.

Study characteristics

The characteristics of the included studies are shown in Table 2. The mean age of patients included in the studies ranged from 38-53 years. The proportion of female patients included ranged from 14-64%. Inclusion and exclusion criteria were inconsistent with regards to age, symptoms, investigations and treatment (Table 2). The development and features of the included PROMs are summarised below and in Table 3.

Quality of the included studies

In total, the 11 studies reported 93 measurement properties for the 10 PROMs. The COSMIN methodology rating for 49 of these (53%) was poor. Many measurement properties were not reported and there was inconsistent reporting between studies (Table 4).

Quality of PROMs

Interpretability factors including floor and ceiling effects are summarised in Table 5. The overall level of evidence for the measurement properties of each PROM is summarised in Table 6. This combines the rating of the reported measurement property using the consensus criteria available in supplementary appendix 2 with the COSMIN scoring and the number of studies per PROM (as described in Table 1).

Of the 10 PROMs identified, five intended to measure symptoms and functional status, four health-related quality of life and one activity level.

Symptoms & Functional Status

Hughston: The Hughston Clinic Questionnaire was developed in 1991 as a knee-specific rather than disease-specific outcome measure.[28] It includes questions on symptoms, functional status and sports activity and patients were not involved in the development of the questions. Only one study has evaluated use of the Hughston questionnaire in patients with meniscal tears.[29] Content validity was rated poor as patients were not involved in the original development and content validity has not been subsequently assessed in patients with meniscal tears. In patients

For peer review only - http://bmjopen.bmj.com/site/about/guidelines.xhtml

with meniscal tears, there was moderate negative evidence against construct validity based on hypothesis testing and all other measurement properties were either not reported or indeterminate due to poor study design or reporting (Table 6).

IKDC: The International Knee Documentation Committee (IKDC) Subjective Knee Form was developed in 2001 as a knee-specific rather than disease-specific outcome measure.[30] It includes question domains on symptoms, functional status and sports activity and patients were not involved in the development of the questions. Two studies have evaluated use of the IKDC score in patients with meniscal tears.[31,32] In English, there is limited positive evidence for reliability and construct validity based on hypothesis testing.[31] In Dutch, there is moderate positive evidence for reliability and construct validity based on hypothesis testing but limited negative evidence against structural validity.[32] In both studies, all other measurement properties were either not reported or indeterminate (Table 6). For the English-version, although no floor or ceiling effect was detected for the overall score, unacceptable floor effects were reported for 9 items and unacceptable ceiling effects in 5 items (Table 5).

KOOS: The Knee injury and Outcome Osteoarthritis Score (KOOS) was developed in 1998 as a knee-injury specific outcome measure for patients at risk of developing osteoarthritis.[33] It includes question domains on symptoms, functional status, sports activity and quality of life. Patients with anterior cruciate ligament (ACL) or meniscal injuries were included in the development process. The KOOS includes the WOMAC osteoarthritis score in full and the WOMAC may therefore be calculated from the KOOS. The KOOS has been studied in Dutch and Swedish for patients with meniscal tears; no study has evaluated the English-version of KOOS in this population.[32,34] There is moderate positive evidence for reliability and construct validity from hypothesis testing of the Dutch-version.[32] For the Swedish-version, there is limited positive evidence for reliability and construct validity based on hypothesis testing.[34] For both the Dutch and Swedish versions, content validity and all other measurement properties were either rated indeterminate or were not reported (Table 6).

Lysholm: The Lysholm knee score was developed in 1982 and modified in 1985 as a disease-specific outcome measure for patients with knee ligament injury.[35,36] The Lysholm knee score was originally designed to be completed by clinicians and developed without patient involvement. One study has evaluated the use of Lysholm in English speaking patients with meniscal tears.[21] There is limited positive evidence for reliability and construct validity based on hypothesis testing. Content validity and all other measurement properties are either indeterminate or were not reported (Table 6). There was no floor or ceiling effect for the Lysholm score overall however an unacceptable floor effect was detected for 2 items and unacceptable ceiling effects for 5 items (Table 5).

WOMAC: The Western Ontario McMaster Osteoarthritis Index (WOMAC) was developed in 1982 as a disease-specific outcome measure for patients with osteoarthritis of the hip or knee.[37] The WOMAC includes question domains for pain, stiffness and functional status and patients with osteoarthritis were involved in the development of the questions. The WOMAC is incorporated in its entirety in the KOOS (see above). One study has evaluated the Dutch version of WOMAC in patients with meniscal tears.[32] In these patients, there is moderate positive evidence for

For peer review only

reliability and construct validity (hypothesis testing). No floor or ceiling effects were detected. Content validity and all other measurement properties are either indeterminate or were not reported (Table 6).

Health-related quality of life

EQ-5D: EQ-5D is a generic measure of health-related quality of life developed in 1990.[38] It was developed with patient involvement and includes question domains on mobility, self care, usual activities, pain, and anxiety or depression. One study has evaluated the English EQ-5D in patients with meniscal tears.[29] In this population, there is moderate positive evidence for construct validity based on hypothesis testing. All other measurement properties are either indeterminate or were not reported.

KQoL-26: The Knee Quality of Life (KQoL-26) 26-item questionnaire was developed in 2008, in English, as a disease-specific health-related quality of life measure for patients with suspected ligamentous or meniscal injury of the knee.[39] In the study population, 67% of patients had a meniscal tear and there is limited positive evidence for internal consistency, reliability, content validity, and construct validity (hypothesis testing and structural validity). Administered by post, an overall response rate of 59% was reported with 14.9% missing items.[39] Floor and ceiling effects were poorly reported with at least one question having an unacceptable floor effect and one an unacceptable ceiling effect (Table 5).

SF-6D: The short form-6 dimensions (SF-6D) generic health-related quality of life measure is derived from the SF-36 or SF-12 and was developed in 2004.[40] It was developed with patient involvement and contains 6 questions domains: physical functioning, role limitation, social functioning, pain, mental health and vitality. One study has evaluated the English version of SF-6D in patients with meniscal tears.[29] There is moderate positive evidence for construct validity based on hypothesis testing but all other measurement properties are indeterminate or were not reported.

WOMET: The Western Ontario Meniscal Evaluation Tool (WOMET) is a meniscal tear disease-specific, quality of life measure developed in 2007.[41] Patients with meniscal tears were involved throughout the development process although the authors reported that the same patients were "admittedly heterogeneous with respect to the incidence of coexisting knee pathology such as chondral damage or ligament injury".[41] The WOMET has been evaluated in English, Chinese, Dutch, Finnish and Turkish. There is strong positive evidence for content validity in the English version and moderate positive evidence in the Dutch version. There is limited positive evidence for reliability, construct validity (hypothesis testing) and responsiveness of the English-version.[41] Measurement error was only reported for the Dutch-version of WOMET and in this case it was concerning that the minimal important change (MIC) for the PROM was found to be less than the smallest detectable change (SDC). A summary of the level of evidence for the measurement properties in all languages is shown in Table 6. Although the overall score does not exhibit floor or ceiling effects, unacceptable levels were reported for several items (Table 5).

For peer review only

Activity Level

Tegner: The Tegner Activity Scale was developed in 1985 for patients with ACL injury.[36] Patients were not involved in the development of the scale. One study has evaluated use of the scale in patients with meniscal tears.[21] In this population, there is limited positive evidence for reliability and construct validity based on hypothesis testing. All other measurement properties were either not reported or indeterminate.

For peer review only

Discussion

This review identified 11 studies evaluating 10 PROMs in patients with meniscal tears: five PROMS measuring symptoms and functional status, four PROMs measuring health-related quality of life and one for activity level. Unfortunately, the findings of the studies were limited by poor methodology and incomplete reporting of measurement properties.

One previous review has been published summarising reported measurement properties of a range of PROMs in studies of patients with any knee condition.[42] In this previous review, WOMET was broadly recommended for use in patients with meniscal injuries without distinguishing the intended health-related quality of life construct from others or assessing the quality of the studies.[42] Ours is the first systematic review of PROMs for patients with meniscal tears and the first to evaluate and report the quality of study methodology. In orthopaedics and sports medicine, systematic reviews of PROMs applying the COSMIN appraisal checklist are established and have been published for patient populations including those with hip and knee osteoarthritis, hip and groin disability, patellofemoral pain, distal radius fractures, shoulder pain, and undergoing hip arthroscopy.[43–50]

For studies included in this review, the COSMIN methodology rating was poor for just over half (53%) of reported measurement properties. Internal consistency was rated poor in all but one of the 11 studies. A key reason for this was the failure of most studies to perform factor analysis to assess the structural validity of PROMs. Internal consistency is an assessment of the inter-relatedness of the items measuring the same underlying construct i.e. the PROM or sub-domain should be 'unidimensional' for the construct to be measured. Factor analysis is a technique that may be used determine whether a PROM or sub-domain is 'unidimensional'. Without this assessment of structural validity, there can be no clear interpretation of internal consistency statistics.[20]

Cross-cultural validity and responsiveness were also particularly poorly evaluated. Regarding responsiveness, frequently studies reported only an effect size for the studied PROM. Effect size alone is a measure of the magnitude of a change score and not the quality of the measurement and is therefore insufficient to assess this measurement property.[20] Responsiveness refers to the validity of a change score and should be assessed with, for example, hypothesis testing against the change score of another related PROM, analogous to the assessment of construct validity.

Measurement error was poorly reported in the included studies and the minimal important change (MIC) was calculated for only one of the PROMS – the Dutch-version of WOMET.[51] It was concerning that in this case the MIC was found to be less than the smallest detectable change (SDC) due to measurement error. Failure to determine and report this information affects the ability of researchers to design high-quality prospective studies and limits interpretation of previous work.

For peer review only - http://bmjopen.bmj.com/site/about/guidelines.xhtml

Evidence for the content validity of the available PROMs was limited. Only the KQoL-26 and WOMET were developed with involvement from patients with meniscal tears. Overall, there was heterogeneity in the population of the patients recruited to the included studies as shown in Table 2. Although most patients in the included studies had meniscal tears as their primary diagnosis, many also had a diagnosis of ligament injury or chondral damage. This reflects the heterogeneity of patients with meniscal tears in general, ranging from the isolated traumatic tear in a young athlete without osteoarthritis to atraumatic tears in older patients with osteoarthritis. Meniscal tears are not always symptomatic, and given the association with osteoarthritis, the distinction between the onset of meniscal pain and osteoarthritic pain is often unclear.[3,52] No single patient factor is sufficient in isolation.[5] For example, the degenerative meniscus will be more susceptible to tearing following knee trauma than a normal meniscus and no difference in symptom profile or treatment response has been demonstrated based on the mechanism of symptom onset.[53,54]

For studies in this review, there was significant variation in the methods used to identify patients. The latest guidance states specific types of meniscal tears should be identified on MRI imaging and related to symptoms and other findings before any surgical intervention is recommended.[5] Several studies included only patients with meniscal tears verified by previous arthroscopic surgery whereas others verified meniscal tears were visible on MRI imaging. For all the included studies, it was unclear how patients were identified to have symptoms correlating with a meniscal tear rather than other pathology such as osteoarthritis. Identifying patients with symptoms that definitely originate from the meniscus is challenging for both clinicians and researchers.

The mean age range of patients included in the studies was 38-53 years and therefore the generalisability of the findings to other age groups is unclear. It is highly likely that the symptom profile and expectations of younger, active patients sustaining a tear to a normal meniscus in an otherwise normal knee will be different to the study patients with predominantly degenerative meniscal tears and underlying osteoarthritis. This has not yet been evaluated.

<u>Strengths and limitations</u>

One strength of this review is the use of a validated, highly-sensitive search strategy to identify relevant studies.[23] A limitation, however, is that only studies specifically designed to appraise the measurement properties of PROMs were included. Trials and other clinical studies of patients with meniscal tears were not included as these studies are not designed to assess measurement properties and the reporting of these properties would be highly unusual. For the same reason, clinical trial registries were not searched for ongoing studies.

This is the first review of PROMs for patients with meniscal tears and the first to apply the COSMIN checklist, which is a validated and accepted tool for the appraisal of study quality. Although it has been shown to have acceptable inter-rater and intra-rater properties, the scoring of some items is reliant on author judgement.[55] We performed pre-testing to ensure scoring consistency and review authors scored studies independently with any disagreement being

For peer review only

settled by consensus or discussion with a third author. Nevertheless, it is feasible that another review team might score some items differently.

For practical purposes, we chose to include tentative summary guidance regarding the selection of PROMs for use in the target population. It should be understood, however, that it would be reasonable to declare that the overall level of evidence for any of the PROMs is insufficient for a recommendation to be made. Due to the study population limitations discussed earlier, the generalisability of the summary findings may also be challenged.

Implications for practice

Currently, although a wide range of PROMs are available for patients with knee conditions, the PROMs that have been tested in patients with meniscal tears all lack data on a large proportion of measurement properties. This is disappointing given moves to select condition-specific, standardised, 'core' outcome sets for use in clinical trials and general clinical evaluation.[13] Considerable further work is required before this will be possible for patients with meniscal tears.

For the assessment of symptoms and functional status in patients with meniscal tears, there is currently only very limited evidence supporting the selection of the English-version of Lysholm or IKDC, or Dutch-version of KOOS. Although the total score of these three PROMs does not exhibit floor or ceiling effects, a considerable number of sub-domain items from both IKDC and Lysholm were reported to have unacceptable floor or ceiling effects. For health-related quality of life, only limited evidence supports the selection of WOMET. One study suggests that measurement error may limit the ability of the WOMET to detect the minimal important change in score for meniscal patients.[51] Several WOMET sub-domain items, but not the total score, have been reported to exhibit unacceptable floor or ceiling effects. For assessment of activity level, only the Tegner activity scale has been evaluated and only very limited evidence is available.

Of all the PROMs evaluated, WOMET has the strongest evidence for content validity. In common with many of the validation studies in this population, however, the included patients frequently had other diagnoses in the same knee such as ligament injuries or chondral defects. This impacts upon the interpretation of clinical evidence in sub-groups of patients that were poorly represented within the development or validation study population. The findings of these validation studies may not be generalisable to such sub-groups and a PROM may fail to detect important clinical differences. Further validation studies may be required in sub-groups or the development of a more specific outcome measure may be necessary.[56] This is pertinent, for example, to current debate about the effectiveness of arthroscopic partial meniscectomy where there is an increasing focus on certain sub-groups of patients within this highly heterogeneous population.[12,54,57,58]

For peer review only - http://bmjopen.bmj.com/site/about/guidelines.xhtml

Conclusion

Many PROMs have been used in clinical studies of patients with meniscal tears but the overall quality of evidence supporting the validity of these PROMs is poor. Further work is required, targeting the deficiencies highlighted by this systematic review, to ensure these PROMs truly reflect the symptoms, function, and quality of life of patients with meniscal tears. This is necessary to inform the design and interpretation of clinical studies of interventions such as arthroscopic partial meniscectomy in patients with meniscal tears.

For peer review only

Statements

Contributorship statement

S. Abram: methodology, study selection, analysis, writing and editing paper.
R. Middleton: study selection, analysis, editing paper.
D.J. Beard: concept, editing paper.
A. J. Price: concept, editing paper.
S. Hopewell: methodology, analysis, editing paper.

Competing interests

No competing interests.

Funding

This report is independent research supported by the National Institute for Health Research (NIHR Doctoral Research Fellowship, Mr Simon Abram, DRF-2017-10-030). The views expressed in this publication are those of the authors and not necessarily those of the NHS, the National Institute for Health Research or the Department of Health.

Data sharing statement

No additional data available.

For peer review only

References

1 Stephen JM, Halewood C, Kittl C, Bollen SR, Williams A, Amis AA. Posteromedial Meniscocapsular Lesions Increase Tibiofemoral Joint Laxity With Anterior Cruciate Ligament Deficiency, and Their Repair Reduces Laxity. *Am J Sports Med* 2016; **44**: 400–8.

2 Rao AJ, Erickson BJ, Cvetanovich GL, Yanke AB, Bach BR, Cole BJ. The Meniscus-Deficient Knee: Biomechanics, Evaluation, and Treatment Options. *Orthop J Sports Med* 2015; **3**: 2325967115611386–2325967115611386.

3 Englund M, Guermazi A, Gale D, *et al.* Incidental meniscal findings on knee MRI in middle-aged and elderly persons. *N Engl J Med* 2008; **359**: 1108–15.

4 Roos EM, Roos HP, Ryd L, Lohmander LS. Substantial disability 3 months after arthroscopic partial meniscectomy: A prospective study of patient-relevant outcomes. *Arthroscopy* 2000; **16**: 619–26.

5 Stone JA, Salzler MJ, Parker DA, Becker R, Harner CD. Degenerative meniscus tears - assimilation of evidence and consensus statements across three continents: state of the art. *J ISAKOS Jt Disord Orthop Sports Med* 2017; **2**: 108–19.

6 Järvinen TLN, Guyatt GH. Arthroscopic surgery for knee pain. *Bmj* 2016; **3934**: i3934–i3934.

7 Sihvonen R, Paavola M, Malmivaara A, *et al.* Arthroscopic partial meniscectomy versus sham surgery for a degenerative meniscal tear. *N Engl J Med* 2013; **369**: 2515–24.

8 Katz JN, Brophy RH, Chaisson CE, *et al.* Surgery versus physical therapy for a meniscal tear and osteoarthritis. *N Engl J Med* 2013; **368**: 1675–84.

9 Herrlin SV, Wange PO, Lapidus G, Hållander M, Werner S, Weidenhielm L. Is arthroscopic surgery beneficial in treating non-traumatic, degenerative medial meniscal tears? A five year follow-up. *Knee Surg Sports Traumatol Arthrosc* 2013; **21**: 358–64.

10 Yim J-H, Seon J-K, Song E-K, *et al.* A comparative study of meniscectomy and nonoperative treatment for degenerative horizontal tears of the medial meniscus. *Am J Sports Med* 2013; **41**: 1565–70.

11 Kise NJ, Risberg MA, Stensrud S, Ranstam J, Engebretsen L, Roos EM. Exercise therapy versus arthroscopic partial meniscectomy for degenerative meniscal tear in middle aged patients: randomised controlled trial with two year follow-up. *BMJ* 2016; **354**. DOI:10.1136/bmj.i3740.

12 Monk P, Garfjeld Roberts P, Palmer AJR, *et al.* The Urgent Need for Evidence in Arthroscopic Meniscal Surgery: A Systematic Review of the Evidence for Operative Management of Meniscal Tears. *Am J Sports Med* 2016. DOI:10.1177/0363546516650180.

13 Williamson PR, Altman DG, Blazeby JM, *et al.* Developing core outcome sets for clinical trials: issues to consider. *Trials* 2012; **13**: 132–132.

14 van de Graaf VA, Scholtes VAB, Wolterbeek N, *et al.* Cost-effectiveness of Early Surgery versus Conservative Treatment with Optional Delayed Meniscectomy for Patients over 45 years with non-obstructive meniscal tears (ESCAPE study): protocol of a randomised controlled trial. *BMJ Open* 2016; **6**: e014381–e014381.

15 Brazier JE, Harper R, Munro J, Walters SJ, Snaith ML. Generic and condition-specific outcome measures for people with osteoarthritis of the knee. *Rheumatology* 1999; **38**: 870–7.

For peer review only

16 Guyatt GH, Bombardier C, Tugwell PX. Measuring disease-specific quality of life in clinical trials. *CMAJ Can Med Assoc J J Assoc Medicale Can* 1986; **134**: 889–95.

17 Mokkink LB, Terwee CB, Stratford PW, *et al.* Evaluation of the methodological quality of systematic reviews of health status measurement instruments. *Qual Life Res* 2009; **18**: 313–33.

18 Moher D, Liberati A, Tetzlaff J, Altman DG. Preferred reporting items for systematic reviews and meta-analyses: the PRISMA statement. *BMJ* 2009; **339**. DOI:10.1136/bmj.b2535.

19 Mokkink LB, Terwee CB, Patrick DL, *et al.* The COSMIN study reached international consensus on taxonomy, terminology, and definitions of measurement properties for health-related patient-reported outcomes. *J Clin Epidemiol* 2010; **63**: 737–45.

20 Mokkink LB, Terwee CB, Knol DL, *et al.* The COSMIN checklist for evaluating the methodological quality of studies on measurement properties: a clarification of its content. *BMC Med Res Methodol* 2010; **10**: 22–22.

21 Briggs KK, Kocher MS, Rodkey WG, Steadman JR. Reliability, validity, and responsiveness of the Lysholm knee score and Tegner activity scale for patients with meniscal injury of the knee. *J Bone Joint Surg Am* 2006; **88**: 698–705.

22 Terwee CB, Bot SDM, de Boer MR, *et al.* Quality criteria were proposed for measurement properties of health status questionnaires. *J Clin Epidemiol* 2007; **60**: 34–42.

23 Terwee CB, Jansma EP, Riphagen II, De Vet HCW. Development of a methodological PubMed search filter for finding studies on measurement properties of measurement instruments. *Qual Life Res* 2009; **18**: 1115–23.

24 Terwee CB, Mokkink LB, Knol DL, Ostelo RWJG, Bouter LM, De Vet HCW. Rating the methodological quality in systematic reviews of studies on measurement properties: A scoring system for the COSMIN checklist. *Qual Life Res* 2012; **21**: 651–7.

25 Mokkink LB, Terwee CB, Patrick DL, *et al.* The COSMIN checklist for assessing the methodological quality of studies on measurement properties of health status measurement instruments: An international Delphi study. *Qual Life Res* 2010; **19**: 539–49.

26 Schellingerhout JM, Verhagen AP, Heymans MW, Koes BW, De Vet HC, Terwee CB. Measurement properties of disease-specific questionnaires in patients with neck pain: A systematic review. *Qual Life Res* 2012; **21**: 659–70.

27 Van Tulder M, Furlan A, Bombardier C, Bouter L. Updated Method Guidelines for Systematic Reviews in the Cochrane Collaboration Back Review Group. *Spine* 2003; **28**: 1290–9.

28 Flandry F, Hunt JP, Terry GC, Hughston JC. Analysis of subjective knee complaints using visual analog scales. *Am J Sports Med* 1991; **19**: 112–8.

29 Goodwin PC, Ratcliffe J, Morris J, Morrissey MC. Using the knee-specific Hughston Clinic Questionnaire, EQ-5D and SF-6D following arthroscopic partial meniscectomy surgery: A comparison of psychometric properties. *Qual Life Res* 2011; **20**: 1437–46.

30 Irrgang JJ, Anderson AF, Boland AL, *et al.* Development and validation of the international knee documentation committee subjective knee form. *Am J Sports Med* 2001; **29**: 600–13.

31 Crawford K, Briggs KK, Rodkey WG, Steadman JR. Reliability, Validity, and Responsiveness of the IKDC Score for Meniscus Injuries of the Knee. *Arthrosc - J Arthrosc Relat Surg* 2007; **23**: 839–44.

For peer review only

32 van de Graaf VA, Wolterbeek N, Scholtes VA, Mutsaerts EL, Poolman RW. Reliability and Validity of the IKDC, KOOS, and WOMAC for Patients With Meniscal Injuries. *Am J Sports Med* 2014; **42**: 1408–16.

33 Roos EM, Roos HP, Lohmander LS, Ekdahl C, Beynnon BD. Knee Injury and Osteoarthritis Outcome Score (KOOS)—Development of a Self-Administered Outcome Measure. *J Orthop Sports Phys Ther* 1998; **28**: 88–96.

34 Roos EM, Roos HP, Ekdahl C, Lohmander LS. Knee injury and Osteoarthritis Outcome Score (KOOS)--validation of a Swedish version. *Scand J Med Sci Sports* 1998; **8**: 439–48.

35 Lysholm J, Gillquist J, Anonymous. Evaluation of Knee Ligament Surgery Results with Special Emphasis on Use of a Scoring Scale. *Am J Sports Med* 1982; **10**: 150–4.

36 Tegner Y, Lysholm J. Rating systems in the evaluation of knee ligament injuries. *Clin Orthop* 1985; : 43–9.

37 Bellamy N. The WOMAC Knee and Hip Osteoarthritis Indices: Development, validation, globalization and influence on the development of the AUSCAN Hand Osteoarthritis Indices. *Clin Exp Rheumatol* 2005; **23**. DOI:10.1097/01.prs.0000436796.74305.38.

38 The EuroQol Group. EuroQol - a new facility for the measurement of health-related quality of life. *Health Policy* 1990; **16**: 199–208.

39 Garratt AM, Brealey S, Robling M, *et al.* Development of the Knee Quality of Life (KQoL-26) 26-item questionnaire: data quality, reliability, validity and responsiveness. *Health Qual Life Outcomes* 2008; **6**: 48.

40 Braizer J, Roberts J, Deverill M. The Estimation of a Preference-Based Measure of Health Form the SF-36. *Journall Health Econ* 2002; **21**: 271–92.

41 Kirkley A, Griffin S, Whelan D. The development and validation of a quality of life-measurement tool for patients with meniscal pathology: the Western Ontario Meniscal Evaluation Tool (WOMET). *Clin J Sport Med Off J Can Acad Sport Med* 2007; **17**: 349–56.

42 Wang D, Jones H, Khair M, Miniaci A. Patient-reported outcome measures for the knee. *J Knee Surg* 2010; **23**: 137–51.

43 Thorborg K, Tijssen M, Habets B, *et al.* Patient-Reported Outcome (PRO) questionnaires for young to middle-aged adults with hip and groin disability: a systematic review of the clinimetric evidence. *Br J Sports Med* 2015; **49**: 812–812.

44 Thoomes-de Graaf M, Scholten-Peeters GGM, Schellingerhout JM, *et al.* Evaluation of measurement properties of self-administered PROMs aimed at patients with non-specific shoulder pain and activity limitations: a systematic review. *Qual Life Res* 2016; **25**: 2141–60.

45 Green A, Liles C, Rushton A, Kyte DG. Measurement properties of patient-reported outcome measures (PROMS) in Patellofemoral Pain Syndrome: A systematic review. *Man Ther* 2014; **19**: 517–26.

46 Terwee CB, Bouwmeester W, van Elsland SL, de Vet HCW, Dekker J. Instruments to assess physical activity in patients with osteoarthritis of the hip or knee: A systematic review of measurement properties. *Osteoarthritis Cartilage* 2011; **19**: 620–33.

For peer review only

47 Gagnier JJ, Mullins M, Huang H, *et al.* A Systematic Review of Measurement Properties of Patient-Reported Outcome Measures Used in Patients Undergoing Total Knee Arthroplasty. *J Arthroplasty* 2017. DOI:10.1016/j.arth.2016.12.052.

48 Kroman SL, Roos EM, Bennell KL, Hinman RS, Dobson F. Measurement properties of performance-based outcome measures to assess physical function in young and middle-aged people known to be at high risk of hip and/or knee osteoarthritis: A systematic review. *Osteoarthritis Cartilage* 2014; **22**: 26–39.

49 Dobson F, Hinman RS, Hall M, Terwee CB, Roos EM, Bennell KL. Measurement properties of performance-based measures to assess physical function in hip and knee osteoarthritis: A systematic review. *Osteoarthritis Cartilage* 2012; **20**: 1548–62.

50 Tijssen M, van Cingel R, van Melick N, de Visser E. Patient-Reported Outcome questionnaires for hip arthroscopy: a systematic review of the psychometric evidence. *BMC Musculoskelet Disord* 2011; **12**: 117–117.

51 van der Wal RJP, Heemskerk BTJ, van Arkel ERA, Mokkink LB, Thomassen BJW. Translation and Validation of the Dutch Western Ontario Meniscal Evaluation Tool. *J Knee Surg* 2017; **30**: 314–22.

52 Guermazi A, Niu J, Hayashi D, *et al.* Prevalence of abnormalities in knees detected by MRI in adults without knee osteoarthritis: population based observational study (Framingham Osteoarthritis Study). *BMJ* 2012; **345**: e5339–e5339.

53 Haviv B, Bronak S, Kosashvili Y, Thein R. Arthroscopic meniscectomy of traumatic versus atraumatic tears in middle aged patients: is there a difference? *Arch Orthop Trauma Surg* 2016; **136**: 1297–301.

54 Thorlund JB, Englund M, Christensen R, *et al.* Patient reported outcomes in patients undergoing arthroscopic partial meniscectomy for traumatic or degenerative meniscal tears: comparative prospective cohort study. *Bmj* 2017; **356**: j356–j356.

55 Mokkink LB, Terwee CB, Gibbons E, *et al.* Inter-rater agreement and reliability of the COSMIN (COnsensus-based Standards for the selection of health status Measurement Instruments) Checklist. *Med Res Methodol* 2010; **10**: 1–11.

56 McKenna SP. Measuring patient-reported outcomes: moving beyond misplaced common sense to hard science. *BMC Med* 2011; **9**: 86–86.

57 Tornbjerg SM, Nissen N, Englund M, *et al.* Structural pathology is not related to patient-reported pain and function in patients undergoing meniscal surgery. *Br J Sports Med* 2016; : bjsports-2016-096456.

58 Thorlund JB, Juhl CB, Roos EM, Lohmander LS. Arthroscopic surgery for degenerative knee: systematic review and meta-analysis of benefits and harms. *Bmj* 2015; **350**: h2747–h2747.

59 Sihvonen R, Järvelä T, Aho H, Järvinen TLN. Validation of the Western Ontario Meniscal Evaluation Tool (WOMET) for patients with a degenerative meniscal tear: a meniscal pathology-specific quality-of-life index. *J Bone Joint Surg Am* 2012; **65**: 8–15.

60 Celik D, Demirel M, Kuş G, Erdil M, Özdinçler AR. Translation, cross-cultural adaptation, reliability and validity of the Turkish version of the Western Ontario Meniscal Evaluation Tool (WOMET). *Knee Surg Sports Traumatol Arthrosc* 2015; **23**: 816–25.

61 Tong WW, Wang W, Xu WD. Development of a Chinese version of the Western Ontario Meniscal Evaluation Tool: cross-cultural adaptation and psychometric evaluation. *J Orthop Surg* 2016; **11**. DOI:10.1186/s13018-016-0424-8.

For peer review only

62 Bellamy N, Buchanan WW, Goldsmith CH, Campbell J, Stitt LW. Validation study of WOMAC: a health status instrument for measuring clinically important patient relevant outcomes to antirheumatic drug therapy in patients with osteoarthritis of the hip or knee. *J Rheumatol* 1988; **15**: 1833–40.

For peer review only

Tables

Table 1: Overall levels of evidence for the quality of the measurement property[26,27]

The quality of the evidence for the measurement property for each PROM, considering the quality criteria for each measurement property (Appendix 2), the methodology of each study reporting the measurement property (Table 4) and the number of studies reporting the measurement property including consistency of findings.

Level of Evidence	Rating	Quality Criteria
Strong	+++<br>or<br>- - -	Consistent findings (positive or negative) in multiple studies of good methodological quality OR in one study of excellent methodological quality
Moderate	++<br>or<br>- -	Consistent findings (positive or negative) in multiple studies of fair methodological quality OR in one study of good methodological quality
Limited	+<br>or<br>-	One study of fair methodological quality (positive or negative)
Conflicting	+/-	Conflicting results
Unknown	?	Only studies of poor methodological quality

+ = positive rating, ? = indeterminate rating, - = negative rating

For peer review only

Table 2: Characteristics of the included studies

Study (year)	Instrument(s)	Country (language)	Population (inclusion and exclusion criteria)	*N*	Mean age (SD, range)	Female : Male
Goodwin (2011)[29]	**Hughston EQ-5D SF-6D**	**UK (English)**	**Inclusion:** Patients previously undergoing arthroscopic partial meniscectomy.	84	38 (SD 8, 21-58)	14 : 86 %
Crawford (2007)[31]	**IKDC**	**USA (English)**	**Inclusion:** Patient with "meniscal pathology requiring treatment" and completed IKDC questionnaire **Exclusion:** Patients with ligament pathology or a chondral defect greater than Outerbridge grade 2	Groups: A: 31 B: 264 C: 50 D: 50	48 (18-81)	29 : 71 %
Van de Graaf (2014)[32]	**IKDC KOOS WOMAC**	**Netherlands (Dutch)**	**Inclusion:** Age >18, knowledge of Dutch language, either on waiting list for meniscal surgery or between 6 weeks and 6 months after meniscal surgery. **Exclusion:** Received arthroplasty in either knee or previous ACL surgery on the knee of interest.	75	48.8 (35-62)	50 : 50 %
Roos (1998)[34]	**KOOS**	**Sweden (Swedish)**	**Inclusion:** Patient waiting for knee arthroscopy for either meniscal lesion, ACL injury or tibio-femoral cartilage damage. (54% meniscal tear, 20% ACL+meniscal tear, 13% ACL, 13% isolated chondral damage) **Exclusion:** Multiple joint involvement, other diagnosis.	142	39.7 (14-75)	22 : 78 %
Garratt (2008)[39]	**KQoL-26**	**UK (English)**	**Inclusion:** Patients aged 18-55, referred to hospital clinic with suspected meniscus or knee ligament pathology. (67% meniscal tear, 30% ACL, 3% other) **Exclusion:** Requiring urgent referral, non-traumatic arthropathy, chronic knee instability, previous same knee surgery (except diagnostic arthroscopy)	323	47 (14.3)	44 : 56 %
Briggs (2006)[21]	**Lysholm Tegner**	**USA (English)**	**Inclusion:** Patient previously undergoing surgery for meniscal lesion or waiting list for meniscal surgery.	Groups: A: 122 B: 191 C: 477	40 (13-81)	32 : 68 %
Kirkley (2007)[41]	**WOMET**	**Canada (English)**	**Inclusion:** Patients with "meniscal symptoms (swelling, catching, locking)" and magnetic resonance imaging suggestive of meniscal pathology.	Groups: A: 31 B: 36 C: 34 D: 69	Not reported	Not reported
Sihvonen (2012)[59]	**WOMET**	**Finland (Finnish)**	**Inclusion:** Patients with arthroscopically verified degenerative meniscal tear and no previous knee trauma. **Exclusion:** Trauma, bilateral arthroscopy, re-operation within 6 months	Groups: A: 485 B: 385 C: 100 D: 40	53 (18-81)	45 : 55 %
Celik (2013)[60]	**WOMET**	**Turkey (Turkish)**	**Inclusion:** Age >16, presence of meniscal tear or previous meniscal repair or resection, complete questionnaires. **Exclusion:** Ligament injury, "articular cartilage damage causing instability", inability to complete the form due to cognitive impairment.	96	43.6 (23-71)	64 : 36 %
Tong (2016)[61]	**WOMET**	**China (Chinese)**	**Inclusion:** Patients with meniscal pathology who underwent arthroscopic surgery for meniscal repair or resection. Age >18, able to read and speak Chinese. **Exclusion:** Ligament injuries, history of leg surgery, infection, tumours, rheumatologic disease, neurological or musculoskeletal disorders.	121	41.2 (14.3)	57 : 43 %
Van der Wal (2016)[51]	**WOMET**	**Netherlands (Dutch)**	**Inclusion:** Patients with MRI confirmed, symptomatic, meniscal tear. Age 18-70, understanding of Dutch language. **Exclusion:** Concomitant ligament injury, previous ligament injury with instability, previous knee surgery, chondral defect greater than Outerbridge grade 2 on MRI or during surgery, inability to participate due to cognitive impairment.	86	Median 52 (IQr 43-60)	41 : 59 %

IQr = interquartile range.

For peer review only

Table 3: Characteristics of the included PROMs

Instrument	Year of development	Original language	Intended Construct & Domains	Number of questions	Target or Development Population	Patients involved in development?
**Symptoms & Functional Status**						
Hughston	1991[29]	English	**Knee-specific symptoms, functional status, sports activity.**<br>No sub-domains	28 questions	"Patients who had undergone knee surgery that varied from arthroscopy to total arthroplasty."[28]	No
IKDC	2001[30]	English	**Knee-specific symptoms, functional status, sports activity.**<br>1. Symptoms<br>2. Sports activities<br>3. Function	18 questions	"A knee-specific, rather than a disease-specific, measure of symptoms, function, and sports activity."[30]	No
KOOS	1998[33]	English	**Knee injury-specific symptoms, functional status, sports activity, quality of life.**<br>1. Symptoms & Stiffness<br>2. Pain<br>3. Activities of daily living (ADL)<br>4. Function in sports and recreation<br>5. Knee-related quality of life (QoL)	42 questions	Patients with knee injury (ACL or meniscus injury) at risk of developing osteoarthritis.	Yes
Lysholm	1982[35] / 1985[36]	English	**Disease-specific (knee ligament) symptoms, functional status**<br>No sub-domains	8 questions	"A scoring scale for knee ligament surgery follow-up emphasizing evaluation of symptoms of instability."[35]	No
WOMAC	1982[37]	English	**Disease-specific (osteoarthritis of hip or knee) symptoms, functional status**<br>1. Pain<br>2. Stiffness<br>3. Function & Daily activities	24 questions	"Outcomes of anti-rheumatic drug therapy in patients with osteoarthritis of the hip or knee."[62]	Yes
**Health-related quality of life**						
EQ-5D	1990[38]	English, Dutch, Finnish, Norwegian, Swedish	**General population health-related quality of life**<br>1. Mobility<br>2. Self-care<br>3. Usual activities<br>4. Pain/Discomfort<br>5. Anxiety/Depression	6 questions	General tool for describing and valuing health related quality of life – items developed and valued after questioning large samples of randomly selected adults.	Yes
KQoL-26	2008[39]	English	**Disease-specific (knee ligament or meniscus) health-related quality of life**<br>1. Physical functioning<br>2. Activity limitations<br>3. Emotional functioning	26 questions	"Patients with a suspected ligamentous or meniscal injury of the knee."[39]	Yes
SF-6D	2004[40]	English	**General population health-related quality of life**<br>1. Physical functioning<br>2. Role limitation<br>3. Social functioning<br>4. Pain<br>5. Mental health<br>6. Vitality	6 questions	Derived from SF-36 or SF-12. A general, preference based classification for describing health-related quality of life.	Yes
WOMET	2007[41]	English	**Disease-specific (meniscus) health-related quality of life**<br>Physical symptoms<br>1. Sports/Recreation/Work/Lifestyle<br>2. Emotions	16 questions	"Patients with meniscal symptomology (swelling, catching, locking) and in whom magnetic resonance imaging had suggested meniscal pathology."[41]	Yes
**Activity level**						
Tegner	1985[36]	English	**Disease-specific (knee ligament) symptoms, functional status**<br>No sub-domains	1 question	Patients with ACL injury diagnosed by clinical examination under anaesthesia and confirmed by arthroscopy or arthrotomy.	No

ACL = anterior cruciate ligament

For peer review only

Table 4: Quality of each study per PROM and measurement property (COSMIN rating)

**Instrument and Study**	Internal consistency	Reliability	Measurement error	Content validity	Structural validity	Hypothesis testing	Cross-cultural validity	Responsiveness
Symptoms & Functional Status								
**Hughston**								
Goodwin (2011)[29]	Poor	na	na	Poor	na	Good	na	Poor
**IKDC**								
Crawford (2007)[31]	Poor	Fair	Fair	Poor	na	Fair	na	Poor
Van de Graaf (2014)[32]	Poor	Good	Good	Poor	Fair	Good	Poor	na
**KOOS**								
Roos (1998)[34]	Poor	Fair	na	Poor	Poor	Fair	Poor	Poor
Van de Graaf (2014)[32]	Poor	Good	Good	Poor	Poor	Good	Poor	na
**Lysholm**								
Briggs (2006)[21]	Poor	Fair	Fair	Poor	na	Fair	na	Poor
**WOMAC**								
Van de Graaf (2014)[32]	Poor	Good	Good	Poor	Poor	Good	Poor	na
Health-related quality of life								
**EQ-5D**								
Goodwin (2011)[29]	Poor	na	na	Poor	na	Good	na	Poor
**KQoL-26**								
Garratt (2008)[39]	Fair	Fair	na	Fair	Fair	Fair	na	Poor
**SF-6D**								
Goodwin (2011)[29]	Poor	na	na	Poor	na	Good	na	Poor
**WOMET**								
Kirkley (2007)[41]	Poor	Fair	na	Excellent	na	Fair	na	Fair
Sihvonen (2012)[59]	Poor	Poor	na	Poor	na	Fair	Poor	Poor
Celik (2013)[60]	Poor	Good	Good	Poor	na	Good	Poor	na
Tong (2016)[61]	Poor	Good	na	Poor	na	Good	Poor	Poor
Van der Wal (2016)[51]	Poor	Good	Good	Good	na	Good	Poor	Good
Activity level								
**Tegner**								
Briggs (2006)[21]	na	Fair	Fair	Poor	na	Fair	na	Poor

For peer review only

Table 5: Interpretability including missing items, response rate, floor and ceiling effects

Instrument and Study	Administration	Missing responses	Missing items	Overall % lowest possible total score (floor)	Overall % highest possible score (ceiling)	Items or Domains with >15% responses with lowest score (floor)	Items or Domains >15% highest possible score (ceiling)	MIC
**Symptoms & Functional Status**								
**Hughston**								
Goodwin (2011)[29]	Clinic	Not reported	Not reported	0%	0%	Not reported	Not reported	Not reported
**IKDC**								
Crawford (2007)[31]	Clinic / Postal	Not reported	Not reported	0%	0%	• Activity pain<br>• Pain last 4 weeks<br>• Pain severity<br>• Catching<br>• Kneeling<br>• Sitting<br>• Running<br>• Jumping<br>• Stopping	• Swelling<br>• Catching<br>• Climb stairs<br>• Sitting<br>• Rising	Not reported
Van de Graaf (2014)[32]	Online / Postal	Unclear	0%	0%	0%	Nil	Nil	Not reported
**KOOS**								
Roos (1998)[34]	Postal	7.2%	0.8%	Not reported	Not reported	Nil	Nil	Not reported
Van de Graaf (2014)[32]	Online / Postal	Unclear	0%	0%	3%	Nil	Nil	Not reported
**Lysholm**								
Briggs (2006)[21]	Clinic	Not reported	Not reported	0%	0.5%	• Squatting<br>• Pain	• Swelling<br>• Instability<br>• Support<br>• Limp<br>• Locking	Not reported
**WOMAC**								
Van de Graaf (2014)[32]	Online / Postal	Unclear	0%	0%	6%	Nil	Nil	Not reported
**Health-related quality of life**								
**EQ-5D**								
Goodwin (2011)[29]	Clinic	Not reported	Not reported	4%	1%	Not reported	Not reported	Not reported
**KQoL-26**								
Garratt (2008)[39]	Postal	41%	14.9%	Not reported	Not reported	• Avoiding turning, twisting, or sideways movements<br>• *	• Staying seated for 15 minutes<br>• *	Not reported
**SF-6D**								
Goodwin (2011)[29]	Clinic	Not reported	Not reported	0%	0%	Not reported	Not reported	Not reported
**WOMET**								
Kirkley (2007)[41]	Clinic	Not reported	Not reported	5.7%	1.7%	Not reported	Not reported	Not reported
Sihvonen (2012)[59]	Unclear	16%	7.5%	0%	0%	• Numbness	Nil	Not reported
Celik (2013)[60]	Unclear	Not reported	Not reported	0%	0%	• Numbness<br>• Swelling	• Consciousness<br>• Activities<br>• Specific skills<br>• Squatting<br>• Fear injury<br>• Concern about future of knee<br>• Frustration	Not reported
Tong (2016)[61]	Unclear	Not reported	0%	0%	0%	Nil	Nil	Not reported
Van der Wal (2016)[51]	Clinic	0%	<1%	0%	0%	• Numbness<br>• Swelling	Nil	14.7
**Activity level**								
**Tegner**								
Briggs (2006)[21]	Clinic	Not reported	Not reported	2.5%	2.5%	na	na	Not reported

MIC = minimal important change; * = Other domains not reported

For peer review only

Table 6: Overall rating of measurement properties and level of evidence for each PROM

See table 1 for a summary of the rating methodology.

Instrument	Internal consistency	Reliability	Measurement error	Content validity	Structural validity	Hypothesis testing	Cross-cultural validity	Responsiveness
**Symptoms & Functional Status**								
**Hughston**								
English[29]	?	na	na	?	na	- -	na	?
**IKDC**								
English[31]	?	+	?	?	na	+	na	?
Dutch[32]	?	++	?	?	-	++	?	na
**KOOS**								
Dutch[32]	?	++	?	?	?	++	?	na
Swedish[34]	?	+	na	?	?	+	?	?
**Lysholm**								
English[21]	?	+	?	?	na	+	na	?
**WOMAC**								
Dutch[32]	?	++	?	?	?	++	?	na
**Health-related quality of life**								
**EQ-5D**								
English[29]	?	na	na	?	na	++	na	?
**KQoL-26**								
English[39]	+	+	na	+	+	+	na	?
**SF-6D**								
English[29]	?	na	na	?	na	++	na	?
**WOMET**								
English[41]	?	+	na	+++	na	+	na	+
Chinese[61]	?	++	na	?	na	++	?	?
Dutch[51]	?	++	- -	++	na	++	?	++
Finnish[59]	?	?	na	?	na	+	?	?
Turkish[60]	?	++	?	?	na	++	?	na
**Activity level**								
**Tegner**								
English[21]	na	+	?	?	na	+	na	?

For peer review only

Figure legends

Figure 1: PRISMA Flow Diagram
Overview of study selection. Full search strategy may be found in supplementary appendix 1.

For peer review only

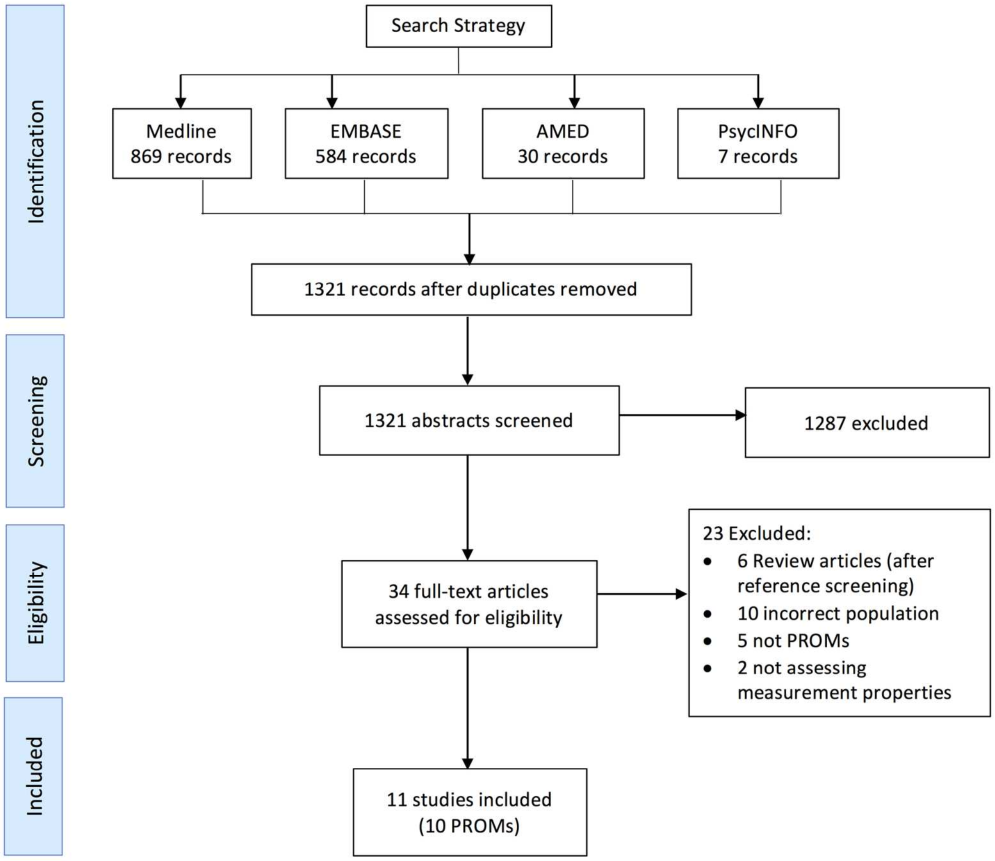

PRISMA Flow Diagram:
Overview of study selection. Full search strategy may be found in supplementary appendix 1.

159x139mm (300 x 300 DPI)

only

Appendix 1: Search strategy

Databases: MEDLINE, Embase, AMED, PsycInfo

#1 Condition

menis*.af

#2 Construct

("quality of life" OR qol OR func* OR HR-PRO OR HRPRO OR HRQOL OR QL OR disab* OR wellbeing OR "well being" OR subjective OR utility OR utilities OR priorit* OR outcome* OR health).af

#3 Instrument

(score* OR measure* OR PROM OR index* OR indices OR scale* OR questionnaire* OR instrument* OR survey* OR profile* OR apprais* OR status OR reported OR reporting OR rated OR rating* OR assessment*).af

#4 Measurement Properties

"Validation Studies".pt OR instrumentation.af OR ("observer variation" OR "psychometrics" OR "reproducibility of results" OR "discriminant analysis").mh OR (agreement OR precision OR imprecision OR "precise values" OR repeatab* OR ((replicab* OR repeated) AND (measure OR measures OR findings OR result OR results OR test OR tests))).af OR (reproducib* OR psychometr* OR clinimetr* OR clinometr* OR observer AND variation OR reliab* OR valid* OR coefficient OR "internal consistency" OR (cronbach* AND (alpha OR alphas)) OR "item correlation" OR "item correlations" OR "item selection" OR "item selections" OR "item reduction" OR "item reductions" OR test?retest OR (test AND retest) OR (reliab* AND (test OR retest)) OR stability OR interrater OR inter-rater OR intrarater OR intra-rater OR intertester OR inter-tester OR intratester OR intra-tester OR interobserver OR inter-observer OR intraobserver OR intra-observer OR intertechnician OR intertechnician OR intratechnician OR intra-technician OR interexaminer OR inter-examiner OR intraexaminer OR intra-examiner OR inter-assay OR inter-assay OR intraassay OR intra-assay OR interindividual OR inter-individual OR intraindividual OR intra-individual OR interparticipant OR inter-participant OR intraparticipant OR intra-participant OR kappa OR kappa?s OR "coefficient of variation" OR generaliza* OR generalisa* OR concordance OR (intraclass AND correlation*) OR discriminative OR "known group" OR "factor analysis" OR "factor analyses" OR "factor structure" OR "factor structures" OR dimensionality OR subscale* OR "multitrait scaling analysis" OR "multitrait scaling analyses" OR "item discriminant" OR "interscale correlation" OR "interscale correlations" OR ((error OR errors) AND (measure* OR correlat* OR evaluat* OR accuracy OR accurate OR precision OR mean)) OR "individual variability" OR "interval variability" OR "rate variability").ti,ab NOT ("addresses" OR "biography" OR "case reports" OR "comment" OR "directory" OR "editorial" OR "festschrift" OR "interview" OR "lectures" OR "legal cases" OR "legislation" OR "letter" OR "news" OR "newspaper article" OR "patient education handout" OR "popular works" OR "congresses" OR "consensus development conference" OR "consensus development conference, nih" OR "practice guideline").pt

#5 (#1 AND #2 AND #3 AND #4)

#5 Remove Duplicates

Appendix 2: Quality criteria for measurement properties[1]

Property	Rating	Quality Criteria
**Reliability**		
Internal consistency	+	Cronbach's alpha(s) ≥ 0.70
	?	Cronbach's alpha not determined or dimensionality unknown
	-	Cronbach's alpha(s) < 0.70
Reliability	+	ICC / weighted Kappa ≥ 0.70 OR Pearson's r ≥ 0.80
	?	Neither ICC / weighted Kappa, nor Pearson's r determined
	-	ICC / weighted Kappa < 0.70 OR Pearson's r < 0.80
Measurement error	+	MIC > SDC OR MIC outside the LOA
	?	MIC not defined
	-	MIC ≤ SDC OR MIC equals or inside LOA
**Validity**		
Content validity	+	All items are considered to be relevant for the construct to be measured, for the target population, and for the purpose of the measurement AND the questionnaire is considered to be comprehensive
	?	Not enough information available
	-	Not all items are considered to be relevant for the construct to be measured, for the target population, and for the purpose of the measurement OR the questionnaire is considered not to be comprehensive
Construct validity – Structural validity	+	Factors should explain at least 50% of the variance
	?	Explained variance not mentioned
	-	Factors explain < 50% of the variance
Construct validity – Hypothesis testing	+	Correlations with instruments measuring the same construct ≥ 0.50 OR at least 75% of the results are in accordance with the hypotheses AND correlations with related constructs are higher than with unrelated constructs
	?	Solely correlations determined with unrelated constructs
	-	Correlations with instruments measuring the same construct < 0.50 OR < 75% of the results are in accordance with the hypotheses OR correlations with related constructs are lower than with unrelated constructs
Construct validity – Cross-cultural validity	+	No differences in factor structure OR no important DIF between language versions
	?	Multiple group factor analysis not applied AND DIF not assessed
	-	Differences in factor structure OR important DIF between language versions
**Responsiveness**		
Responsiveness	+	Correlation with changes on instruments measuring the same construct ≥ 0.50 OR at least 75% of the results are in accordance with the hypotheses OR AUC ≥ 0.70 AND correlations with changes in related constructs are higher than with unrelated constructs
	?	Solely correlations determined with unrelated constructs
	-	Correlations with changes on instruments measuring the same construct < 0.50 OR < 75% of the results are in accordance with the hypotheses OR AUC < 0.70 OR correlations with changes in related constructs are lower than with unrelated constructs

MIC = minimal important change, SDC = smallest detectable change, LoA = limits of agreement, ICC = intraclass correlation coefficient, DIF = differential item functioning, AUC = area under the curve
+ = positive rating, ? = indeterminate rating, - = negative rating

1 Terwee CB, Bot SDM, de Boer MR, *et al.* Quality criteria were proposed for measurement properties of health status questionnaires. *J Clin Epidemiol* 2007; **60**: 34–42.

For peer review only - http://bmjopen.bmj.com/site/about/guidelines.xhtml

PRISMA 2009 Checklist

Section/topic	#	Checklist item	Reported on page #
**TITLE**			
Title	1	Identify the report as a systematic review, meta-analysis, or both.	1
**ABSTRACT**			
Structured summary	2	Provide a structured summary including, as applicable: background; objectives; data sources; study eligibility criteria, participants, and interventions; study appraisal and synthesis methods; results; limitations; conclusions and implications of key findings; systematic review registration number.	2-3
**INTRODUCTION**			
Rationale	3	Describe the rationale for the review in the context of what is already known.	3
Objectives	4	Provide an explicit statement of questions being addressed with reference to participants, interventions, comparisons, outcomes, and study design (PICOS).	3
**METHODS**			
Protocol and registration	5	Indicate if a review protocol exists, if and where it can be accessed (e.g., Web address), and, if available, provide registration information including registration number.	4
Eligibility criteria	6	Specify study characteristics (e.g., PICOS, length of follow-up) and report characteristics (e.g., years considered, language, publication status) used as criteria for eligibility, giving rationale.	4
Information sources	7	Describe all information sources (e.g., databases with dates of coverage, contact with study authors to identify additional studies) in the search and date last searched.	7
Search	8	Present full electronic search strategy for at least one database, including any limits used, such that it could be repeated.	7 + Appendix1
Study selection	9	State the process for selecting studies (i.e., screening, eligibility, included in systematic review, and, if applicable, included in the meta-analysis).	7
Data collection process	10	Describe method of data extraction from reports (e.g., piloted forms, independently, in duplicate) and any processes for obtaining and confirming data from investigators.	7
Data items	11	List and define all variables for which data were sought (e.g., PICOS, funding sources) and any assumptions and simplifications made.	4-7
Risk of bias in individual studies	12	Describe methods used for assessing risk of bias of individual studies (including specification of whether this was done at the study or outcome level), and how this information is to be used in any data synthesis.	7
Summary measures	13	State the principal summary measures (e.g., risk ratio, difference in means).	8
Synthesis of results	14	Describe the methods of handling data and combining results of studies, if done, including measures of consistency (e.g., I^2) for each meta-analysis.	8

For peer review only - http://bmjopen.bmj.com/site/about/guidelines.xhtml

PRISMA 2009 Checklist

Section/topic	#	Checklist item	Reported on page #
Risk of bias across studies	15	Specify any assessment of risk of bias that may affect the cumulative evidence (e.g., publication bias, selective reporting within studies).	8
Additional analyses	16	Describe methods of additional analyses (e.g., sensitivity or subgroup analyses, meta-regression), if done, indicating which were pre-specified.	n/a
**RESULTS**			
Study selection	17	Give numbers of studies screened, assessed for eligibility, and included in the review, with reasons for exclusions at each stage, ideally with a flow diagram.	9 + Fig 1
Study characteristics	18	For each study, present characteristics for which data were extracted (e.g., study size, PICOS, follow-up period) and provide the citations.	9 + Table 2 (p24)
Risk of bias within studies	19	Present data on risk of bias of each study and, if available, any outcome level assessment (see item 12).	9 + Table 4,6 (p26,p28)
Results of individual studies	20	For all outcomes considered (benefits or harms), present, for each study: (a) simple summary data for each intervention group (b) effect estimates and confidence intervals, ideally with a forest plot.	n/a + Table 5 (p27)
Synthesis of results	21	Present results of each meta-analysis done, including confidence intervals and measures of consistency.	9-12 + Table 6 (p28)
Risk of bias across studies	22	Present results of any assessment of risk of bias across studies (see Item 15).	9-12 + Table 6 (p28)
Additional analysis	23	Give results of additional analyses, if done (e.g., sensitivity or subgroup analyses, meta-regression [see Item 16]).	n/a
**DISCUSSION**			
Summary of evidence	24	Summarize the main findings including the strength of evidence for each main outcome; consider their relevance to key groups (e.g., healthcare providers, users, and policy makers).	13-14, 15
Limitations	25	Discuss limitations at study and outcome level (e.g., risk of bias), and at review-level (e.g., incomplete retrieval of identified research, reporting bias).	14-15
Conclusions	26	Provide a general interpretation of the results in the context of other evidence, and implications for future research.	16
**FUNDING**			

For peer review only

For peer review only - http://bmjopen.bmj.com/site/about/guidelines.xhtml

PRISMA 2009 Checklist

Funding	27	Describe sources of funding for the systematic review and other support (e.g., supply of data); role of funders for the systematic review.	17

From: Moher D, Liberati A, Tetzlaff J, Altman DG, The PRISMA Group (2009). Preferred Reporting Items for Systematic Reviews and Meta-Analyses: The PRISMA Statement. PLoS Med 6(7): e1000097. doi:10.1371/journal.pmed1000097

For more information, visit: www.prisma-statement.org.

For peer review only

For peer review only - http://bmjopen.bmj.com/site/about/guidelines.xhtml
